# Supplementary material for: DNA barcoding for elasmobranch diversity assessment in Thailand: Its advantages and limitations
Source: PLoS One. 2025 Oct 31;20(10):e0334640. doi: 10.1371/journal.pone.0334640 (PMC12578239; doi:10.1371/journal.pone.0334640)
Supplement: S1 Table — (PDF) [file pone.0334640.s001.pdf]

S1 Table. Lists of internal primers used in the study

**Species**	**Primers (5' → 3')**
**COI**	
Centrophorus	
Cen_CO_1L	CCTGTGGCAATTAACCGTTG
Cen_CO_1H	TGGCTAGGTCTACGGATGCT
Cen_CO_2L	CCTCCCTCCCTCCTGTTACT
Cen_CO_2H	AATTATTCCGAAGCCGGGTA
Platyrhina psomadakisi	
Pla_CO_1L	GGCTTCAACACCAGCAGAAG
Pla_CO_1H	TAATCTTTGGTGCCTGAGCA
Pla_CO_2L	CTCCTGCAGGGTCAAAGAAG
Pla_CO_2H	ATAATTGGTTCCCCGGACAT
Glaucostegus	
Gla_CO_1L	ATTATTCCGAAGCCCGGTAG
Gla_CO_1H	GCTTCCGCTGGAGTTGAG
Gla_CO_2L	CTCCCGTTGGGATAGCAATA
Gla_CO_2H	CCCCCAGCAATTTCTCAATA
Gla_CO_3L	CCTGCTAAACCAAGGAAGTG
Gla_CO_3H	GCAATTGGTTTACTTGGCTTC
Rhinobatos	
RhiCOI_L1	GCCATCTTACCTGTGGCAAT
RhiCOI_R1	TCCAGCGGAGGCTAGTAGAA
RhiCOI_L2	GCACTGGCCTTAGTCTGCTT
RhiCOI_R2	GGTAGAGGATGGGGTCTCCT
RhiCOI_L3	GGCTGGAACAGGCTGAAC
RhiCOI_R3	GCCAAGTAGTCCGATTGCTA
**ND2**	
Hexatrygon	
Hex_ND2_1L	CCCCCAATTATTACCATTACTATCC
Hex_ND2_1H	GGAGGGGGTAAAGTTGGAGT
Hex_ND2_2L	TTGCCCTCGCACTAAAAATC
Hex_ND2_2H	ATCCGGATAAGGGAGGGAGT
Hex_ND2_3L	AATTGCTCACATTGGCTGAA
Hex_ND2_3H	TGGGGGTTAGTGGTAGTAGGG
Cruriraja andamanica	
Cru_ND2_1L	TGAACCCCCTTGTATTATCTATCC
Cru_ND2_1H	CGAATGGGGCTAGTTTCTGT
Cru_ND2_2L	TGCCTGAATAACAGGACAATG
Cru_ND2_2H	GATCGAGAGTAGTGGTGATTTGG
Cru_ND2_3L	TTCATTTTGCCCCTAACCTG
Cru_ND2_3H	AGGAGGATGGATAGTGAAGTGG
Centrophorus	
Cen_ND2_1L	AAACTCTTGGTGCTTCCGATT
Cen_ND2_1H	GATTAGGCCCGTGGTAAGGT
Cen_ND2_2L	CGCCACACTCGTAACCATT
Cen_ND2_2H	TCCGAGAGAGAGCAGGGTAA
Cen_ND2_3L	CTACTCCCCCAACCTTACCC

Species	Primers (5’ → 3’)
Cen_ND2_3H	GCTTTGAAGGCTTTTGGTCT
Platyrhina psomadakisi<br>Pla_ND2_1H	CCTCCTCAACCCCCAATAAT
Pla_ND2_1L	TGAACCCCTTAATCCTATTTATTTT
Pla_ND2_2H	GGAAACCTGAAAGTGGTGGA
Pla_ND2_2L	TGCACTTTTGATTGCCAGAA
Pla_ND2_3H	CAATCGCACATCTAGGCTGA
Pla_ND2_3L	TCGAAATTGATGCGGTAGTG
Carcharhinus longimanus<br>CL_ND2_1L	TAGTGTGGCAGAAGTTGGGT
CL_ND2_1H	AAAGCTTTTGGGCCCATACC
CL_ND2_2L	CCGAGGTTTGCGATTGATG
CL_ND2_2H	AACACCATCCACGAGCAGTA
CL_ND2_3L	GGGTTGGATGGTTGGGTTTT
CL_ND2_3H	CATTGGAGGATGAGGAGGACT
Bythaelurus lutarius<br>Blu_ND2L_1	CCCTCCGTGTTTCCTATTACAC
Blu_ND2R1	CACCCTCCGATTATTGTTGA
Blu_ND2L_2	CGCTTGAACCTCAGGTGAAT
Blu_ND2R2	GTTGTGGCGTAACAAAGACG
Blu_ND2L_3	CGCCCCTAACCTAACCCTAC
Blu_ND2R3	CTGAGTTGCATTCAGAAGATGTG

PCR preparation and amplification

- DNA extraction and quality checking
  DNA is extracted from all samples using the commercial QIAGEN DNeasy Kit. The quality of the extracted DNA is assessed by electrophoresis on a 1% agarose gel. Their concentrations are measured using a Nanodrop (NanoVueTM) spectrophotometer.
  - If the extracted DNA samples show a single clear band at the top of the gel, they are selected for Nanodrop analysis to determine their concentrations.
  - If electrophoresis reveals fragmented bands, smearing, or no visible band, the samples are considered to be of poor quality. They are still analyzed using the Nanodrop to record their concentrations. Due to DNA degradation, PCR preparation for these samples is carried out separately.
- PCR preparation
  - For samples with good DNA quality, PCR cocktails are prepared as following:
    a reaction total volume of 25 ul using 5-7 ng of DNA template, 2 µl (0.2 mM) dNTP, 2.5 µl 10xPCR buffer, 1 µl (0.5 µM) for each primer, 1 µl (2 mM) MgCl2, 0.05 µl (5 unit/µl) Taq DNA polymerase, and deionized water

    COI primers (Ward et al. 2005)
    FishF1 (5′– TCA ACC AAC CAC AAA GAC ATT GGC AC –3′)
    FishR1 (5′– TAG ACT TCT GGG TGG CCA AAG AAT CA –3′)

    ND2 primers (Naylor et al. 2012)

ILEM (5' AAG GAG CAG TTT GAT AGA GT 3')
ASNM (5' AAC GCT TAG CTG TTA ATT AA 3')

- For samples with poor DNA quality, the PCR cocktails are initially prepared similarly to high-quality samples. Nevertheless, the amount of DNA template is increased to 15-20 ng, and the amount of deionized water is decreased to make a total reaction volume of 25 µl. The same primers are used as those for high-quality DNA samples. If the PCR products for COI fragments do not show clear band or yield no band, then the alternative primer pairs are tested.

  COI
  FishF2 (5' TCG ACT AAT CAT AAA GAT ATC GGC AC 3')
  FishR2 (5' ACT TCA GGG TGA CCG AAG AAT CAG AA 3')

For ND2, the internal primers (developed from unpublished document by Khudamrongsawat et al.) are used in combination with the universal primers (Naylor et al. 2012) as following:

ND2
ILEM (5' AAG GAG CAG TTT GAT AGA GT 3') with
RVintND2R (5' AAA GCA CTT GGG TTG CAT TC 3')

ASNM (5' AAC GCT TAG CTG TTA ATT AA 3') with
RVintND2R FWintND2R (5' CCA CAA AAA TTA ACT CAA TCA CCA 3')

However, if the samples could not be amplified, the primers listed in Table S1 are used for the corresponding taxa.

- PCR amplification
  - Regular PCR profile:
    an initial denaturation at 94°C for 3 minutes (min)
    followed by 35 cycles of
    denaturing at 94°C for 50 seconds (s)
    annealing at 50°C for 2 min
    extension at 72°C for 90 s
    a final extension at 72°C for 6 min.
  - PCR profile for difficult samples, a touchdown PCR profile is performed.
    an initial denaturation at 94°C for 3 min
    followed by 5 cycles of
    denaturing at 94°C for 30 s
    annealing at 48°C for 60 s
    extension at 72°C for 90 s
    then followed by 30 cycles of
    denaturing at 94°C for 30 s
    annealing at 50°C for 60 s
    extension at 72°C for 90 s
    a final extension at 72°C for 10 min
  - Samples that could not be amplified by any of these profiles were discarded.

References

Naylor GJP, Caira JN, Jensen K, Rosana KAM, White WT, Last PR. A DNA sequence-based approach to the identification of shark and ray species and its implications for global elasmobranch diversity and parasitology. B Am Mus Nat Hist. 2012;367:1-262.

Ward RD, Zemlak TS, Innes BH, Last PR, Hebert PDN. DNA barcoding Australia's fish species. Philos T R Soc B. 2005;360:1847-1857.
