# Supplementary material for: DNA barcoding for elasmobranch diversity assessment in Thailand: Its advantages and limitations
Source: PLoS One. 2025 Oct 31;20(10):e0334640. doi: 10.1371/journal.pone.0334640 (PMC12578239; doi:10.1371/journal.pone.0334640)
Supplement: S2 Table — Genetic comparisons of the samples in this study were based on COI and ND2 gene fragments, using reference sequences from the NCBI and BOLD databases. Phylogenetic tree analyses were performed using the Maximum Likelihood method. Initial species identifications, based on morphological characteristics, are not presented. Abbreviations: GOT = Gulf of Thailand; AN = Andaman Sea; U = unable to amplify sequences; NM = sequence did not match with any data; NF = species not found in the database. (PDF) [file pone.0334640.s002.pdf]

S2 Table. GenBank accession numbers of samples.

Sample ID	Consensus species	COI sequences	Best match (% identity)		ND2 sequences	NCBI Best match (% identity)
			NCBI	BOLD		
PNA-S-01	*Echinorhinus brucus*	–	U	U	PQ238751	*Echinorhinus* sp. (99.90%)
F021224	*Squalus hemipinnis*	–	U	U	PQ238750	NM
SM1	*Squalus montalbani*	OR395274	*Squalus montalbani* (97.51%)	*Squalus* sp. (99.54%)	–	U
CG1	*Centrophorus uyato*	–	U	U	OR400892	*Cen. uyato* (100%)
CG2	*Centrophorus uyato*	OR395257	*Centrophorus granulosus* (99.48%)/ *Cen. zeehaani* (99.48%)/ *Cen. uyato* (99.48%)	*Cen. zeehaani* (99.48%)	OR400893	*Cen. uyato* (100%)
CG3	*Centrophorus uyato*	–	U	U	OR400894	*Centrophorus uyato* (100%)
CG4	*Centrophorus uyato*	–	U	U	OR400895	*Centrophorus uyato* (100%)
EF2	*Etmopterus fusus*	OR391898	NM	NM	OR400903	*Etmopterus splendidus* (99.71%)
F021222	*Orectolobus leptolineatus*	–	U	U	PQ238749	*Orectolobus* cf. *leptolineatus* (99.62%)
SK-S-37	*Chiloscyllium hasseltii*	PQ517033	*Chiloscyllium hasseltii* (100%)/ *Ch. griseum* (100%)	*Chiloscyllium hasseltii* (100%)/ *Ch. griseum* (100%)	–	U
SNI-S-01	*Chiloscyllium hasseltii*	PQ517032	*Chiloscyllium hasseltii* (100%)	*Ch.* cf. *hasseltii* (100%)/ *C. griseum* (100%)	–	U
Tr-S-13	*Chiloscyllium hasseltii*	–	*Chiloscyllium hasseltii* (99.85%)	*Ch. hasseltii* (99.84%)	PP975131	U
ST-S-24	*Chiloscyllium indicum*	PP940169	U	U	PP975132	*Ch. griseum* (100%)
ST-S-25	*Chiloscyllium indicum*	PP940170	*Chiloscyllium indicum* (100%)	*Ch. indicum* (100%)	PP975133	*Ch. indicum* (100%)
ST-S-26	*Chiloscyllium indicum*	PP940171	*Chiloscyllium indicum* (100%)	*Ch. indicum* (100%)	PP975134	*Ch. indicum* (100%)
C.gri-04	*Chiloscyllium griseum*	PQ517028	*Chiloscyllium indicum* (100%)	*Ch. indicum* (100%)	–	*Ch. indicum* (100%)

Sample ID	Consensus species	COI sequences	Best match (% identity)		ND2 sequences	NCBI Best match (% identity)
			NCBI	BOLD		
RN-S-08	*Chiloscyllium griseum*	PQ517030	*Chiloscyllium hasseltii* (99.82%)	*Ch. griseum* 99.85%)	–	*Ch. griseum* (99.87%)
PK-S-05	*Chiloscyllium griseum*	PP940182	*Chiloscyllium hasseltii* (99.82%)	*Chiloscyllium hasseltii* 99.82%)/ *Ch. griseum* 99.85%)	PP975139	U
F021417	*Chiloscyllium punctatum*	PP940173	*Chiloscyllium punctatum* (100%)	*Ch. punctatum* (99.85%)	PP975135	*Ch. punctatum* (100%)
F021453	*Chiloscyllium punctatum*	PP940174	*Chiloscyllium punctatum* (100%)	*Ch. punctatum* (99.85%)	–	U
RY-S-02-2	*Chiloscyllium punctatum*	PP940175	*Chiloscyllium punctatum* (100%)	*Ch. punctatum* (100%)	PP975136	*Ch. punctatum* (99.71%)
ST-S-02	*Chiloscyllium punctatum*	PP940176	*Chiloscyllium punctatum* (99.85%)	*Ch. punctatum* (99.85%)	PQ040218	U
ST-S-09	*Chiloscyllium punctatum*	PP940177	*Chiloscyllium punctatum* (99.85%)	*Ch. punctatum* (99.85%)	–	U
ST-S-10	*Chiloscyllium punctatum*	PP940178	*Chiloscyllium punctatum* (99.85%)	*Ch. punctatum* (99.85%)	–	U
ST-S-44	*Chiloscyllium punctatum*	PP940179	*Chiloscyllium punctatum* (100%)	*Ch. punctatum* (100%)	PP975137	*Ch. punctatum* (100%)
Tr-S-12	*Chiloscyllium punctatum*	–	U	U	PP975138	*Ch. punctatum* (99.90%)
LS	*Stegostoma tigrinum*	PP940180	*Stegostoma fasciatum* (99.85%)	*S. fasciatum* (100%)	–	U
PK-S-157	*Stegostoma tigrinum*	–	U	U	PQ238731	*S. fasciatum* (99.47%)
PK-S-85	*Isurus oxyrinchus*	PQ192068	*Isurus oxyrinchus* (98.77%)	*I. oxyrinchus* (98.76%)	–	U
SK-S-20	*Atelomycterus marmoratus*	PP940119	*Atelomycterus marmoratus* (100%)	*A. marmoratus* (100%)	PQ238732	*A. marmoratus* (98.37%)
SK-S-21	*Atelomycterus marmoratus*	PP940120	*Atelomycterus marmoratus* (99.85%)	*A. marmoratus* (99.85%)	PQ238733	*A. marmoratus* (98.28%)
Blu1	*Bythaelurus lutarius*	OR104936	*Bythaelurus hispidus* (99.19%)	*B. hispidus* (99.17%)	MW143049	NM, NF
Blu3	*Bythaelurus lutarius*	OR104937	*Bythaelurus hispidus* (99.02%)	*B. hispidus* (99.03%)	MW143051	NM, NF
Blu5	*Bythaelurus lutarius*	OR104938	*Bythaelurus hispidus* (99.35%)	*B. hispidus* (99.35%)	MW143050	NM, NF
Blu7	*Bythaelurus lutarius*	–	U	U	MW143055	NM, NF

Sample ID	Consensus species	COI sequences	Best match (% identity)		ND2 sequences	NCBI Best match (% identity)
			NCBI	BOLD		
Blu8	*Bythaelurus lutarius*	–	U	U	MW143056	NM, NF
Blu9	*Bythaelurus lutarius*	–	U	U	MW143053	NM, NF
Lman	*Iago mangalorensis*	OR391911	*Iago* sp. (99.21%)	*Iago* sp. (100%)	OR400914	NM, NF
Tr-S-18	*Mustelus stevensi*	PP940131	*Mustelus lenticulatus* (100%)	*Mustelus* sp. 2 (100%)/ *M. lenticulatus* (100%)	PQ238742	*M. stevensi* (99.71%)
NK-S-06	*Carcharhinus amboinensis*	OR591439	*Carcharhinus amboinensis* (100%)	*Car. amboinensis* (100%)	PQ238739	*Car. amboinensis* (99.90%)
RY-S-01	*Carcharhinus brevipinna*	PP940121	*Carcharhinus brevipinna* (100%)	*Car. brevipinna* (100%)	PQ238734	*Car. brevipinna* (99.81%)
SK-S-17	*Carcharhinus brevipinna*	PP940122	*Carcharhinus brevipinna* (100%)	*Car. brevipinna* (100%)	PQ238735	*Car. brevipinna* (100%)
ST-S-14	*Carcharhinus brevipinna*	PP940123	*Carcharhinus brevipinna* (100%)	*Car. brevipinna* (100%)	PQ040221	*Car. brevipinna* (100%)
ST-S-15	*Carcharhinus brevipinna*	PP940124	*Carcharhinus brevipinna* (100%)	*Car. brevipinna* (100%)	PQ040222	*Car. brevipinna* (99.89%)
ST-S-16	*Carcharhinus brevipinna*	PP940125	*Carcharhinus brevipinna* (100%)	*Car. brevipinna* (100%)	–	U
PK-S-86	*Carcharhinus falciformis*	PP940126	*Carcharhinus falciformis* (99.85%)	*Car. brevipinna* (99.85%)/ *Car. falciformis* (99.85%)	PQ238736	*Car. falciformis* (100%)
PK-S-87	*Carcharhinus falciformis*	PQ192067	*Carcharhinus falciformis* (100%)	*Car. brevipinna* (100%)/ *Car. falciformis* (100%)	PQ238737	*Car. falciformis* (100%)
PK-S-88	*Carcharhinus falciformis*	PP940127	*Carcharhinus falciformis* (100%)	*Car. falciformis* (100%)	PQ238738	*Car. falciformis* (100%)
Tr-S-17	*Carcharhinus lecas*	PP940128	*Carcharhinus leucas* (100%)	*Car. leucas* (100%)	–	U
F021386	*Carcharhinus limbatus*	–	U	U	PQ238748	*Car. limbatus* (99.90%)
CL1	*Carcharhinus longimanus*	–	U	U	OR400896	*Car. longimanus* (99.90%)
CL2	*Carcharhinus longimanus*	–	U	U	OR400897	*Car. longimanus* (100%)
RY-S-02	*Carcharhinus melanopterus*	PP940129	*Carcharhinus melanopterus* (100%)	*Car. melanopterus* (100%)	PQ238740	*Car. melanopterus* (99.33%)

Sample ID	Consensus species	COI sequences	Best match (% identity)		ND2 sequences	NCBI Best match (% identity)
			NCBI	BOLD		
MUSC22072301	*Carcharhinus sorrah*	OR395260	*Carcharhinus sorrah* (99.41%)	*Car. sealei* (100%)/ *Car. sorrah* (100%)	OR400901	*Car. sorrah* (100%)
MUSC22072302	*Carcharhinus sorrah*	OR395261	*Carcharhinus sorrah* (99.55%)	*Car. sealei* (100%)/ *C. sorrah* (100%)	OR400902	*Car. sorrah* (100%)
Csor3	*Carcharhinus sorrah*	–	U	U	OR400900	*Car. sorrah* (99.81%)
Csor4	*Carcharhinus sorrah*	–	U	U	OR400899	*Car. sorrah* (100%)
Csor5	*Carcharhinus sorrah*	–	U	U	OR400898	*Car. sorrah* (100%)
ST-S-04	*Carcharhinus sorrah*	PP940130	*Carcharhinus sorrah* (100%)	*Car. sealei* (100%)/ *Car. sorrah* (100%)	PQ238741	*Car. sorrah* (99.90%)
ST-S-18	*Carcharhinus sorrah*	PP940131	*Carcharhinus sorrah* (100%)	*Car. sealei* (100%)/ *Car. sorrah* (100%)	–	U
Tr-S-01	*Carcharhinus sorrah*	PQ835806	*Carcharhinus sorrah* (100%)	*Car. sealei* (100%)/ *Car. sorrah* (100%)	–	U
Tr-S-02	*Carcharhinus sorrah*	PQ835807	*Carcharhinus sorrah* (100%)	*Car. sealei* (100%)/ *Car. sorrah* (100%)	PQ040218	*Car. sorrah* (99.86%)
PK-S-89	*Prionace glauca*	PQ192069	*Prionace glauca* (100%)	*P. glauca* (100%)	PQ238743	*P. glauca* (100%)
PK-S-90	*Prionace glauca*	PQ192070	*Prionace glauca* (100%)	*P. glauca* (100%)	PQ238744	*P. glauca* (100%)
PK-S-91	*Prionace glauca*	PQ192071	*Prionace glauca* (100%)	*P. glauca* (100%)	PQ238745	*P. glauca* (100%)
GA1	*Galeocerdo cuvier*	OR391899	*Galeocerdo cuvier* (100%)	*G. cuvier* (100%)	OR400904	*G. cuvier* (100%)
PK-S-171	*Galeocerdo cuvier*	–	U	U	PQ238730	*G. cuvier* (99.56%)
ST-S-20	*Sphyrna lewini*	PP940134	*Sphyrna lewini* (100%)	*S. lewini* (100%)	–	U
ST-S-21	*Sphyrna lewini*	PP940135	*Sphyrna lewini* (100%)	*S. lewini* (100%)	–	U
Tr-S-16	*Sphyrna lewini*	PP940132	*Sphyrna lewini* (100%)	*S. lewini* (100%)	PQ238746	*S. lewini* (99.90%)
Tr-S-19	*Sphyrna lewini*	PP940133	*Sphyrna lewini* (100%)	*S. lewini* (100%)	PQ238747	*S. lewini* (100%)
THNHM-F022018	*Sphyrna mokarran*	OR395289	*Sphyrna mokarran* (100%)	*S. lewini* (100%)/ *S. mokarran* (100%)	OR400917	*S. mokarran* (99.81%)
ST-R-06	*Rhynchobatus australiae*	PQ145565	*Rhynchobatus australiae* (100%)	*Rh. australiae* (100%)	–	U
ST-R-17	*Rhynchobatus australiae*	PQ145567	*Rhynchobatus australiae* (100%)	*Rh. australiae* (100%)	–	U

Sample ID	Consensus species	COI sequences	Best match (% identity)		ND2 sequences	NCBI Best match (% identity)
			NCBI	BOLD		
Rhi	*Rhinobatos ranongensis*	OR395273	*Rhinobatos* cf. *borneensis* (99.69%)	NM	OR400916	NF, NM
GT1	*Glaucostegus younholeei*	OR391900	*Glaucostegus* sp. (100%)	*Glaucostegus* sp. (100%)	–	U
GT2	*Glaucostegus younholeei*	OR391901	*Glaucostegus* sp. (100%)	*Glaucostegus* sp. (100%)	–	U
GT3	*Glaucostegus younholeei*	OR391902	*Glaucostegus* sp. (100%)	*Glaucostegus granulosus* (100%)	–	U
GT4	*Glaucostegus younholeei*	OR391903	*Glaucostegus* sp. (100%)	*Glaucostegus* sp. (100%)	–	U
GT5	*Glaucostegus younholeei*	OR391904	*Glaucostegus* sp. (100%)	*Glaucostegus* sp. (100%)	–	U
GT6	*Glaucostegus younholeei*	OR391905	*Glaucostegus* sp. (100%)	*Glaucostegus* sp. (100%)	–	U
GT7	*Glaucostegus younholeei*	OR391906	*Glaucostegus* sp. (100%)	*Glaucostegus* sp. (100%)	–	U
GT8	*Glaucostegus younholeei*	OR391907	*Glaucostegus* sp. (100%)	*Glaucostegus* sp. (100%)	–	U
PP1	*Platyrhina psomadakisi*	OR395285	NF, NM	NM	–	U
PP2	*Platyrhina psomadakisi*	OR395286	NF, NM	NM	–	U
PP2	*Platyrhina psomadakisi*	OR395287	NF, NM	NM	–	U
PP4	*Platyrhina psomadakisi*	OR395288	NF, NM	NM	–	U
F201592	*Torpedo sinuspersici*	–	U	U	PQ382896	NM
F201593	*Torpedo sinuspersici*	–	U	U	PQ382897	NM
Ben3	*Benthobatis moresbyi*	OR395253	*Benthobatis moresbyi* (99.69%)	*Be. moresbyi* (99.68%)	OR400888	NF, NM
Ben4	*Benthobatis moresbyi*	OR395254	*Benthobatis moresbyi* (99.68%)	*Be. moresbyi* (99.67%)	OR400889	NF, NM
RN-R-06	*Narcine maculata*	PQ620196	*Narcine maculata* (98.73%)	*Na. prodorsalis* (99.84%)	–	U
RN-R-07	*Narcine maculata*	PQ620197	*Narcine maculata* (98.52%)	*Na. prodorsalis* (99.54%)	–	U
NAV	*Narcine prodorsalis*	OR395284	*Narcine maculata* (99.84%)/ *Narcine* sp. (99.52%), NF	*Na.* cf. *oculifer* (99.84%)	–	U
RN-R-05	*Narcine timlei*	PQ620198	*Narcine timlei* (97.26%)	*Na. brunnea* (99.85%)	–	U
Tr-R-33	*Orbiraja powelli*	PP968077	*Orbiraja powelli* (99.08%)	*O. powelli* (99.20%)	–	U

Sample ID	Consensus species	COI sequences	Best match (% identity)		ND2 sequences	NCBI Best match (% identity)
			NCBI	BOLD		
Cand2	*Cruriraja andamanica*	OR395255	NF, NM	NM	OR400891	NF, NM
Hexa	*Hexatrygon bickelli*	OR391909	*Hexatrygon bickelli* (100%)	*Hex. bickelli* (100%)	OR400906	*Hex. bickelli* (99.70%)
ST-R-38	*Gymnura poecilura*	PP968069	*Gymnura poecilura* (99.69%)	*G. poecilura* (99.69%)	PQ565809	NM
Tr-R-30	*Gymnura poecilura*	PP968070	*Gymnura poecilura* (99.85%)	*G. poecilura* (99.84%)	PQ565810	NM
MUSC220110	*Brevitrygon heterura*	OR395292	*Brevitrygon walga* (100%)	*Himantura walga* (100%)	–	U
MUSC220111	*Brevitrygon heterura*	OR395293	*Brevitrygon walga* (100%)	*H. walga* (100%)	OR400890	*B. walga* (99.90%)
MUSC22072306	*Brevitrygon heterura*	OR395262	*Brevitrygon walga* (99.84%)	*H. walga* (99.84%)	–	U
MUSC22072308	*Brevitrygon heterura*	OR395263	*Brevitrygon walga* (100%)/ *B. heterura* (100%)	*H. walga* (100%)	–	U
MUSC22072319	*Brevitrygon heterura*	OR395266	*Brevitrygon walga* (99.85%)	*H. walga* (99.85%)	–	U
B9-R-03	*Brevitrygon heterura*	PP968068	*Brevitrygon walga* (99.85%)	*B. walga* (100%)	–	U
ST-R-01	*Brevitrygon heterura*	PQ145564	*Brevitrygon heterura* (99.68%)	*B. heterura* (99.68%)	–	U
MUSC22072304	*Hemitrygon bennetti*	OR391908	*Hemitrygon bennetti* (100%)	*He. bennetti* (100%)	OR400905	*H.* (*Dasyatis*) *bennetti* (99.90%)
MUSC20240913	*Hemitrygon bennetti*	PQ897942	*Hemitrygon bennetti* (99.67%)	*He. Bennetti* (99.67%)	–	U
MUSC20241123	*Hemitrygon laosensis*	PQ897943	NM	NM	–	U
RN-R-18/ AN	*Himantula undulata*	PQ620195	*Himantura undulata*	*H. undulata* (100%)	–	U
MUSC22072303	*Maculabatis gerrardi*	OR395283	*Maculabatis gerrardi* (100%)	*M. gerrardi* (100%)	OR400915	*M. gerrardi* (99.52%)
ST-R-26	*Maculabatis gerrardi*	PQ145568	*Maculabatis macrura* (100%)/ *M. gerrardi* (100%)	*M. macrura* (100%)/ *M. gerrardi* (100%)	–	U
ST-R-27	*Maculabatis gerrardi*	PQ145569	*Maculabatis macrura* (100%)/ *M. gerrardi* (100%)	*M. macrura* (100%)	–	U
MP1	*Maculabatis pastinacoides*	OR395277	*Maculabatis pastinacoides* (100%)	*M. pastinacoides* (100%)	OR400907	NM

Sample ID	Consensus species	COI sequences	Best match (% identity)		ND2 sequences	NCBI Best match (% identity)
			NCBI	BOLD		
MP2	*Maculabatis pastinacoides*	OR395278	*Maculabatis pastinacoides* (100%)	*M. pastinacoides* (100%)	OR400908	NM
MP3	*Maculabatis pastinacoides*	OR395279	*Maculabatis pastinacoides* (99.84%)	*M. pastinacoides* (99.85%)	OR400909	NM
MP4	*Maculabatis pastinacoides*	OR395280	*Maculabatis pastinacoides* (99.68%)	*M. pastinacoides* (99.84%)	OR400910	NM
MP6	*Maculabatis pastinacoides*	OR395290	*Maculabatis pastinacoides* (99.84%)	*Himantura* sp. (99.84%)	OR400911	NM
MP7	*Maculabatis pastinacoides*	OR395281	*Maculabatis pastinacoides* (99.84%)	*M. pastinacoides* (99.84%)	OR400912	NM
MP8	*Maculabatis pastinacoides*	OR395282	*Maculabatis pastinacoides* (99.84%)	*M. pastinacoides* (99.84%)	OR400913	NM
ST-R-10	*Neotrygon caeruleopunctata*	PP967953	*Neotrygon kuhlii* (100%)	*N. kuhlii* (100%)	PQ589246	NM, NF
ST-R-11	*Neotrygon caeruleopunctata*	PP967954	*Neotrygon kuhlii* (100%)	*N. kuhlii* (100%)	PQ589246	NM, NF
ST-R-12	*Neotrygon caeruleopunctata*	PP967955	*Neotrygon kuhlii* (99.85%)/ *N. malaccensis* (99.85%)	*N. kuhlii* (100%)	PQ589246	NF, NM
RN-R-03	*Neotrygon caeruleopunctata*	PP967952	*Neotrygon kuhlii* (99.85%)	*N. kuhlii* (99.84%)	PQ589245	NF, NM
Tr-R-42	*Neotrygon caeruleopunctata*	PP967956	*Neotrygon kuhlii* (100%)	*N. kuhlii* (100%)	PQ589246	NF, NM
Tr-R-43	*Neotrygon caeruleopunctata*	PP967957	*Neotrygon kuhlii* (100%)/ *N. malaccensis* (100%)	*N. kuhlii* (100%)	PQ589247	NF, NM
Ch-R-10	*Neotrygon varidens*	PP967958	*Neotrygon trigonoides* (99.85%)/ *N. kuhlii* (99.85%)	*N.* cf. *kuhlii* (99.84%)/ *N. kuhlii* (99.84%)/ *N. trigonoides* (99.84%)	PQ589243	*N. kuhlii* (99.81%)
Ch-R-11	*Neotrygon varidens*	PP967959	*Neotrygon trigonoides* (99.85%)/ *N. kuhlii* (99.85%)	*N. kuhlii* (99.84%)	PQ589243	*N. kuhlii* (99.81%)
CTI-R-01	*Neotrygon varidens*	PP967960	*Neotrygon kuhlii* (100%)	*N. kuhlii* (100%)	PQ589243	*N. kuhlii* (100%)

Sample ID	Consensus species	COI sequences	Best match (% identity)		ND2 sequences	NCBI Best match (% identity)
			NCBI	BOLD		
RN-R-12	*Pateobatis jenkinsii*	PP968074	*Pateobatis jenkinsii* (100%)	*P. jenkinsii* (100%)	PV037693	*H. jenkinsii* (99.43%)
RN-R-13	*Pateobatis jenkinsii*	PP968075	*Pateobatis jenkinsii* (100%)	*P. jenkinsii* (100%)	–	U
RN-R-14	*Pateobatis jenkinsii*	PP968076	*Pateobatis jenkinsii* (99.70%)	*P. jenkinsii* (99.85%)	–	U
NK-R-23	*Pateobatis uarnacoides*	PP968071	*Pateobatis uarnacoides* (99.85%)	*H. uarnacoides* (99.84%)	PQ565811	*P.* (*Himantura*) *uarnacoides* (99.00%)
RN-R-17	*Pateobatis uarnacoides*	PP968072	*Pateobatis uarnacoides* (100%)	*H. uarnacoides* (100%)	–	U
MUSC2024081501	*Pateobatis uarnacoides*	PQ897944	*Pateobatis uarnacoides* (99.69%)	*H. uarnacoides* (99.69%)	–	U
PB-THNHM2024	*Pateobatis uarnacoides*	PQ897945	*Pateobatis uarnacoides* (99.85%)	*P. uarnacoides* (100%)	–	U
F021259	*Pteroplatytrygon violacea*	PQ620199	*Pteroplatytrygon violacea* (99.85%)	*P. violacea* (100%)	PQ382895	*P. violacea* (99.90%)
MUSC22091001	*Taeniura lymma*	OR395275	*Taeniura lymma* (100%)	*Ta. lymma* (100%)	OR400918	*Ta. lymma* (99.42%)
MUSC22091002	*Taeniura lymma*	OR395276	*Taeniura lymma* (100%)	*Ta. lymma* (100%)	OR400919	*Ta. lymma* (99.52%)
Bre1-MUSC220109	*Telatrygon biasa*	OR395267	*Telatrygon* (*Dasyatis*) *zugei* (100%)	*T. zugei* (100%)	OR400920	*T.*(*Dasyatis*) *zugei* (99.14%)
Bre16-MUSC22072316	*Telatrygon biasa*	OR395268	*Telatrygon* (*Dasyatis*) *zugei* (100%)	*T. zugei* (100%)	OR400921	*T.* (*Dasyatis*) *zugei* (99.62%)
Bre18-MUSC22072318	*Telatrygon biasa*	OR395269	*Telatrygon* (*Dasyatis*) *zugei* (100%)	*T. zugei* (100%)	OR400922	*T.* (*Dasyatis*) *zugei* (99.33%)
Bre-20MUSC22072320	*Telatrygon biasa*	OR395270	*Telatrygon* (*Dasyatis*) *zugei* (99.38%)	*T. zugei* (99.69%)	OR400923	*T.* (*Dasyatis*) *zugei* (99.90%)
PK-R-191	*Telatrygon crozieri*	PP973883	*Telatrygon zugei* (100%)	*T. zugei* (100%)	PQ589239	*Telatrygon* (*Dasyatis*) *zugei* (98.66%)
PK-R-221	*Telatrygon crozieri*	PP973884	*Telatrygon zugei* (100%)	*T. zugei* (100%)	PQ589240	*Telatrygon* (*Dasyatis*) *zugei* (98.66%)
PK-R-228	*Telatrygon crozieri*	PP973885	*Telatrygon zugei* (100%)	*T. zugei* (100%)	PQ589241	*Telatrygon* (*Dasyatis*) *zugei* (98.66%)

Sample ID	Consensus species	COI sequences	Best match (% identity)		ND2 sequences	NCBI Best match (% identity)
			NCBI	BOLD		
G0005060	*Urogymnus lobistoma*	PQ626155	*Urogymnus lobistoma* (99.38%)	*H. uarnocoides* (99.38%)	–	U
PKR329	*Myliobatis hamlyni*	–	U	U	PQ628108	*Myliobatis tobijei* (99.43%)
B9-R-03	*Aetobatus ocellatus*	–	U	U	PQ565807	*Aetobatus ocellatus* (99.52%)
NK-S-08	*Aetobatus ocellatus*	PP968067	*Aetobatus ocellatus* (100%)	*A. ocellatus* (100%)/ *A. narinari* (100%)	PQ565808	*A. ocellatus* (99.52%)
PN-R-B	*Rhinoptera jayakari*	PP968073	*Rhinoptera jayakari* (100%)	*Rhinoptera* sp. (100%)/ *R. jayakari* (100%)	PQ565812	*R. jayakari* (99.89%)
Mtar	*Mobula tarapacana*	PP967859	*Mobula tarapacana* (100%)	*M. tarapacana* (100%)	PP967859	*M. tarapacana* (100%)
