# Supplementary material for: DNA barcoding for elasmobranch diversity assessment in Thailand: Its advantages and limitations
Source: PLoS One. 2025 Oct 31;20(10):e0334640. doi: 10.1371/journal.pone.0334640 (PMC12578239; doi:10.1371/journal.pone.0334640)
Supplement: Attachment — Submitted filename: review 1.pdf [file pone.0334640.s003.pdf]

The manuscript (PONE-D-25-18018), titled “DNA barcoding for elasmobranch diversity assessment in Thailand: its advantages and limitations” is an interesting work. However, the revisions are necessary to improve the clarity, depth, and contextualization of findings. Detailed comments and suggestions are provided below, organized by section.

Please, adding line numbers would help reviewers provide more precise feedback

Abstract
"mean for species identification" should be "means of species identification."
"exercise cautions" should be "exercise caution."
Suggest professional English editing for clarity and flow.

Methodological Clarifications

- Mention the primers used in this study
- DNA quantification method: Mention how DNA concentration was measured (e.g., Nanodrop, Qubit?).
- Threshold justification: Explain why 98% identity and 95% coverage were chosen as cutoffs (e.g., precedent in literature).
- Glaucostegus exception: The lowering of query coverage to 60% should be explained more clearly is this due to database fragment length?
- Model selection: The model used for phylogenetics (TIM2+F+I+G4) is specified, but the alignment software used to generate sequence alignments is not mentioned.
- Outgroups: No mention of which outgroups were used (if any) in tree construction — important for rooting and interpretation.
- Voucher information: Though storage locations are mentioned, include how voucher IDs were assigned and whether they are linked to GenBank entries.

The study relies heavily on publicly available databases
(BOLD, NCBI), many of which contain mislabeled or outdated entries. This issue is discussed, but the authors might consider emphasizing the importance of curating verified local reference libraries.

The Results and Discussion
Consider separating detailed species-level data into Supplementary Materials.
Use summary tables/figures for clarity
(e.g., show cases of taxonomic conflict or cryptic species with quick-reference tables).
