# Supplementary material for: DNA barcoding for elasmobranch diversity assessment in Thailand: Its advantages and limitations
Source: PLoS One. 2025 Oct 31;20(10):e0334640. doi: 10.1371/journal.pone.0334640 (PMC12578239; doi:10.1371/journal.pone.0334640)
Supplement: Attachment — Submitted filename: Review_PONE-D-25-18018_Muhala.pdf [file pone.0334640.s004.pdf]

PLOS ONE

DNA barcoding for elasmobranch diversity assessment in Thailand: its advantages and limitations

--Manuscript Draft--

**Manuscript Number:**	PONE-D-25-18018
**Article Type:**	Research Article
**Full Title:**	DNA barcoding for elasmobranch diversity assessment in Thailand: its advantages and limitations
**Short Title:**	DNA barcoding for elasmobranch diversity assessment in Thailand
**Corresponding Author:**	Wansuk Senanan, Ph.D.<br>Burapha University<br>Chonburi, Chonburi THAILAND
**Keywords:**	sharks, rays, batoid, COI, ND2, genetic variation
**Abstract:**	The assessment of elasmobranch biodiversity in Thailand benefits greatly from the application of DNA barcoding, which helps mitigate the challenge posed by a shortage of expert taxonomists. Fragments of COI and ND2 mitochondrial DNA were examined, and the strength and weaknesses of these two markers were compared. In this study, a total of 28 shark species and 32 batoid species were identified. Many species could be confidently identified as their morphological characteristics aligned with DNA barcodes. However, several exceptions were recognized. The absence of reference sequences for rare species presented a challenge for species verification, and the misidentification of reference sequences, as well as changes in species names due to taxonomic revisions, added complexity when comparing DNA barcoding sequences. Conflicts between morphology and genetics were also observed. While intraspecific genetic variation based on both DNA barcodes generally indicated 0-2% variation, this metric could not always be used for species delimitation. This was particularly true for species displaying low genetic variation among closely related species and species where cryptic diversity remained hidden and yet to be uncovered. In such cases, the morphological characteristics of the samples served as a primary mean for species identification. Despite these challenges, DNA barcoding remains an invaluable tool for biodiversity assessment, especially in light of the shortage of skilled experts and identification of products made of vulnerable species. However, it is essential to exercise cautions and be aware of these complexities in its application.
**Order of Authors:**	Jenjit Khudamrongsawat, Ph.D.
	Tassapon Krajangdara
	Thadsin Panithanarak, Ph.D.
	Ratima Karuwancharoen
	Wanlada Klangnurak, Ph.D.
	Pattarapon Promnun
	Wansuk Senanan, Ph.D.
**Additional Information:**	
**Question**	**Response**
**Financial Disclosure**<br><br>Enter a financial disclosure statement that describes the sources of funding for the work included in this submission. Review the submission guidelines for detailed requirements. View published research articles from *PLOS ONE* for specific	Yes

Powered by Editorial Manager® and ProduXion Manager® from Aries Systems Corporation

examples.<br><br>This statement is required for submission and **will appear in the published article** if the submission is accepted. Please make sure it is accurate.<br><br>**Funded studies**<br>Enter a statement with the following details:<br>• Initials of the authors who received each award<br>• Grant numbers awarded to each author<br>• The full name of each funder<br>• URL of each funder website<br>• Did the sponsors or funders play any role in the study design, data collection and analysis, decision to publish, or preparation of the manuscript?<br><br>Did you receive funding for this work?	
Please add funding details.<br>as follow-up to "**Financial Disclosure**<br><br>Enter a financial disclosure statement that describes the sources of funding for the work included in this submission. Review the submission guidelines for detailed requirements. View published research articles from *PLOS ONE* for specific examples.<br><br>This statement is required for submission and **will appear in the published article** if the submission is accepted. Please make sure it is accurate.<br><br>**Funded studies**<br>Enter a statement with the following details:<br>• Initials of the authors who received each award<br>• Grant numbers awarded to each author<br>• The full name of each funder<br>• URL of each funder website<br>• Did the sponsors or funders play any role in the study design, data collection and analysis, decision to publish, or preparation of the manuscript?	This project is funded by National Research Council of Thailand (NRCT-N25A650485).

Powered by Editorial Manager® and ProduXion Manager® from Aries Systems Corporation

Did you receive funding for this work?"	
Please select the country of your main research funder (please select carefully as in some cases this is used in fee calculation).<br>as follow-up to "**Financial Disclosure**<br><br>Enter a financial disclosure statement that describes the sources of funding for the work included in this submission. Review the submission guidelines for detailed requirements. View published research articles from *PLOS ONE* for specific examples.<br><br>This statement is required for submission and **will appear in the published article** if the submission is accepted. Please make sure it is accurate.<br><br>**Funded studies**<br>Enter a statement with the following details:<br>• Initials of the authors who received each award<br>• Grant numbers awarded to each author<br>• The full name of each funder<br>• URL of each funder website<br>• Did the sponsors or funders play any role in the study design, data collection and analysis, decision to publish, or preparation of the manuscript?<br><br>Did you receive funding for this work?"	THAILAND - TH
**Competing Interests**<br><br>Use the instructions below to enter a competing interest statement for this submission. On behalf of all authors, disclose any competing interests that could be perceived to bias this work—acknowledging all financial support and any other relevant financial or non-financial competing interests.	The authors have declared that no competing interests exist.

This statement is required for submission and **will appear in the published article** if the submission is accepted. Please make sure it is accurate and that any funding sources listed in your Funding Information later in the submission form are also declared in your Financial Disclosure statement.<br><br>View published research articles from *PLOS ONE* for specific examples.<br><br>**NO authors have competing interests**<br><br>Enter: *The authors have declared that no competing interests exist.*<br><br>**Authors with competing interests**<br><br>Enter competing interest details beginning with this statement:<br><br>*I have read the journal's policy and the authors of this manuscript have the following competing interests: [insert competing interests here]*<br><br>* typeset	
**Ethics Statement**<br><br>Enter an ethics statement for this submission. This statement is required if the study involved:<br><br>• Human participants<br>• Human specimens or tissue<br>• Vertebrate animals or cephalopods<br>• Vertebrate embryos or tissues<br>• Field research<br><br>Write "N/A" if the submission does not require an ethics statement.<br><br>General guidance is provided below. Consult the submission guidelines for detailed instructions. **Make sure that all information entered here is included in the Methods section of the manuscript.**	Permission for animal use was granted by MU-IACUC (permission MUSC62-025-489) and Burapha University (IACUC 002/2566).

**Format for specific study types**<br><br>**Human Subject Research (involving human participants and/or tissue)**<br>• Give the name of the institutional review board or ethics committee that approved the study<br>• Include the approval number and/or a statement indicating approval of this research<br>• Indicate the form of consent obtained (written/oral) or the reason that consent was not obtained (e.g. the data were analyzed anonymously)<br><br>**Animal Research (involving vertebrate animals, embryos or tissues)**<br>• Provide the name of the Institutional Animal Care and Use Committee (IACUC) or other relevant ethics board that reviewed the study protocol, and indicate whether they approved this research or granted a formal waiver of ethical approval<br>• Include an approval number if one was obtained<br>• If the study involved *non-human primates*, add *additional details* about animal welfare and steps taken to ameliorate suffering<br>• If anesthesia, euthanasia, or any kind of animal sacrifice is part of the study, include briefly which substances and/or methods were applied<br><br>**Field Research**<br><br>Include the following details if this study involves the collection of plant, animal, or other materials from a natural setting:<br>• Field permit number<br>• Name of the institution or relevant body that granted permission	
**Data Availability**<br><br>Authors are required to make all data underlying the findings described fully available, without restriction, and from the time of publication. PLOS allows rare exceptions to address legal and ethical concerns. See the PLOS Data Policy and FAQ for detailed information.	No - some restrictions will apply

Powered by Editorial Manager® and ProduXion Manager® from Aries Systems Corporation

A Data Availability Statement describing where the data can be found is required at submission. Your answers to this question constitute the Data Availability Statement and **will be published in the article**, if accepted.<br><br>**Important:** Stating 'data available on request from the author' is not sufficient. If your data are only available upon request, select 'No' for the first question and explain your exceptional situation in the text box.<br><br>Do the authors confirm that all data underlying the findings described in their manuscript are fully available without restriction?	
**Describe where the data may be found in full sentences. If you are copying our sample text, replace any instances of XXX with the appropriate details.**<br><br>• If the data are **held or will be held in a public repository**, include URLs, accession numbers or DOIs. If this information will only be available after acceptance, indicate this by ticking the box below. For example: *All XXX files are available from the XXX database (accession number(s) XXX, XXX.).*<br>• If the data are all contained **within the manuscript and/or Supporting Information files**, enter the following: *All relevant data are within the manuscript and its Supporting Information files.*<br>• If neither of these applies but you are able to provide **details of access elsewhere**, with or without limitations, please do so. For example:<br><br>*Data cannot be shared publicly because of [XXX]. Data are available from the XXX Institutional Data Access / Ethics Committee (contact via XXX) for researchers who meet the criteria for access to confidential data.*<br><br>*The data underlying the results presented in the study are available*	We included all the Genbank accession numbers for the nucleotide sequences in the Supporting Information file. These accession number will be released upon publication being published (https://www.ncbi.nlm.nih.gov/).

from (include the name of the third party and contact information or URL).<br>• This text is appropriate if the data are owned by a third party and authors do not have permission to share the data.<br><br>* typeset	
Additional data availability information:	Tick here if the URLs/accession numbers/DOIs will be available only after acceptance of the manuscript for publication so that we can ensure their inclusion before publication.

Powered by Editorial Manager® and ProduXion Manager® from Aries Systems Corporation

Manuscript
Click here to access/download;Manuscript;text_1_2025-03-21_Khudamrongsa

DNA barcoding for elasmobranch diversity assessment in Thailand: its advantages and [illegible]itations

Jenjit Khudamrongsawat[1], Tassapon Krajangdara[2], Thadsin Panithanarak[3], Ratima Karuwancharoen[3], Wanlada Klangnurak[4], Pattarapon Promnun[5], Wansuk Senanan[6**]

[1]Department of Biology, Faculty of Science, Mahidol University, Rama VI Road, Rajathewi District, Bangkok, 10400, Thailand
[2]66/27 Soi 5, Thep Thani Village, Moo 2, Rasada Subdistrict, Muang District, Phuket Province 83000, Thailand
[3]Institute of Marine Science, Burapha University, 169 Long Had Bangsaen Rd, Saen Suk, Chon Buri District, Chon Buri 20131, Thailand
[4]Department of Animal Production Technology and Fishery, School of Agricultural Technology, King Mongkut's Institute of Technology Ladkrabang, Bangkok, 10520, Thailand
[5]Geneus Genetics Co.,Ltd, 37/1 Sukhumvit 101/1, Bang Chak, Phra Kanong, Bangkok, 10260, Thailand
[6]Department of Aquatic Science, Burapha University, 169 Long Had Bangsaen Rd, Saen Suk, Chon Buri District, Chon Buri 20131, Thailand

******corresponding author

Abstract

The assessment of elasmobranch biodiversity in Thailand benefits greatly from the application of DNA barcoding, which helps mitigate the challenge posed by a shortage of expert taxonomists. Fragments of COI and ND2 mitochondrial DNA were examined, and the strength and weaknesses of these two markers were compared. In this study, a total of 28 shark species and 32 batoid species were identified. Many species could be confidently identified as their morphological characteristics aligned with DNA barcodes. However, several exceptions were recognized. The absence of reference sequences for rare species presented a challenge for species verification, and the misidentification of reference sequences, as well as changes in species names due to taxonomic revisions, added complexity when comparing DNA barcoding sequences. Conflicts between morphology and genetics were also observed. While intraspecific genetic variation based on both DNA barcodes generally indicated 0-2% variation, this metric could not always be used for species delimitation. This was particularly true for species displaying low genetic variation among closely related species and species where cryptic diversity remained hidden and yet to be uncovered. In such cases, the morphological characteristics of the samples served as a primary mean for species identification. Despite these challenges, DNA barcoding remains an invaluable tool for biodiversity assessment, especially in light of the shortage of skilled experts and identification of products made of vulnerable species. However, it is essential to exercise cautions and be aware of these complexities in its application.

Keywords: sharks, rays, batoid, COI, ND2, genetic variation

Introduction

The Southeast Asian region (SEA) boasts a remarkable array of elasmobranch species in which the ongoing discovery of new, undescribed species is adding to our appreciation of the extraordinary diversity of this under-studied group [1, 2]. To safeguard these taxa in the region, the Southeast Asian Fisheries Development Center (SEAFDEC) has been actively encouraging SEA countries to assess their elasmobranch diversity so as to better formulate international conservation policies. Among SEA nations, Thailand is one of chondrichthyan biodiversity hotspots. This is attributable to the country's expansive marine ecosystem covering nearly 3,000 kilometers of coastlines, spanning both the Indo-West Pacific region and the Indian Ocean. The geological dynamics and intricate evolutionary history further contribute to this biodiversity wealth [3, 4]. The latest records unveil over 180 species (across 12 orders and 43 families), including some apparently distinct, yet morphologically similar lineages are yet to be confirmed as full species [5]. Evident disparities exist in elasmobranch diversity between the Gulf of Thailand and the Andaman Sea. The species composition within the Gulf of Thailand tends to closely resemble that of the western Pacific region, whereas the Andaman Sea shows resemblances to species prevalent in the east Indian Ocean. Notably, the Andaman Sea boasts a higher number of elasmobranch species compared to the Gulf of Thailand. This discrepancy is further underscored by the presence of Holocephali species exclusively recorded in the Andaman Sea [5, 6]. The escalating species count is a result of enhanced survey efforts and the integration of molecular tools for species identification. Despite ongoing surveys and the submission of DNA barcode information to the NCBI database and Barcode of Life Database (BOLD), Thailand's remarkable elasmobranch diversity remains poorly in the existing literature [7–10]. Available genetic information, though scant, poses many questions regarding taxonomic diversity in which the high levels of genetic differentiation that could not be ignored [11].

The evaluation of elasmobranch diversity hinges on the utilization of both morphological and molecular tools, with DNA barcoding especially the cytochrome c oxidase subunit I (COI) and the nicotinamide adenine dehydrogenase 2 (ND2) gene fragments, gaining prominence [7, 12–15]. DNA barcoding facilitates the differentiation of cryptic species that are difficult to distinguish based on appearance alone [16]. Even species with extensive and apparently continuous distributions may exhibit genetic variations that have implications for disparate evolutionary trajectories [17, 18]. Specifically, ND2 has shown superiority in uncovering genetic variations in cryptic sharks and is strongly recommended for use in chondrichthyan DNA barcoding [7, 19]. While DNA barcoding offers a powerful and expeditious tool for assessing biodiversity, it has proven challenging to establish clear criteria for genetic differentiation between species, particularly for non-taxonomists. In many instances, genetic variations within elasmobranchs fall within a 2% range at the intraspecific level [13, 15, 20–22]. However, exceptions do exist [13, 15, 19, 23, 24]. These strengths and weaknesses of DNA barcoding for biodiversity assessment in various taxa have also been acknowledged [25–33]. Nonetheless, as Thailand has an urgent need for improved biodiversity assessment, the adoption of DNA

barcoding is a worthwhile endeavor. Genetic insights into species have yielded significant advances in shaping the Thailand National Plan of Action for the Conservation and Management of Sharks (NPOA-Sharks, Thailand). This aligns with the nation's economic and development agenda in accordance with the United Nations' 2030 Sustainable Development Goals (SDGs).

This research's primary objectives were to evaluate the extent of DNA barcoding application based on COI and ND2 fragments for both taxonomists and non-taxonomists in elasmobranch biodiversity assessment and to create DNA barcoding databases of elasmobranch diversity in Thailand. The outcomes hold the potential to provide valuable contributions to the regional conservation efforts by shedding light on Thailand's unique biological resources.

Methods

- Tissue sampling locations and collections

Samples were obtained from various sources, encompassing research vessels, fish markets, and fishing harbors situated in both the Gulf of Thailand and the Andaman Sea over the period from 2013 to 2023. These specimens were photographed and underwent subsequent species identification by T. Krajangdara. The identification process relied on the guidance provided in Krajangdara et al. [5], Last et al. [34], Krajangdara [35], and Ebert et al. [36]. Instances where characteristics proved intricate or displayed unclear attributes were categorized as unidentified species, designated with "cf." or "sp." To definitively classify these cases, molecular analysis was performed. Muscle or fin clips were excised from the specimens and conserved in 95% ethanol, subsequently being frozen at -20°C at Burapha University, Mahidol University, King Mongkut's Institute of Technology Ladkrabang, and the Natural History Museum of Thailand. In cases involving sizeable, expensive, or live individuals, photographs were taken as records, with the focus being solely on acquiring tissue samples. To ensure the future viability of the research, selected samples were designated as voucher specimens and retained at these institutions: the Phuket Marine Biological Center, Burapha University, King Mongkut's Institute of Technology Ladkrabang, and Natural History Museum of Thailand.

- Molecular examination

DNA samples were extracted using commercial Extraction Kit (QIAGEN─DNeasy kit). DNA barcoding based on cytochrome c oxidase subunit I (COI) and NADH dehydrogenase subunit 2 (ND2) were amplified following the protocols by Ward et al. [12] and Naylor et al. [7]. Degraded tissue samples, especially those obtained before 2020, could not be amplified using universal primers, and so new primers were designed for investigation. List of primers used in this study were listed in Supporting information (S1 File). Polymerase chain reactions (PCR) were carried out with a reaction volume of 25 ul using 5-7 ng of DNA template, 2 µl (0.2 mM) dNTP, 2.5 µl 10xPCR buffer, 1 µl (0.5 µM) for each primer, 1 µl (2 mM) $MgCl_2$, 0.05 µl (5 unit/µl) Taq DNA polymerase, and deionized water. PCR cycles were performed with an initial denaturation at 94°C for 3 min, followed by 35 cycles of 94°C for 50 s, annealing at 50°C for 2 min, and extension at 72°C for 90 s, and a final extension at 72°C for 6 min. For samples posing difficulty in amplification, a touchdown PCR profile was performed including an initial denaturation at 94°C for 3 min, followed by 5 cycles of 94°C for 30 s, annealing at 48°C for 60 s, and extension at 72°C for 90 s, and then followed by 30 cycles of 94°C for 30 s, annealing at

50°C for 60 s, and extension at 72°C for 90 s, and a final extension at 72°C for 10 min. PCR products were checked by electrophoresis using 1.5% agarose gel and purified using commercial Gel/PCR purification kit (QIAGEN━QIAquick PCR purification kit). Sequences were sent for Sanger sequencing using an ABI 3730 automated sequencer. All sequences were edited, translated into amino acid using MEGA 11 [37].

- Data analyses

Species were confirmed through a dual process involving both morphological identification and cross-referencing with reference sequences available in the National Center for Biotechnology Information (NCBI) and the Barcode of Life Database (BOLD). Taxonomic name changes, particularly in the family Dasyatidae, were also recognized [38]. The differences in the generic names of species in the genera previously named as *Himantura* and *Dasyatis*, were not treated as different from the newly assigned names. In instances where matches were inconclusive, a thorough assessment was conducted using supplementary information, notably morphology and geographic data. In cases of persistent uncertainty, the designation “cf.” was attributed to those particular samples. The validation of species through molecular means relied on a similarity scoring system, demanding a minimum of 95% query coverage and a similarity level of 98% or higher. This methodology utilized the BLAST algorithm within NCBI, as well as the Identification Engine embedded in BOLDSYSTEMS. Nevertheless, COI fragments from our [illegible]ples did not overlap with most *Glaucostegus* COI sequences in the public database, preventing the application of a 95% query coverage threshold. Instead, a minimum coverage of 60% or higher was used. A separate file was created for *Glaucostegus* tree construction. Following these comprehensive validations, the genetic sequences were duly submitted to the NCBI database, contributing to the broader body of scientific knowledge and facilitating further research and insights into the studied species (S2 file).

Furthermore, to elucidate the relationships between the samples under investigation and the reference data, phylogenetic trees were constructed. The best-fit substitution model for the sequence alignment was selected using IQ-TREE. ModelFinder [39]. This method, integrated within IQ-TREE V2.3.6, was employed to identify the best substitution model based on the Bayesian Information Criterion (BIC), which was TIM2+F+I+G4. Bayesian inference of phylogeny was conducted using MrBayes (v3.2.7) [40]. A Markov Chain Monte Carlo (MCMC) analysis was executed in MrBayes with a total of 1,000,000 generations. The posterior distribution was sampled every 100 generations. Four chains were used to improve exploration of the posterior distribution. After the analysis, 25% of the samples were discarded as burn-in to eliminate the influence of the starting state. Convergence diagnostics, including the standard deviation of split frequencies and effective sample sizes (ESS), were assessed to ensure reliability of the analysis. The consensus tree was generated and visualized by Figtree V1.4.4 with posterior probabilities representing the clade support [41].

These trees offered a visual representation of the evolutionary placements for both sharks and batoids. The genetic distances were quantified using the Kimura-2-Parameter (K2P) method, gauging the variations of intra- and inter- specific diversity of species and genera of samples in this study. For this analysis, the reference sequences of unidentified species of reference sequences were excluded. Because several species in this study were represented by a small

sample size, sequences in NCBI and BOLD databases under the same scientific names were used to enable intraspecific comparison.

Results

There were 153 samples with successful amplification of either COI or ND2 fragments or both. The two DNA barcoding fragments offered different advantages (Table 1). COI fragments showed better rate of amplification success than ND2, while ND2 provided more characteristics for comparison. A larger number of ND2 sequences could not be matched with any references in the database, whereas most COI sequences found their matches, but some matched sequences attributed to more than one species and were therefore inconclusive. Nevertheless, the matching results did not indicate correct species identification because conclusive species identification was conducted using morphological examination. The genetic data only showed possible identification if only DNA sequences were available.

Table 1. Comparisons of COI and ND2 fragments as DNA barcoding for elasmobranch species identification from 153 samples.

criteria	COI	ND2
number of sequences of successful amplification	130	99
length of fragments obtained (bp)	520-676	970-1047
number of sequences NOT matched with any reference sequences or matched unidentified species	9	31
number of sequences matched with multiple species	26	0

With the combined use of morphological and genetic data, the samples in this study unveiled a total of 28 shark species and 32 batoid species. The process of arriving at consensus species heavily relied on morphological traits, with genetic data serving a supplementary role. Identification to species was congruent when assessed through both morphological and genetic examination when allowing for intraspecific genetic variations of no more than 2% (Table 2; Figs 1– 4). Phylogenetic analysis of a sample assigned to *Orectolobus leptolineatus* on morphological criteria matched an unidentified *Orectolobus* sequence (Fig 2A).

Figure 1. Phylogenetic tree of sharks based on 654 bp of COI fragments; (A) overall relationships of sharks in this study (B) phylogenetic tree of sharks in the order Carcharhiniformes; bold = samples in this study; number on lines indicating bootstrap values.

Figure 2. Phylogenetic tree of sharks based on 1044 bp of ND2 fragments; (A) overall relationships of sharks in this study (B) phylogenetic tree of sharks in the order Carcharhinoformes; bold = samples in this study; number on lines indicating bootstrap values.

Figure 3. Phylogenetic tree of batoids based on 652 bp of COI fragments; (A) overall relationships of batoids in this study (B) phylogenetic tree of batoids in the family Dasyatidae; (C) phylogenetic tree of batoids in the genus *Glaucostegus*, bold = samples in this study,

sequences MW431029, MW431018 identified as *G. younholeei* (Habib and Islam 2021).; number on lines indicating bootstrap values.

Figure 4. Phylogenetic tree of batoids based on 1044 bp of ND2 fragments; (A) overall relationships of batoids in this study (B) phylogenetic tree of sharks in the family Dasyatidae; bold = samples in this study; number on lines indicating bootstrap values.

Table 2. Species identification based on morphological and genetic characteristics.

Morphological identification	genetic identification based on COI and/or ND2 (best match)	Phylogenetic tree placement	consensus species	problem
**SHARKS**				
ECHINORHINIFORMES				
Echinorhinus brucus	*Echinorhinus* sp.	*Echinorhinus* sp.	*E. brucus*	Insufficient information for comparison
SQUALIFORMES				
Squalus hemipinnis	not matched	*S. brevirostris*	*S. hemipinnis*[*1]	Insufficient information for comparison
Squalus montalbani	*Squalus* sp.	*Squalus* sp. A	*S. montalbani*	Insufficient information for comparison
Centrophorus uyato	*C. granulosus/ C. zeehaani/ C. uyato*	*Cen. uyato*	*C. uyato*[*2]	Taxonomic revision
Etmopterus fusus	not matched	Not group with any species (COI); *E. splendidus* (ND2)	*E. fusus*	Discordance between morphology and genetics
ORECTOLOBIFORMES				
Orectolobus leptolineatus	*O.* cf. *leptolineatus*	*O. leptolineatus*	*O. leptolineatus*	None
Chiloscyllium griseum	*C. griseum*	*C. hasselti/ C. griseum*	*C. griseum*	None
Chiloscyllium hasselti	*C. hasselti/ C. griseum*	*C. hasselti/ C. griseum*	*C. hasselti*	Cryptic morphological similarity
Chiloscyllium indicum	*C. indicum*	*C. indicum*	*C. indicum*	None
Chiloscyllium punctatum	*C. punctatum*	*C. punctatum*	*C. punctatum*	None
Stegostoma tigrinum	*S. fasciatum*	*S. fasciatum*	*S. tigrinum*[*3]	None
LAMNIFORMES				
Isurus oxyrinchus	*I. oxyrinchus*	*I. oxyrinchus*	*I. oxyrinchus*	None
CHACHARHINIFORMES				
Atelomycterus marmoratus	*A. marmoratus*	*A. marmoratus*	*A. marmoratus*	None
Bythaelurus lutarius	*B. hispidus*	*B. hispidus* (COI); not grouped with any species (ND2)	*B. lutarius*	Discordance between morphology and genetics
Iago mangalorensis	*Iago* sp.	*Iago* sp. A	*I. mangalorensis*	Insufficient information for comparison
Mustelus stevensi	*Mustelus* sp./ *M. lenticulatus/ M. stevensi*	*M. lenticulatus* (COI); *M. stevensi* (ND2)	*M. stevensi*	Cryptic morphological similarity
Carcharhinus amboinensis	*C. amboinensis*	*C. amboinensis*	*C. amboinensis*	None
Carcharhinus brevipinna	*C. brevipinna*	*C. brevipinna*	*C. brevipinna*	None
Carcharhinus falciformis	*C. falciformis/ C. brevipinna*	*C. falciformis*	*C. falciformis*	Cryptic morphological similarity
Carcharhinus leucas	*C. leucas*	*C. leucas*	*C. leucas*	None
Carcharhinus limbatus	*C. limbatus*	*C. limbatus*	*C. limbatus*	None

Morphological identification	genetic identification based on COI and/or ND2 (best match)	Phylogenetic tree placement	consensus species	problem
Carcharhinus longimanus	*C. longimanus*	*C. longimanus*	*C. longimanus*	None
Carcharhinus melanopterus	*C. melanopterus*	*C. melanopterus*	*C. melanopterus*	None
Carcharhinus sorrah	*C. sorrah/ C. sealei*	*C. sorrah*	*C. sorrah*	Cryptic morphological similarity
Prionace glauca	*P. glauca*	*P. glauca*	*P. glauca*	None
Galeocerdo cuvier	*G. cuvier*	*G. cuvier*	*G. cuvier*	None
Sphyrna lewini	*S. lewini*	*S. lewini*	*S. lewini*	None
Sphyrna mokarran	*S. mokarran*	*S. mokarran*	*S. mokarran*	None
**BATOIDS**				
RHINOPRISTIFORMES				
Rhynchobatus australiae	*R. australiae*	*R. australiae*	*R. australiae*	None
Rhinobatos ranongensis	no matched	*Rhinobatos* cf. *borneensis*	*R. ranongensis*	Insufficient information for comparison
Glaucostegus younholeei	*Glaucostegus* sp. (later as *G. younholeei*)/ *G. granulosus*	*Glaucostegus* sp./ *G. granulosus*	*G. younholeei*[*4]	Cryptic morphological similarity
Platyrhina psomadakisi	not matched	not grouped with any species	*P. psomadakisi*	Insufficient information for comparison
TORPEDINIFORMES				
Torpedo sinuspersici	not matched	*Torpedo* sp./ *T. sinuspersici*	*T. sinuspersici*	Insufficient information for comparison
Benthobatis moresbyi	*B. moresbyi*	*B. moresbyi* (COI); not grouped with any species (ND2)	*B. moresbyi*	None
Narcine maculata	*N. maculata*	*N. maculata*	*N. maculata*	None
Narcine prodorsalis	*N. maculata/ Narcine* sp./ *N.* cf. *oculifer*	*Narcine* sp.	*N. prodorsalis*	Cryptic morphological similarity
Narcine timlei	*N. timlei/ N. brunnea*	*N. timlei*	*N. timlei*	Cryptic morphological similarity
RAJIFORMES				
Orbiraja powelli	*O. powelli*	*O. powelli*	*O. powelli*	None
Cruriraja andamanica	not matched	*Cruriraja triangularis* (COI); *C. hulleyi* (ND2)	*C. andamanica*	Insufficient information for comparison
MYLIOBATIFORMES				
Hexatrygon bickelli	*H. bickelli*	*H. bickelli*	*H. bickelli*	None
Gymnura poecilura	*G. poecilura*	*G. poecilura*	*G. poecilura*	None
Brevitrygon heterura	*B.* (*Himantura*) *walga*/ *B. heterura*	*B. walga* / *B. heterura*	*B. heterura*[*5]	Taxonomic revision

Morphological identification	genetic identification based on COI and/or ND2 (best match)	Phylogenetic tree placement	consensus species	problem
Hemitrygon bennetti	*H.* (*Dasyatis*) *bennetti*	*H. bennetti*	*H. bennetti*	None
Hemitrygon laosensis	not matched	not grouped with any species	*H. laosensis*	Insufficient information for comparison
Himantula undulata	*H. undulata*	*H. undulata*	*H. undulata*	None
Maculabatis gerrardi	*M. gerrardi/ M. macrura*	*M. macrura/ M. gerrardi*	*M. gerrardi*	Cryptic morphological similarity
Maculabatis pastinacoides	*M. pastinacoides*	*M. pastinacoides*	*M. pastinacoides*	None
Neotrygon caeruleopunctata	*N. kuhlii/ N. malaccensis/ N. caeruleopunctata*	*N. kuhlii / N. varidens/ N. caeruleounctata*	*Neotrygon caeruleopunctata* [*6]	Ttaxonomic revision
Neotrygon varidens	*N. trigonoides/ N. kuhlii/ N.* cf. *kuhlii*	*N. kuhlii/ N. trigonoides*	*N. varidens*[*6]	Taxonomic revision
Pateobatis jenkinsii	*P.* (*Himantura*) *jenkinsii*	*P. jenkinsii*	*P. jenkinsii*	None
Pateobatis uarnacoides	*P.* (*Himantura*) *uarnacoides*	*P. uarnacoides*	*P. uarnacoides*	None
Pteroplatytrygon violacea	*P. violacea*	*P. violacea*	*P. violacea*	None
Taeniura lymma	*T. lymma*	*T. lymma*	*T. lymma*	None
Telatrygon biasa	*T.* (*Dasyatis*) *zugei/ T. biasa*	*T.* (*Dasyatis*) *zugei* (COI); *T.* (*Dasyatis*) *zugei / T. biasa* (ND2)	*T. biasa*[*8]	Taxonomic revision
Telatrygon crozieri	*T.* (*Dasyatis*) *zugei*	*T.* (*Dasyatis*) *zugei / T. biasa*	*T. crozieri*[*7]	Taxonomic revision
Urogymnus lobistoma	*U. lobistoma*	*U. lobistoma*	*U. lobistoma*	None
Myliobatis hamlyni	*M. tobijei*	*M. tobijei*	*M. hamlyni*[*8]	Taxonomic revision
Aetobatus ocellatus	*A. ocellatus/ A. narinari*	*A. ocellatus*	*A. ocellatus*[*9]	Taxonomic revision
Rhinoptera jayakari	*R. jayakari*	*R. jayakari*	*R. jayakari*	None
Mobula tarapacana	*M. tarapacana*	*M. tarapacana*	*M. tarapacana*	None

*1 short ND2 fragment comparison = *S. hemipinnis*

*2 taxonomic revision—*C. zeehaani* = syn. of *C. uyato* often misidentified with *C. granulosus* [42]

*3 Listed as *Stegostoma fasciatum* in BOLD, listed as *S. fasciatum* and *S. tigrinum* in NCBI

*4 *Glaucostegus* sp. in GenBank described as *G. younholeei* sp. nov. in Habib and Islam [43]

*5 taxonomic revision and geographic distribution of *Brevitrygon* [38]

*6 taxonomic revision of *Neotrygon kuhlii* [44-45]

*7 taxonomic revision and new species erection [34]

*8 taxonomic revision and new species erection [46]

*9 taxonomic revision and new species erection [47]

Not all samples could be identified. Problems regarding species identification could be grouped into 4 categories.

1. Insufficient information for comparison/ lack of species registered in the database

Specific challenges emerged in cases where information on references was unavailable within established databases. In these instances, the primary criterion for species identification was based on morphological characteristics of the samples. These samples included rarely found species such as *Hemitrygon laosensis*, *Echinorhinus brucus*, *Squalus montalbani*, *Iago mangalorensis*, *Platyrhina psomadakisi*, *Torpedo sinuspersici*, and *Cruriraja andamanica*. Genetic data for some species are newly described such as *Rhinobatos ranongensis* were not available for public access. In these cases, we relied solely on morphology for species identification.

For *Squalus hemipinnis*, the reference sequences of which did not fully meet the sequence alignment criteria of this study, and did not align with any known species. Although the phylogenetic tree placed it in close proximity to *S. brevirostris*, there was a 2.5% genetic difference. An alignment of short fragments of ND2 of our specimen and sequences in the NCBI database (529 base pairs—not shown) listed under *S. hemipinnis* revealed their similarity. This information was omitted and not presented here. By combining morphological characteristics and partial DNA barcoding data, the consensus identification for this sample was determined to be *S. hemipinnis*.

2. Taxonomic revision/ redescription/ new species assignments

Taxonomic revisions present yet another challenge that impedes the comprehensive utilization of DNA barcodes in databases. Many of these barcodes were deposited prior to taxonomic revisions and have not been updated, resulting in classification of samples under different names. Such instances were observed in *Centrophorus uyato*, several species of rays in the family Dasyatidae and *Aetobatus ocellatus*, where they were matched with species bearing the names before revisions. Consensus as to species was drawn based on morphological characteristics and published distributional ranges of taxa in current literature. The names used when the samples were deposited were likely synonyms and require updating. Our DNA sequence data represented and comparable with recent DNA information deposited after the revision. Nevertheless, some revised taxa, namely *T. crozieri*, lacked references for their DNA barcodes. Given the unavailability of morphology data for these references, we attempted to establish DNA barcoding profiles that aligned with their corresponding morphological characteristics under the assigned names.

The samples of *Glaucostegus* in this study were previously recognized as *Glaucostegus* cf. *granulosus* due to their genetic similarity to this species but still showed morphological differences. Our DNA sequences matched an undescribed *Glaucostegus* sp. later named as a distinct species. The updated publication of the sequence information in the NCBI website allowed us to place the valid name on these samples as *G. younholeei* [43].

The reassignment of species in the genus *Myliobatis* clarified the presence of two closely related species, *M. hamlyni* and *M. tubijei*. *Myliobatis hamlyni* was previously considered a junior synonym of *M. tobijei* but later confirmed as a valid species. The name applied to

sequences of *M. tobijei* that matched with our sample were likely following the original designation, not subsequently updated. No sequences of *M. hamlyni* were available for comparison with our data. Based on morphological examination and distribution range, we conclude that the consensus species of this sample is *M. hamlyni*.

3. Cryptic morphological similarity

There were discrepancies in the labeling of sequences when comparing the NCBI and BOLD databases for a few species, such as *Chiloscyllium hasseltii*, which closely resembles *C. griseum*; *Carcharhinus falciformis*, misidentified as *C. brevipinna; C. sorrah*, misidentified as *C. seali*; and *Maculabatis gerrardi*, occasionally misidentified as *M. macrura*. These species pairs highly similar may be misidentified by non-specialists. For example, *C. hasseltii* and *C. griseum* are morphologically similar as adults but can be distinguished in the juvenile stage based on the presence of dark brown bands. Similarly, *M. gerrardi* and *M. macrura* both exhibit white spots [illegible] their bodies but differ in the distribution of these markings.

Some species are unfamiliar and sometimes misidentified as related species such as *Mustelus stevensi* occasionally misidentified as *M. lenticulatus*, *Narcine timlei* occasionally misidentified as *N. brunnea*. These samples exhibited distinct morphological characteristics from their congeners. In such cases, we placed reliance on the morphological characteristics of these species, recognizing the potential for mislabeling in the references. Furthermore, we cross-referenced synonyms of these species with available literature, giving priority to accepted names. Consequently, the consensus species identification remained consistent with the initially assigned species name.

Sequences under name *Narcine prodorsalis* were either not present or unavailable for public access in both NCBI and BOLD. However, our *N. prodorsalis* COI sequence matched with an undescribed *Narcine* sp. and one *N. maculata*. The phylogenetic placement of our sample and the other *Narcine* spp. showed that the *N. maculata* that matched our sequence was likely mislabeled because all other *N. maculata* reference sequences formed a distinct clade.

4. Discordance between morphology and genetics

We also encountered discrepancies between morphology and genetics as in the case of *Bythaelurus lutarius*. This species exhibited clear morphological differences from its sympatric counterpart, *B. hispidus*, and other congeners. However, their COI sequences aligned with that of *B. hispidus* (Fig 1B) while ND2 sequences did not aligned with any *Bythaelurus* species (Fig 2B). These morphological distinctions included the absence of papillae in the tongue and roof of the mouth, variations in head shape and differences in the origins of the dorsal fins [5]. As a result, a consensus identification could not be definitively established, and the specimens were provisionally assigned as *B. lutarius*.

Another challenging case was found in a sample morphologically identified as *Etmopterus fusus*. The COI sequence of this sample did not match any references in both NCBI and BOLD while its ND2 sequence matched an *E. splendidus* sample from Taiwan. Our sample from the Andaman Sea displayed clear morphological characteristics consistent with *E. fusus*, including the presence of four black bands above the anal fin continuing to the caudal fin. This feature distinguished it from *E. splendidus*, which displayed a different black band pattern in the

same position. The distribution ranges of these 2 species are clearly different with *E. fusus* in the Eastern Indian Ocean and northern Western Australia, and *E. splendidus* in the Northwestern Pacific Ocean. The single reference COI sequence of *E. fusus* which came from a sample in New Caledonia showed a genetic distance from our sample greater than 6% based on 659 bp. Based on the morphological characteristics and the collecting locality of our sample, the consensus identification was *E. fusus*.

The average intraspecific genetic distance for sharks and batoids across both gene fragments, COI and ND2, was about 1% (Table 3). However, the maximum genetic distance was much greater. In sharks, the highest COI intraspecific variation was observed between our *Etmopterus fusus* sample and the NCBI reference, while the greatest ND2 variation was observed in our *Echinorhinus brucus* sample and the reference. Among batoids, samples of *Brevitrygon imbricata* and *Aetobatus flagellum* presented highest intraspecific genetic distances for both genes. The average genetic differentiation among congeneric species exceeded 7% for both gene fragments. Additionally, *Himantura leoparda* and *Narcine* spp. showed COI intraspecific variation exceeding 4%.

Table 3. Genetic distance (K2P percentage) based on COI and ND2 of elasmobranch species.

**Levels of genetic comparison**	**Taxa**	**COI**			**ND2**		
		Mean (S.E.)	Min	Max	Mean (S.E.)	Min	Max
Variations among individual of the same species	Sharks	0.92 (0.26)	0.00	6.32	0.76 (0.21)	0.00	5.37
	Batoids	0.86 (0.13)	0.00	5.12	1.26 (0.31)	0.00	10.49
Variations among species of the same genus	Sharks	7.50 (0.54)	0.05	17.73	10.88 (0.57)	0.39	21.01
	Batoids	10.42 (0.45)	0.21	26.40	11.31 (0.59)	0.58	32.38

However, very low interspecific genetic distances were observed between our *Etmopterus fusus* sample and the NCBI reference of *E. splendidus* and between our *Mustelus stevensi* sample and the reference *M. lenticulatus*. Among batoids, very low interspecific genetic variation was observed in *Neotrygon kuhlii* and other *Neotrygon* spp. and also within the genus *Telatrygon*. Similarly, low genetic variation was observed among species of *Rhinoptera*, *Aetobatus*, *Mobula*, and *Myliobatis* in the NCBI reference sequences.

The distribution of K2P genetic distances in sharks and batoids revealed distinct differences between intra- and interspecific variation (Figs 5, 6). Over 80% of our samples fell within 2% genetic distance of their assigned species. However, interspecific genetic variation varied across both genes. Some genera, such as *Etmopterus* and *Echinorhinus*, showed highly similar sequences, likely due to limited available genetic data. In contrast, other genera, such as *Neotrygon*, *Telatrygon*, and *Aetobatus*, have undergone taxonomically revisions, and current scientific names of many sequences have not been updated.

Sequences from our samples of *Iago mangalorensis*, *Rhinobatos ranongensis*, *Cruriraja andamanica*, and *Hemitrygon laosensis*, did not match any published species since reference

sequences were unavailable. These DNA sequences showed distinct genetic diversity compared to their congeneric species.

Figure 5. Distribution of K2P genetic distance in sharks (A) COI, arrow pointing to *Etmopterus fusus*; (B) ND2, arrow pointing to *Echinorhinus brucus*.

Figure 6. Distribution of K2P genetic distance in batoids (A) COI, arrows pointing *to Narcine timlei*, *N. brunnea*, *Brevitrygon imbricata*; (B) ND2 arrows pointing to *Brevitrygon imbricata*, *Aetobatus flagellum*.

Discussion

The implementation of DNA barcoding significantly enhances the taxonomic clarification of cartilaginous fishes, offering an alternative to the intricate and time-consuming process of morphological examination for non-taxonomists [15, 48]. Among the well-recognized mitochondrial gene candidates serving as DNA barcodes for cartilaginous fishes, COI and ND2 stand out [7, 12, 13, 15, 20]. COI tends to demonstrate higher success rates in amplification compared to ND2 due to its shorter length. The design of shorter fragments within the ND2 region amplification process has bolstered its efficacy, providing additional and valuable insights for future application. It is important not to pass judgment on the superiority of one gene over the other, as each presents distinct advantages. COI has enjoyed a decade-long legacy as a DNA barcode for fishes [12, 13, 15, 21, 22], whereas the acceptance of ND2 as a DNA barcode for chondrichthyans came later [7, 49]. The extensive utilization of COI is mirrored by the larger repository of COI sequences available across databases in comparison to ND2 sequences. However, ND2 sequences offer the advantage of increased length, subsequently affording more characteristics for meaningful comparison. Consequently, a synergistic employment of both genes holds promise in the accurate assessment of elasmobranch diversity. The number of ND2 sequences available for comparison is fewer than COI. COI still serves as important DNA barcode but need serious taxonomic updating.

The utility of DNA barcoding has contributed to new species records [5, 22, 50. 51]. In this study, DNA barcoding clarified specimens initially identified as *Glaucostegus* cf. *granulatus* [5], confirming them as *Glaucostegus younholeei* [43]. Additionally, DNA barcoding aids in identifying cryptic species, leading to new species descriptions and taxonomic revisions [e.g. 52–58].

Despite the considerable promise of DNA barcodes, this study has revealed several limitations. The effectiveness of this tool is contingent upon the availability of data from verified species, which is absent or limited from, especially, deep-sea species. Because of the rarity of specimens, identification based on morphological criteria may be mistaken resulting in erroneous molecular sequence assignment. The description of a new species, *Glaucostegus younholeei* sp. nov. [43], provided a clear identification of samples of the guitarfish previously described as *Glaucostegus* cf. *granulatus* [5] although the reference sequences in NCBI have not been

updated and listed the species as *Glaucostegus* sp. The authors of the new species have included a reference to the description of their newly described species as an update, hence providing an official name for the taxon. In addition, the presence of synonyms for certain species, such as the zebra shark, has also presented a problem, with two names existing in the databases. Although a priority is accorded to the senior synonym, *Stegostoma tigrinum* [59], several sequences of this species are still listed as *S. fasciatum*.

The issue of cryptic morphological similarity, due to either specimen misidentification or failure to take account of taxonomic revision, also presents a significant challenge when utilizing public databases for species verification. In such cases, it becomes necessary to rely on the morphological characteristics of specimens to confirm species assignment. The landscape of taxonomic revisions within many taxa adds complexity to the direct utilization of DNA barcodes, as species names undergo alterations over time. For example, DNA barcodes of samples identified as *Centrophorus uyato* have been matched with different *Centrophorus* species due to recent revisions that revealed *C. granulosus* as a species complex [42, 52]. As a result, several COI of several species including *C. uyato* were erroneously labeled as *C. granulosus*. On the contrary, information on ND2 sequences is more recent and up-to-date serving as a better reference than COI. A similar situation was found in *Aetobatus ocellatus* that was previously grouped within the *A. narinari* species complex but later recognized as a valid species based on molecular evidence [47, 53]. Sequences of samples labeled as *A. narinari* prior to this taxonomic revision could actually represent *A. ocellatus*, emphasizing the need for caution when using such references.

The extensive revision of stingrays of the family Dasyatidae has led to significant changes in generic classification and the description of new species exemplified by *Brevitrygon* [38, 54], *Neotrygon* [11], and *Telatrygon* [55]. These species exhibit distinct morphological characteristics that serve as a primary mean of identification, albeit requiring the expertise of specialists for their precise differentiation. To accelerate the identification process, DNA profiles of these species should be established. This study demonstrated such genetic distinctions through DNA barcoding, which contributes to the improved assessment of biodiversity.

Several sharks and rays are still subject to taxonomic revision due to their morphological similarity and very wide distribution ranges. The *Squalus* species complex presents wide distribution range and similar morphological characteristics among congeners [56–58]. Some species are thought to be endemic to certain geographic locations, but their distribution is still unclear and requires further examination, which may result in further taxonomic revision [36]. Our *Squalus* samples provisionally identified as *S. hemipinnis* and *S. montalbani*, which did not match the reference sequences given for those taxa may be distinct lineages.

Discordance between morphology and the DNA barcode assigned to *Bythaelurus lutarius* provided difficulty in reaching consensus identification. Identification based on distinctive morphological characteristics revealed that all examined samples collected from the Thai Andaman Sea were of *B. lutarius* [5], clearly distinguishable from the only similar-looking

species found in the same region, *B. hispidus* [60], *B. stewarti* [61], *B. naylori* [62], *B. dawsoni* and *B. canescens* [63]. The lack of COI sequences of *B. lutarius* from the type locality and ND2 sequence of *B. hispidus* challenges the use of DNA barcoding for species confirmation. Because all *B. lutarius* sampled formed a monophyletic cluster based on longer ND2 fragments, clearly distinct from other catsharks whose sequences were available for comparison, they likely all represented the same species. With clear morphological distinction and uncertain genetic affinity within this genus, the samples are tentatively assigned as *B. lutarius*.

It has been long recognized that intraspecific genetic variations among metazoans generally fall within 2% [13, 15, 20–22]. However, low genetic variation among distinct but congeneric lineages is evident in *Mustelus* species [64]. The clear genetic differentiation between *M. stevensi* and *M. lenticulatus* [10], which allowed a correct consensus identification. On the contrary, certain species, such as *Etmopterus fusus*, *Squalus montalbani*, *Maculabatis gerrardi*, and *Narcine timlei*, display high genetic variation within species. This may arise from either cryptic diversity or misidentification of reference samples. Therefore, while a 2% genetic difference within species is helpful, it cannot serve as an absolute criterion for species delimitation. Comparisons of genetic differentiation based on COI and ND2 fragments showed slightly greater interspecific differences in ND2 compared to COI possibly due to the utilization of longer fragments in the analyses. Nevertheless, low genetic differentiation among distinct congeneric species may confound their recognition. For example, our *Etmopterus fusus* sample showed high COI sequence variation compared to reference *E. fusus* but similar a ND2 sequence compared to *E. splendidus*. Although both species are closely related and rarely available for thorough examinations [65], clear difference in their geographical distribution [66] helps identify our sample as *E. fusus*. While the influx of DNA barcoding sequences is expected to continue, its application should be approached with caution. Given the dearth of expert chondrichthyan taxonomists at both local and global scales, the utilization of molecular markers is important for facilitating biodiversity assessment [67]. The data gathered by our present study contribute both to the sample of genetic information from better-known species and new genetic information of several species not available in public databases. Despite this great benefit, the clarity of the species concept is crucial, and traditional taxonomic identification methods should not be disregarded [68].

Discordance between morphological and genetic characteristics and misidentification of closely related species may result from hybridization, which has been reported many times in elasmobranchs [69–74]. In such cases, DNA barcoding cannot resolve taxonomic identification and necessitates assessment of nuclear DNA. While hybridization and introgression have been documented in a few elasmobranch groups, many remain unexplored. Given that environmental changes may influence reproductive behaviors of these fishes, baseline data on genetics across different species are crucial for understanding these processes.

As elasmobranchs serve as resources for both local consumption and international export, prohibition of the use of these resources is impractical. On the other hand, regulation of fisheries

based on solid scientific foundation and correct species assignment will help sustain long-term exploitation of these resources. To achieve this level of regulation, fundamental knowledge on species and genetic diversity is important and requires thorough examination, especially of local populations [18]. DNA barcoding data then serve as references for the authentication of meat products [14, 75]. Limited data on biodiversity jeopardize populations of these fishes, especially those commonly used in the preparation of luxurious dishes and pharmaceutical products, which usually come from non-specified species and some of which are globally vulnerable species [76–80].

Acknowledgements

We would like to thank our assistants, P. Chanmuang. T. Meejan, P. Laongdee, K. Panpong, A. Pankhort, and P. Wongmanee, who contributed to sample collection and laboratory work. Several organizations and individuals provided specimens for this project, including the Marine Fisheries Research and Development Division and Inland Fisheries Research Development Division of the Department of Fisheries, the Thailand National Science Museum, C. Vidthayanon, and T. Vasinopas. This project is funded by National Research Council of Thailand (NRCT-N25A650485). Language editing was assisted by P. D. Round. Permission for animal use was granted by MU-IACUC (permission MUSC62-025-489) and Burapha University (IACUC 002/2566). The authors declare that they do not have any known financial interests or personal relationships that could have appeared to influence the work reported in this paper.

References

1. Weigmann S. Annotated checklist of the living sharks, batoids and chimaeras (Chondrichthyes) of the world, with a focus on biogeographical diversity: Annotated global checklist of Chondrichthyes. J Fish Biol. 2016;88(3):837–1037.
2. Ali A, Fahmi, Dharmadi, Krajangdara T, Khiok ALP. Biodiversity and habitat preferences of living sharks in the Southeast Asian region. Indones Fish Res J. 2018; 24: 133-140.
3. Voris HK. Maps of Pleistocene sea levels in Southeast Asia: shorelines, river systems and time durations. J Biogeogr. 2000;27(5):1153–67.
4. Costello MJ, Tsai P, Wong PS, Cheung AKL, Basher Z, Chaudhary C. Marine biogeographic realms and species endemicity. Nat Commun. 2017;8(1):1057.
5. Krajangdara T, Vidthayanon C, Ali A, Lim A, Sumontha M, Rodpradit R, et al. 2022. The Cartilaginous Fishes of Thailand and Adjacent Waters. Department of Fisheries, Ministry of Agriculture and Cooperatives. Chiang Mai, Thailand: Vanidakarnpim Publishing; 2022. Thai.
6. Krajangdara T, Fahmi, Ebert DA, Chaorattana C, and Khudamrongsawat J. Morphological and genetic evidence confirmed three new records of ghost shark species (Chimaeriformes) from the Andaman Sea of Thailand. Trop Nat His. 2021;21:218-233.
7. Naylor GJP, Caira JN, Jensen K, Rosana KAM, White WT, Last PR. A DNA sequence–based approach to the identification of shark and ray species and its implications for global elasmobranch diversity and parasitology. B Am Mus Nat Hist. 2012;367:1–262.

8. Khudamrongsawat J, Bhummakasikara B, Chansue N. Preliminary study of genetic diversity in the giant freshwater stingray, *Himantura chaophraya* (Batoidea: Dasyatidae) from the remnant populations in Thailand. Trop Nat His. 2017;17:175-180.
9. Last PR, Séret B, Naylor GJP. Description of *Rhinobatos ranongensis* sp. nov. (Rhinopristiformes: Rhinobatidae) from the Andaman Sea and Bay of Bengal with a review of its northern Indian Ocean congeners. Zootaxa. 2019;4576: 257–287.
10. White WT, Arunrugstichai S, Naylor GJP. Revision of the genus *Mustelus* (Carcharhiniformes: Triakidae) in the northern Indian Ocean, with description of a new species and a discussion on the validity of *M. walkeri* and *M. ravidus*. Mar Biodivers 2021; 51: 42.
11. Borsa P. Comments on "Annotated checklist of the living sharks, batoids and chimaeras (Chondrichthyes) of the world, with a focus on biogeographical diversity" (Weigmann, 2016). J Fish Biol. 2016;90: 1170-1175.
12. Ward RD, Zemlak TS, Innes BH, Last PR, Hebert PDN. DNA barcoding Australia's fish species. Philos Trans R Soc Lond B Biol Sci. 2005;360(1462):1847–57
13. Ward RD, Holmes BH, White WT, Last PR. DNA barcoding Australian chondrichthyans: results and potential uses in conservation. Mar Freshwater Res. 2008;59:57-71.
14. Holmes BH, Steinke D, Ward RD. Identification of shark and ray fins using DNA barcoding. Fish Res. 2009;95(2–3):280–288.
15. Cerutti-Pereyra F, Meekan MG, Wei N-WV, O'Shea O, Bradshaw CJA, Austin CM. Identification of rays through DNA barcoding: an application for ecologists. PLoS One. 2012;7(6):e36479.
16. Avise JC. Molecular Markers, Natural History, and Evolution. 2nd ed. Massachusetts: Sinauer, Sunderland; 2004.
17. Frankham R. Where are we in conservation genetics and where do we need to go? Conserv Genet. 2010;11(2):661–663.
18. Domingues RR, Hilsdorf AWS, Gadig OBF. The importance of considering genetic diversity in shark and ray conservation policies. Conserv Genet. 2018;19(3):501–525.
19. Moore ABM, White WT, Ward RD, Naylor GJP, Peirce R. Rediscovery and redescription of the smoothtooth blacktip shark, *Carcharhinus leiodon* (Carcharhinidae), from Kuwait, with notes on its possible conservation status. Mar Freshw Res. 2011;62(6):528.
20. Ward RD. DNA barcode divergence among species and genera of birds and fishes. Mol Ecol Resour. 2009;9(4):1077–1085.
21. Pavan-Kumar A, Gireesh-Babu P, Suresh Babu PP, Jaiswar AK, Prasad KP, Chaudhari A, et al. DNA barcoding of elasmobranchs from Indian Coast and its reliability in delineating geographically widespread specimens. Mitochondrial DNA. 2013;26(1):92–100.
22. Loh K-H, Lim K-C, Then AY-H, Adam S, Leung AJ-X, Hu W, et al. Advancing DNA barcoding to elucidate elasmobranch biodiversity in Malaysian waters. Animals. 2023;13(6):1002.
23. Kashiwagi T, Marshall AD, Bennett MB, Ovenden JR. The genetic signature of recent speciation in manta rays (*Manta alfredi* and *M. birostris*). Mol Phylogenet Evol. 2012;64(1):212–218.

24. Ariza AA, Adachi AMCL, Roque P, Hazin FHV, Vianna M, Rotundo MM, et al. DNA barcoding and species delimitation for dogfish sharks belonging to the Squalus genus (Squaliformes: Squalidae). Diversity. 2022;14(7):544.
25. Leray M, Knowlton N, Ho S-L, Nguyen BN, Machida RJ. GenBank is a reliable resource for 21st century biodiversity research. Proc Natl Acad Sci USA. 2019;116(45):22651–22656.
26. Pentinsaari M, Ratnasingham S, Miller SE, Hebert PDN. BOLD and GenBank revisited – Do identification errors arise in the lab or in the sequence libraries? PLoS One. 2020;15(4):e0231814.
27. Fontes JT, Vieira PE, Ekrem T, Soares P, Costa FO. BAGS: An automated Barcode, Audit & Grade System for DNA barcode reference libraries. Mol Ecol Resour. 2021;21(2):573–83.
28. Grant DM, Brodnicke OB, Evankow AM, Ferreira AO, Fontes JT, Hansen AK, et al. The future of DNA barcoding: Reflections from early career researchers. Diversity. 2021;13(7):313.
29. Mota M, Thomaz M, Fabrin C, Diamante NA, Alessandra, Filho HO, et al. DNA barcode is efficient for identifying bat species. J Mamm Evol. 2021;29(1):63–75.
30. Peláez ML, Horreo JL, García-Jiménez R, Valdecasas AG. An evaluation of errors in the mitochondrial COI sequences of Hydrachnidia (Acari, Parasitengona) in public databases. Exp Appl Acarol. 2022;86(3):371–384.
31. Baena-Bejarano N, Reina C, Martínez-Revelo DE, Medina CA, Tovar E, Uribe-Soto S, et al. Taxonomic identification accuracy from BOLD and GenBank databases using over a thousand insect DNA barcodes from Columbia. PLoS One. 2023;18: e0277379.
32. Lindner MF, Gonçalves LT, Bianchi FM, Ferrari A, Cavalleri A. Tiny insects, big troubles: a review of BOLD's COI database for Thysanooptera (Insecta). B Entomol Res. 2023;113: 703-715.
33. Ruedi M, Manzinallii J, Dietrich A, Vinciguerra L. Shortcoming of DNA barcodes: a perspective from the mammal fauna of Switzerland. Hystrix it J Mamm. 2023;34:54-61.
34. Last PR, White WT, de Carvalho MR, Séret B, Stehmann FW, Naylor GJP. Rays of the World. Ithaca: Cornell University Press; 2016.
35. Krajangdara T. The Cartilaginous Fishes (Sharks, Rays and Chimaeras) Found in Thai Waters and the Adjacent Areas. Marine Fisheries Research and Development Division, Department of Fisheries, Ministry of Agriculture and Cooperatives. Phuket: Phuketoffset Publishing; 2017. Thai.
36. Ebert, DA, Dando M, Fowler S. Sharks of the World – A Complete Guide. New Jersey: Princeton University Press; 2021.
37. Tamura K, Stecher G, Kumar S. MEGA11: Molecular Evolutionary Genetics Analysis version 11. Mol Biol Evol. 2021;38(7):3022–3027.
38. Last PR, Naylor GJP, Manjaji-Matsumoto BM. A revised classification of the family Dasyatidae (Chondrichthyes: Myliobatiformes) based on new morphological and molecular insights. Zootaxa. 2016a;4139(3):345–368.
39. Kalyaanamoorthy S, Minh BQ, Wong TKF, von Haeseler A, Jermiin LS. ModelFinder: fast model selection for accurate phylogenetic estimates. Nat Methods. 2017;14(6):587–589.

40. Ronquist F, Teslenko M, van der Mark P, Ayres DL, Darling A, Höhna S, et al. MRBAYES 3.2: Efficient Bayesian phylogenetic inference and model selection across a large model space. Syst Biol. 2012;61:539-542.
41. Rambaut A. FigTree v1. 3.1. Institute of Evolutionary Biology, University of Edinburgh, Edinburgh [Internet]. 2010.
42. Habib KA, Islam MJ. Description of a new species of giant guitarfish, *Glaucostegus younholeei* sp. nov. (Rhinopristiformes: Glaucostegidae) from the northern Bay of Bengal, Bangladesh. Zootaxa. 2021;4995(1):129–146.
43. White WT, Guallart J, Ebert DA, Naylor GJP, Veríssimo A, Cotton CF, et al. Revision of the genus *Centrophorus* (Squaliformes: Centrophoridae): Part 3—redescription of *Centrophorus uyato* (Rafinesque) with a discussion of its complicated nomenclatural history. Zootaxa. 2022;5155:1-50.
44. Borsa P, Shen K-N, Arlyza IS, Hoareau TB. Multiple cryptic species in the blue-spotted maskray (Myliobatoidei: Dasyatidae: *Neotrygon* spp.): An update. C R Biol. 2016;339(9–10):417–26.
45. Borsa P, Arlyza IS, Hoareau TB, Shen K-N. Diagnostic description and geographic distribution of four new cryptic species of the blue-spotted maskray species complex (Myliobatoidei: Dasyatidae; *Neotrygon* spp.) based on DNA sequences. J Oceanol Limnol. 2018;36(3):827–41.
46. White WT, Kawauchi J, Corrigan S, Rochel E, Naylor GJP. Redescription of the eagle rays Myliobatis hamlyni Ogilby, 1911 and M. tobijei Bleeker, 1854 (Myliobatiformes: Myliobatidae) from the East Indo-West Pacific. Zootaxa. 2015;3948(3):521–48.
47. White WT, Last PR, Naylor GJP, Jensen K, Caira JN. Clarification of *Aetobatus ocellatus* (Kuhl, 1823) as a valid species, and a comparison with *Aetobatus narinari* (Euphrasen, 1790) (Rajiformes: Myliobatidae). In: Last PR, White WT, Pogonoski JJ. (Eds.), Descriptions of new sharks and rays from Borneo. CSIRO Marine and Atmospheric Research Paper 032. 2010;32:141-164.
48. Gostel MR, Kress WJ. The expanding role of DNA barcodes: Indispensable tools for ecology, evolution, and conservation. Diversity (Basel). 2022;14(3):213.
49. Henderson AC, Reeve AJ, Jabado RW, Naylor GJP. A molecular-assisted taxonomic assessment of elasmobranch fishes (Chondrichthyes: Elasmobranchii) from south-eastern Aribia revisited. Zootaxa. 2025;5575:429-446.
50. Fernando D, Bown RMK, Tanna A, Gobiraj R, Ralicki H, Jockusch EL, et al. New insights into the identities of the elasmobranch fauna of Sri Lanka. Zootaxa. 2019;4585(2):201-238.
51. Zhang M, Shan B, Liu Y, Wang L, Yang C, Liu M, et al. A new record of Squalus montalbani (Chondrichthyes: Squaliformes: Squalidae) from the Nansha (Spratly) Island, South China Sea. AleP. 2023; 53: 51-57.
52. Dahl RB, Sigsgaard EE, Mwangi G, Thomsen PF, Jørgensen RD, Torquato F de O, et al. The sandy zebra shark: A new color morph of the zebra shark *Stegostoma tigrinum*, with a redescription of the species and a revision of its nomenclature. Copeia. 2019;107(3):524.
53. White WT, Ebert DA, Naylor GJP, Ho H, Clerkin P, Verissimo A, et al. Revision of the genus *Centrophorus* (Squaliformes: Centrophoridae): Part 1—Redescription of

Centrophorus granulosus (Bloch & Schneider), a senior synonym of *C. acus* Garman and *C. niaukang* Teng. Zootaxa. 2013;3752:035-072.
54. Richards VP, Henning M, Witzell W, Shivji MS. Species delineation and evolutionary history of the globally distributed spotted eagle ray (*Aetobatus narinari*). J Hered. 2009;100(3):273–83.
55. Last PR, Weigmann S, Naylor GJ. The Indo-Pacific stingray genus *Brevitrygon* (Myliobatiformes: Dasyatidae): Clarification of historical names and description of a new species, *B. manjajiae* sp. nov., from the Western Indian Ocean. Diversity. 12;15(12):1213.
56. Last PR, White WT, Naylor G. Three new stingrays (Myliobatiformes: Dasyatidae) from the Indo-West Pacific. Zootaxa. 2016c;4147(4):377–402.
57. Marouani S, Chaâba R, Kadri H, Saidi B, Bouain A, Maltagliati F, et al. Taxonomic research on *Squalus megalops* (Macleay, 1881) and *Squalus blainvillei* (Risso, 1827) (Chondrichthyes: Squalidae) in Tunisian waters (central Mediterranean Sea). Sci Mar. 2012;76(1):97–109.
58. Bonello JJ, Bonnici L, Ferrari A, Cariani A, Schembri PJ. Not all that clear cut: Intraspecific morphological variability in *Squalus blainville* (Risso, 1827) and implication for identification of this species. J Mar Biol Assoc UK. 2016;96(8):1585-1596.
59. Ferrari A, Di Crescenzo S, Cariani A, Crobe V, Benvenuto A, Piattoni F, et al. Puzzling over spurdogs: molecular taxonomy assessment of the *Squalus* species in the Strait of Sicily. Eur Zool J. 2021;88(1):181-90.
60. Akhilesh KV, White WT, Bineesh KK, Ganga U, Pillai NGK. Biological observations on the bristly catshark *Bythaelurus hispidus* from deep waters off the south-west coast of India. J Fish Biol. 2013; 82: 1582-1591.
61. Weigmann S, Kaschner CJ, Thiel R. A new microendemic species of the deep-water catshark genus Bythaelurus (Carcharhiniformes, Pentanchidae) from the northwestern Indian Ocean, with investigations of its feeding ecology, generic review and identification key. PLoS One. 2018;13(12):e0207887.
62. Ebert DA, Clerkin PJ. A new species of deep-sea catshark (Scyliorhinidae: *Bythaelurus*) from the southwestern Indian Ocean. JOSF. 2015;15:53-63.
63. Springer S. A revision of the catsharks, family Scyliorhinidae. NOAA Tech Rep NMFS. 1979;422: p. 1-152.
64. Boomer JJ, Harcourt RG, Francis MP, Stow AJ. Genetic divergence, speciation and biogeography of *Mustelus* (sharks) in the central Indo-Pacific and Australasia. Mol Phylogenet Evol. 2012;64(3):697–703.
65. Straube N, Iglésias SP, Sellos DY, Kriwet J, Schliewen UK. Molecular phylogeny and node time estimation of bioluminescent Lantern Sharks (Elasmobranchii: Etmopteridae). Mol Phylogenet Evol. 2010;56(3):905–17.
66. Last PR, Burgess GH, Séret B. Description of six new species of lantern-sharks of the genus *Etmopterus* (Squaloidea: Etmopteridae) from the Australasian region. Cybium. 2002;26:203-223.
67. Engel MS, Ceríaco LMP, Daniel GM, Dellapé PM, Löbl I, Marinov M, et al. The taxonomic impediment: a shortage of taxonomists, not the lack of technical approaches. Zool J Linn Soc. 2021;193(2):381–7.

68. Rheindt FE, Bouchard P, Pyle RL, Welter-Schultes F, Aescht E, Ahyong ST, et al. Tightening the requirements for species diagnoses would help integrate DNA-based descriptions in taxonomic practice. PLoS Biol. 2023;21(8):e3002251.
69. Morgan JAT, Harry AV, Welch DJ, Street R, White J, Geraghty PT, Macbeth WG, et al. Detection of interspecies hybridization in Chondrichthyes: hybrids and hybrid offspring between Australian (*Carcharhinus tilstoni*) and common (*C. limbatus*) blacktip shark found in an Australian fishery. Conserv Genet. 2012;13:455-463.
70. Cruz VP, Vera M, Mendoça FF, Pardo BG, Martinez P, Oliveira C, Foresti F. First identification of interspecies hybridization in the freshwater stingrays *Potamotrygon motoro* and *P. falkneri* (Myliobatiformes, Potamotrygonidae). Conserv Genet. 2015;16:241-245.
71. Marino IAM, Riginella E, Gristina M, Rasotto MB, Zane L, Mazzoldi C. Multiple paternity and hybridization in two smooth-hound sharks. Sci Rep. 2015;5:12919.
72. Corrigan S, Delser PM, Eddy C, Duffy C, Yang L, Li C, Bazinet AL, et al. Historical introgression drives pervasive mitochondrial admixture between two species of pelagic sharks. Mol Phylogenet Evol. 2017;110:122-126.
73. Baker AM, Adams DH, Briggers III WB, Frazier BS, Portnoy DS. Hybridization between sympatric hammerhead sharks in the western North Atlantic Ocean. Biol Lett. 2019;15:20190004. http://dx.doi.org/10.1098/rsbl.2019.0004.
74. Nevatte RJ, Williamson JE, Gillings MR. First evidence of multiple paternity and hybridization in Australian sawsharks. Mar Freshwater Res. 2023; 74(7):586-600.
75. Domingues RR, Bunholi IV, Pinhal D, Antuens A, Mendoça FF. From molecule to conservation: DNA-based methods to overcome frontiers in the shark and ray fin trade. Conserv Genet Resour. 2021;13:231-247.
76. Wainwright BJ, Ip YCA, Neo ML, Chang JJM, Gan CZ, Clark-Shen N, et al. DNA barcoding of traded shark fins, meat and mobulid gill plates in Singapore uncovers numerous threatened species. Conserv Genet. 2018;19(6):1393–9.
77. Hobbs CAD, Potts RWA, Bjerregaard Walsh M, Usher J, Griffiths AM. Using DNA barcoding to investigate patterns of species utilisation in UK shark products reveals threatened species on sale. Sci Rep. 2019;9(1):1028.
78. Choo MY, Choy CPP, Ip YCA, Rao M, Huang D. Diversity and origins of giant guitarfish and wedgefish products in Singapore. Aquat Conserv. 2021;31(7):1636–49.
79. Liu CJN, Neo S, Rengifo NM, French I, Chiang S, Ooi M, et al. Sharks in hot soup: DNA barcoding of shark species traded in Singapore. Fish Res.2021;241:105994.
80. Klangnurak W, Arunrugstichai S, Manopawitr P, Krajangdara. DNA-based species identification of shark fins traded in Thai markets. Conserv. Genet. 2023;24(4):537-546.

Fig 1A

Click here to access/download;Figure;fig1_A_sharks_COI.tif

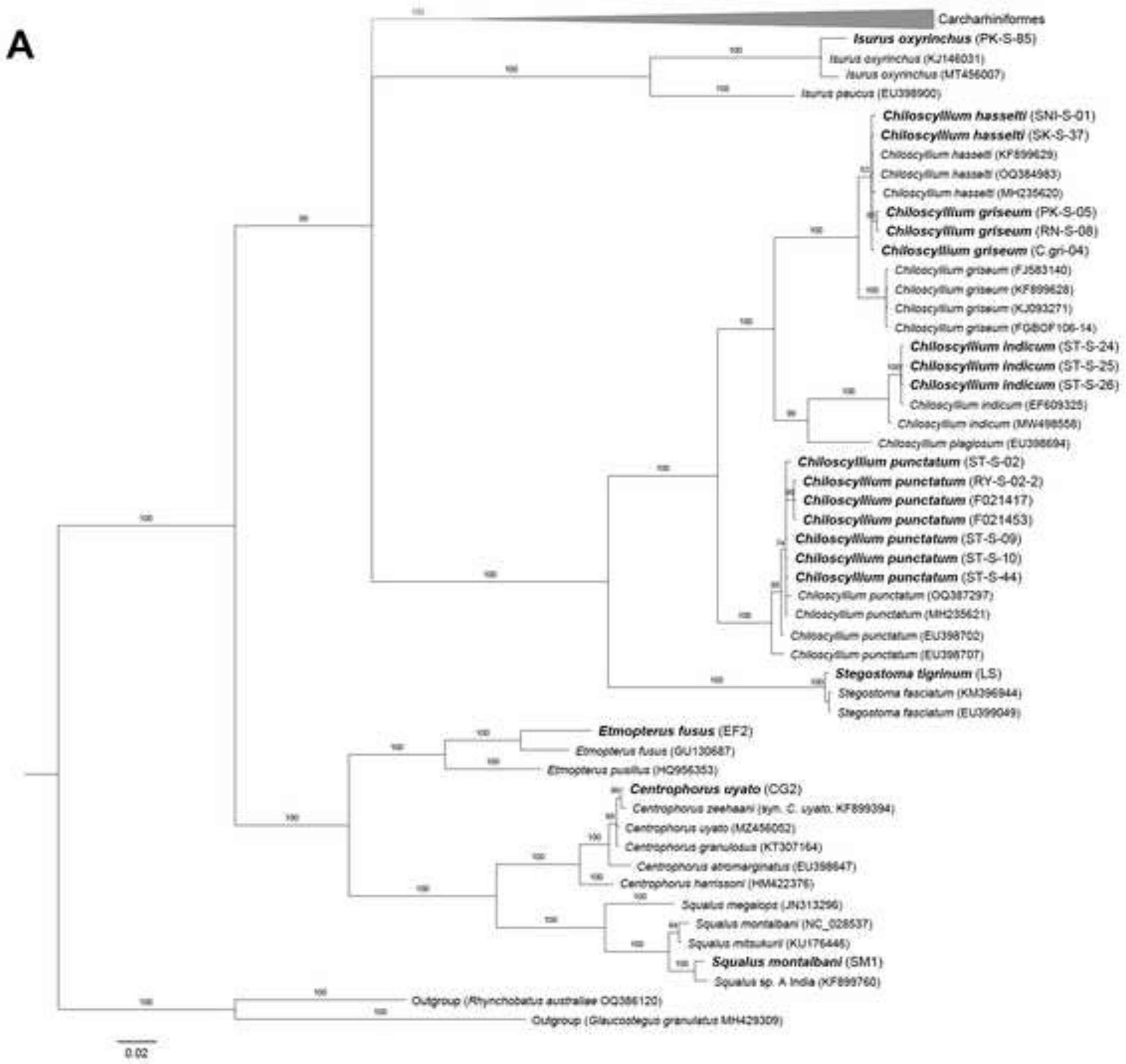

Fig 1B

Click here to access/download;Figure;fig1_B_sharks_COI.tif

B

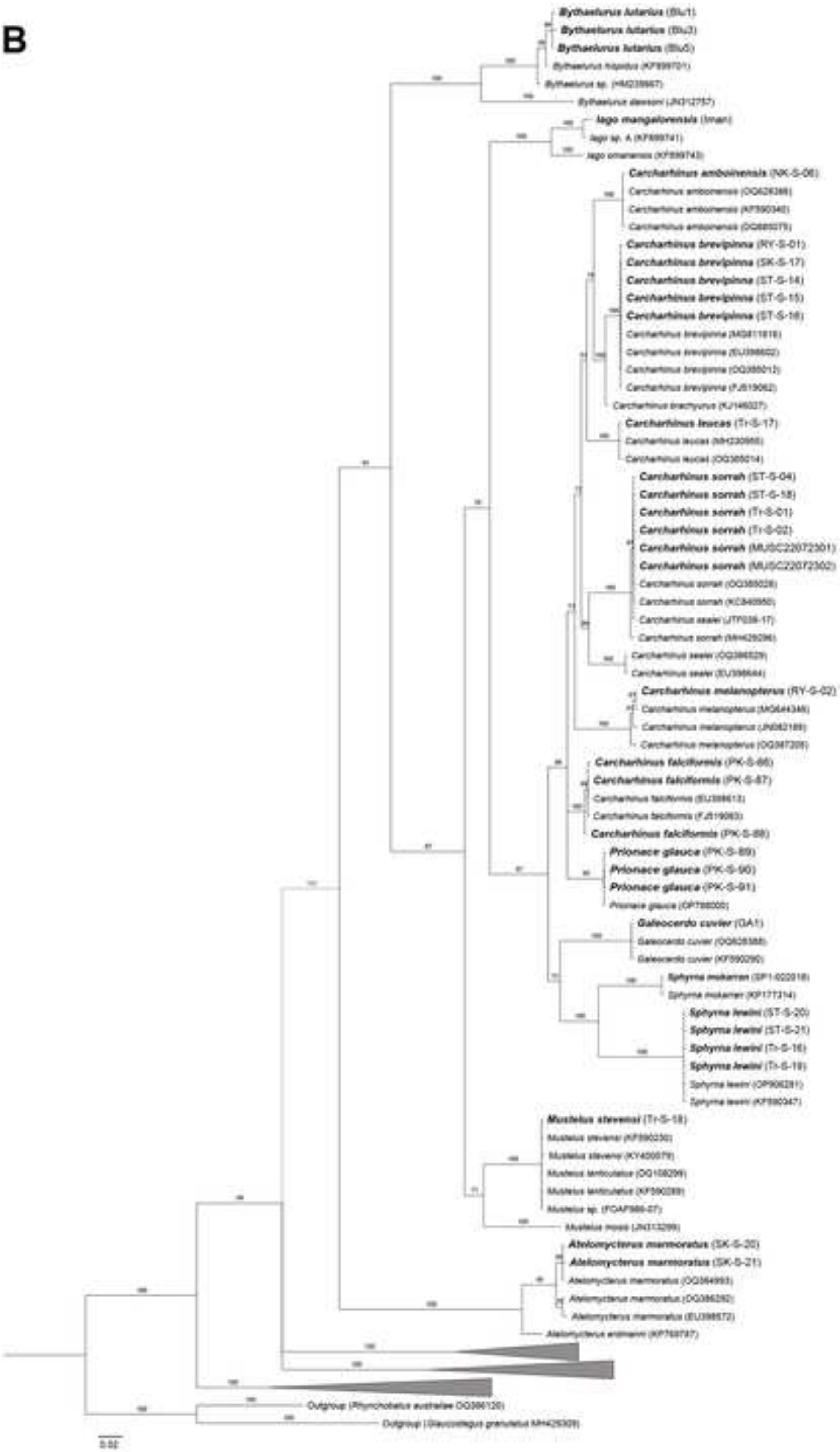

Fig 2A

Click here to access/download;Figure;fig2_A_sharks_ND2.tif

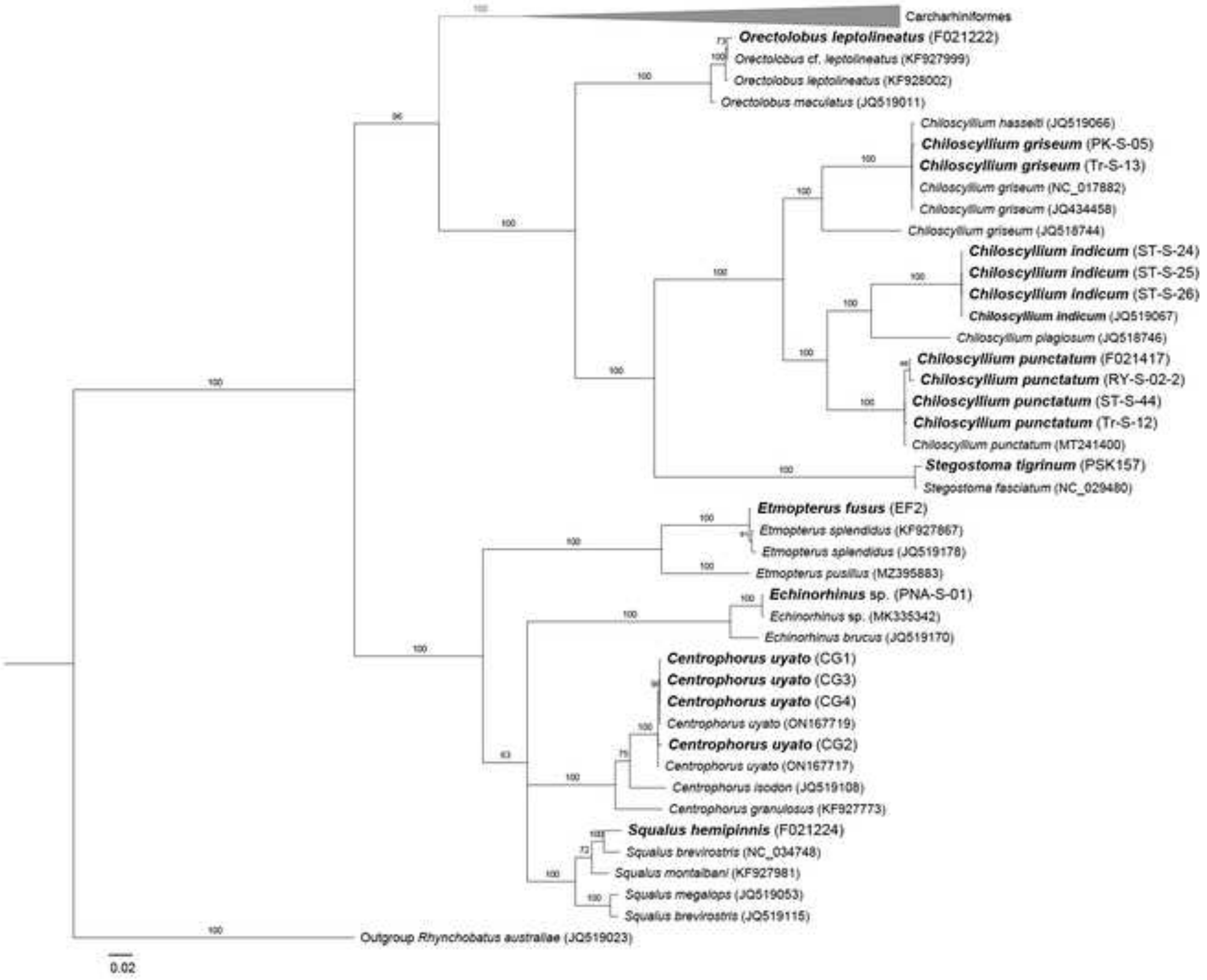

Fig 2B

Click here to access/download;Figure;fig2_B_sharks_ND2.tif

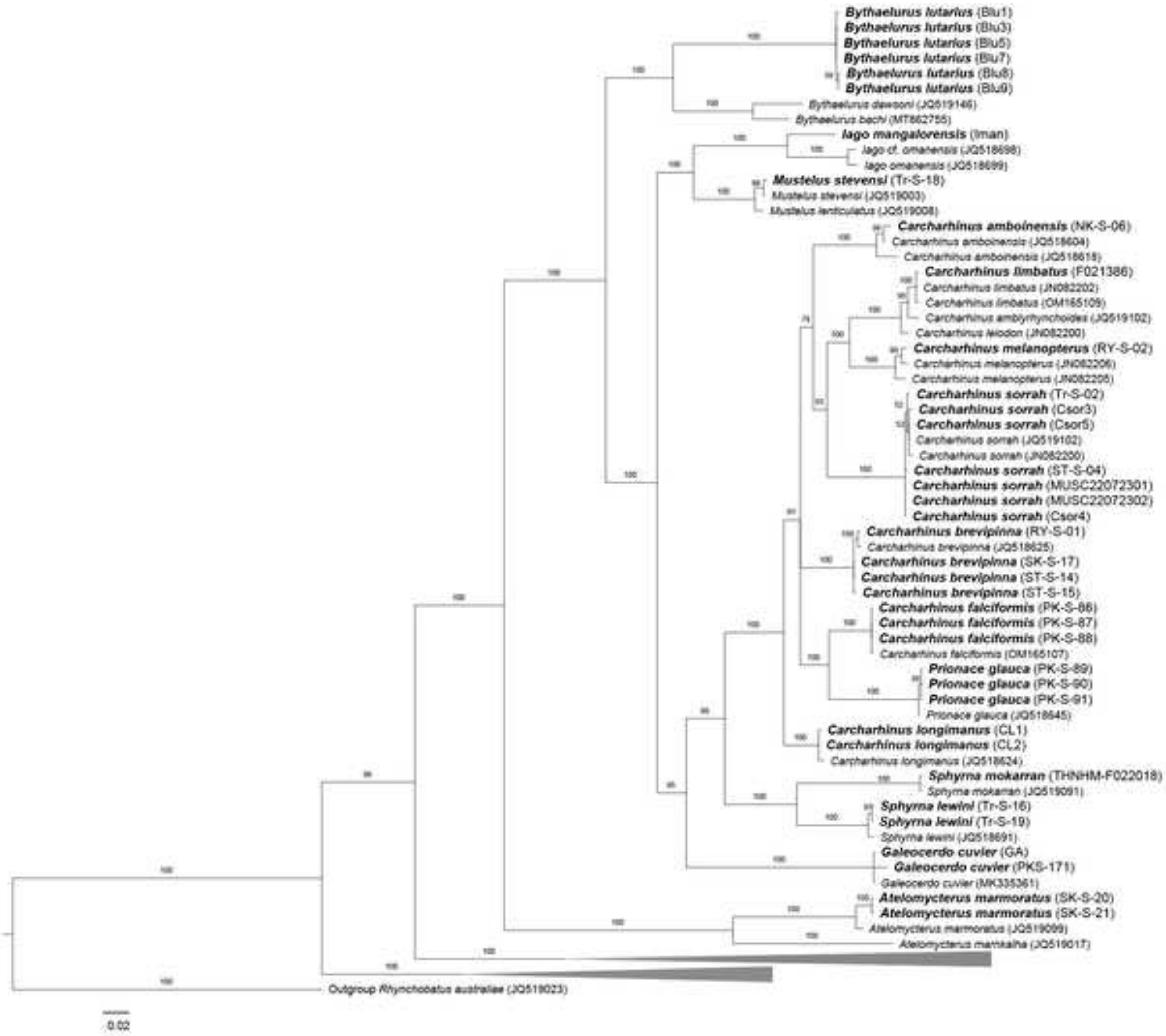

Fig 3A

Click here to access/download;Figure;fig3_A_Batoids_COI.tif

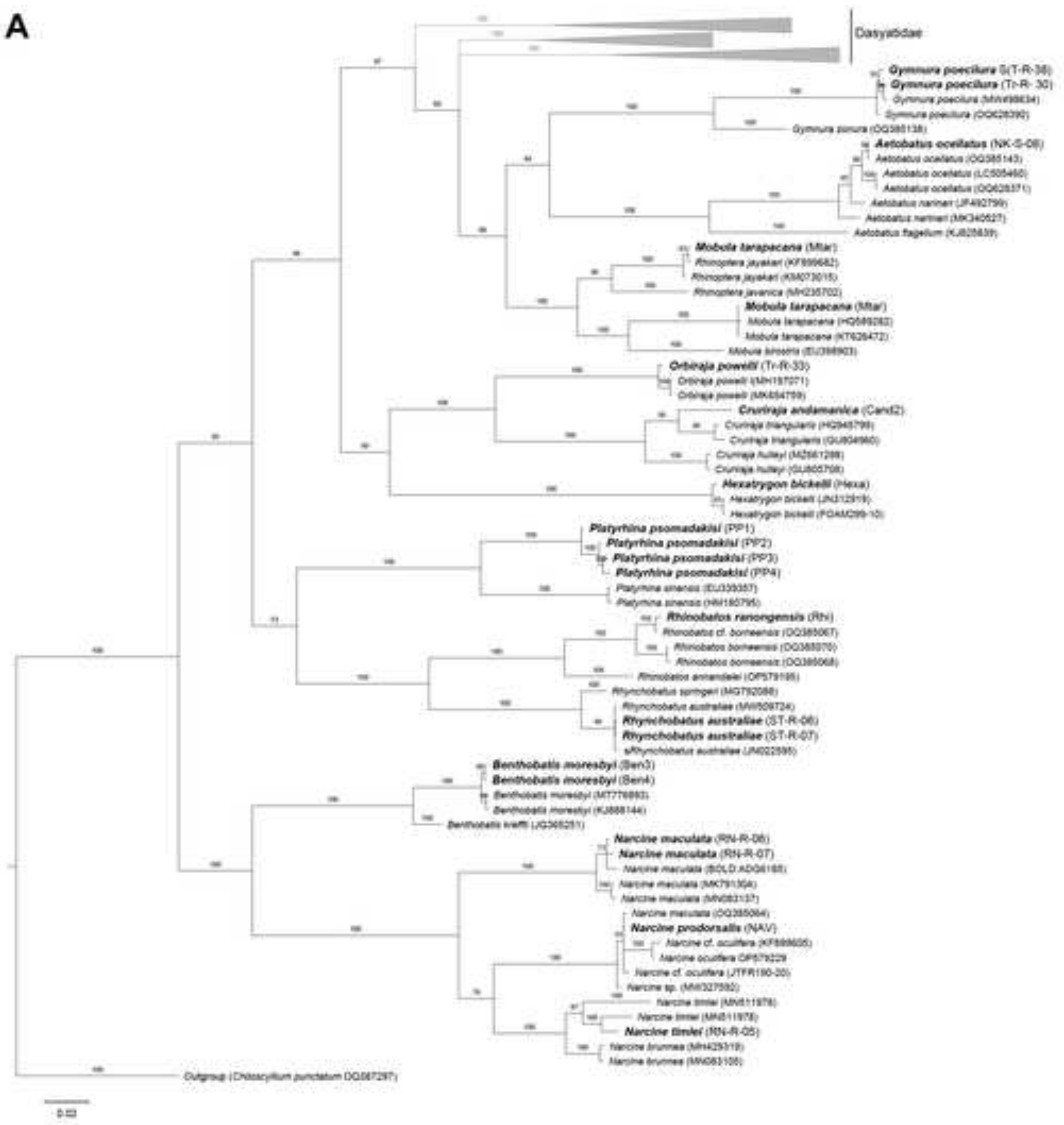

Fig 3B

Click here to access/download;Figure;fig3_B_Batoids_COI.tif

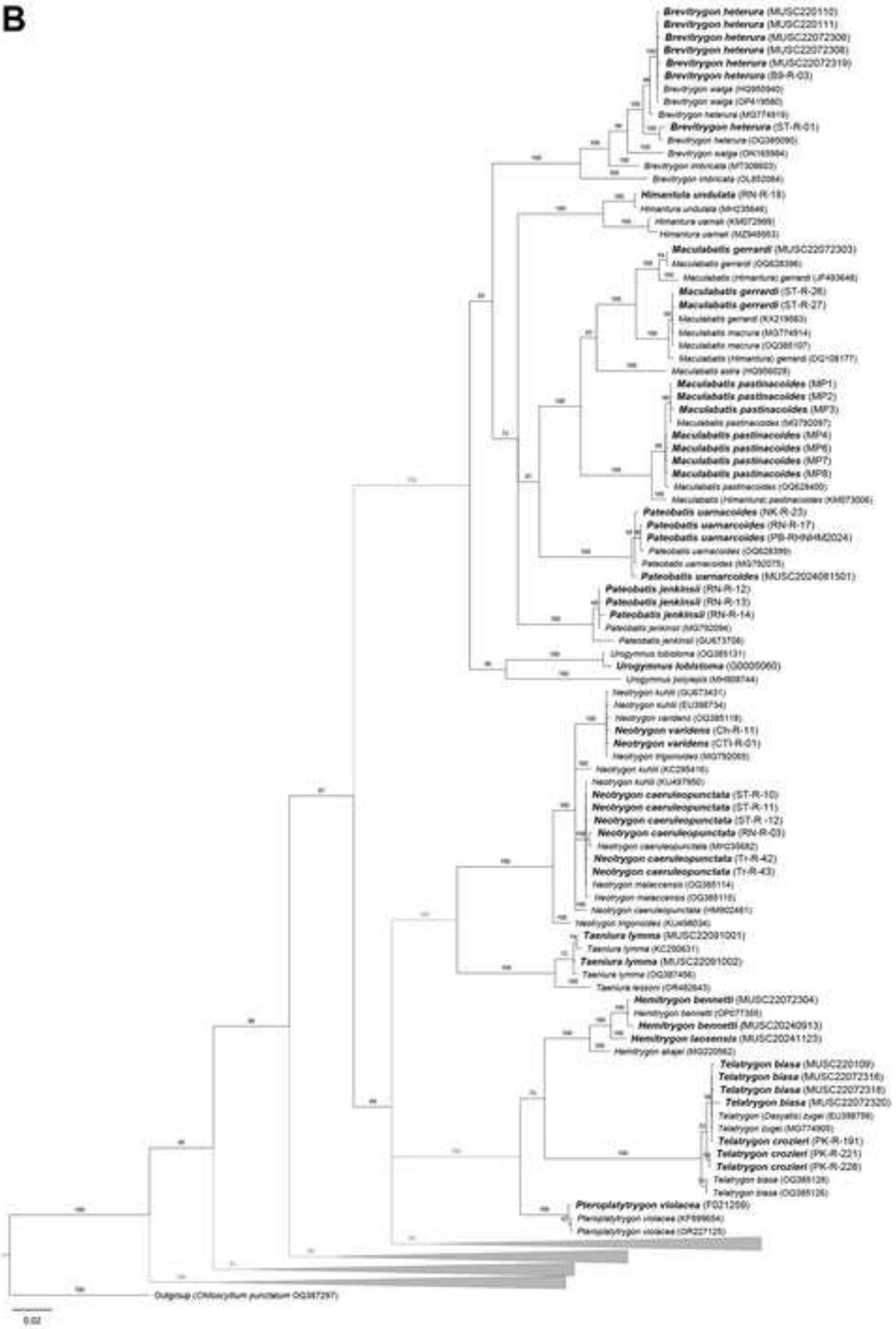

Fig 3C

Click here to access/download;Figure;fig3_C_batoid_COI_Glaucostegus.tif

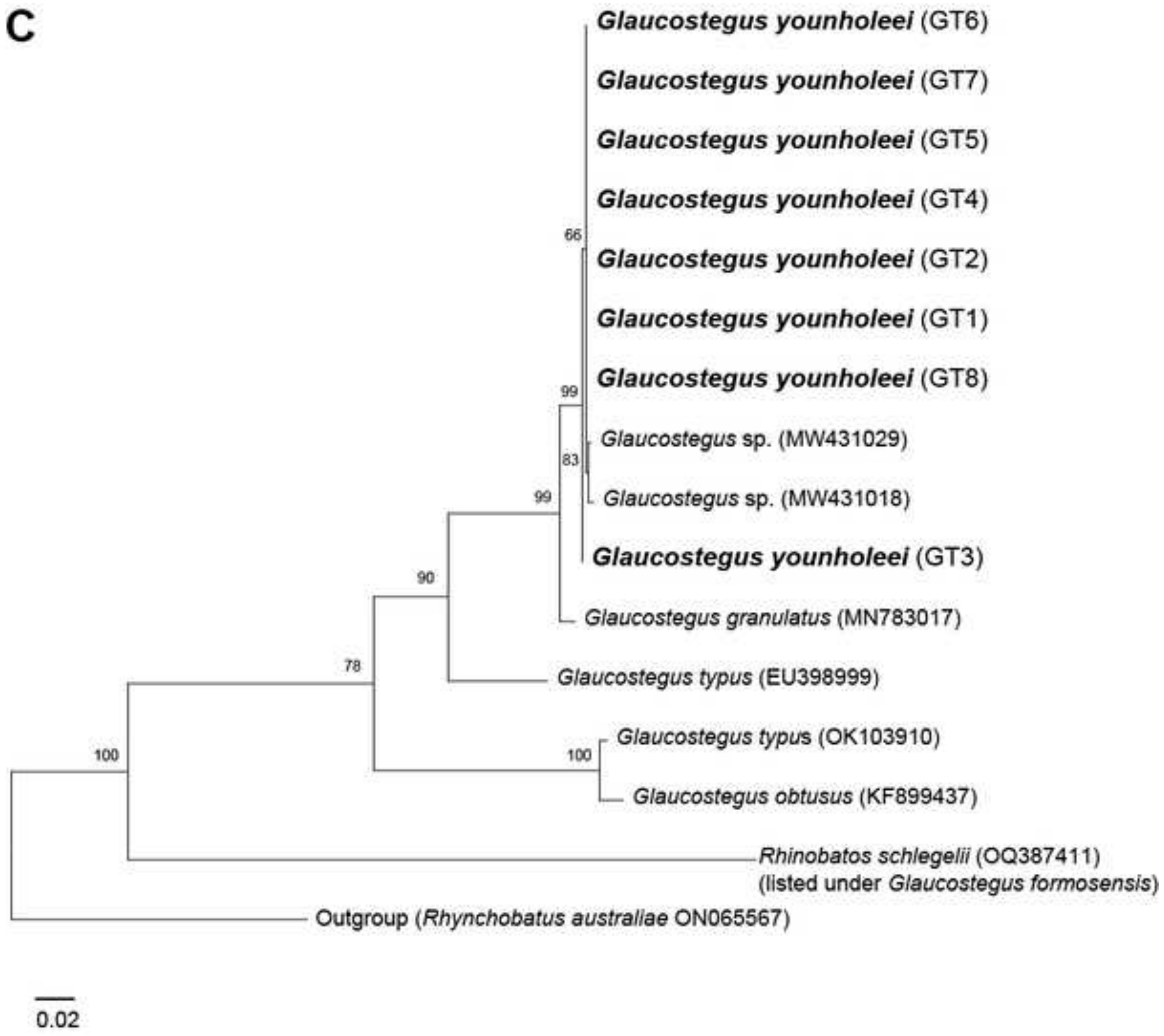

Fig 4A

Click here to access/download;Figure;fig4_A_Batoids_ND2.tif

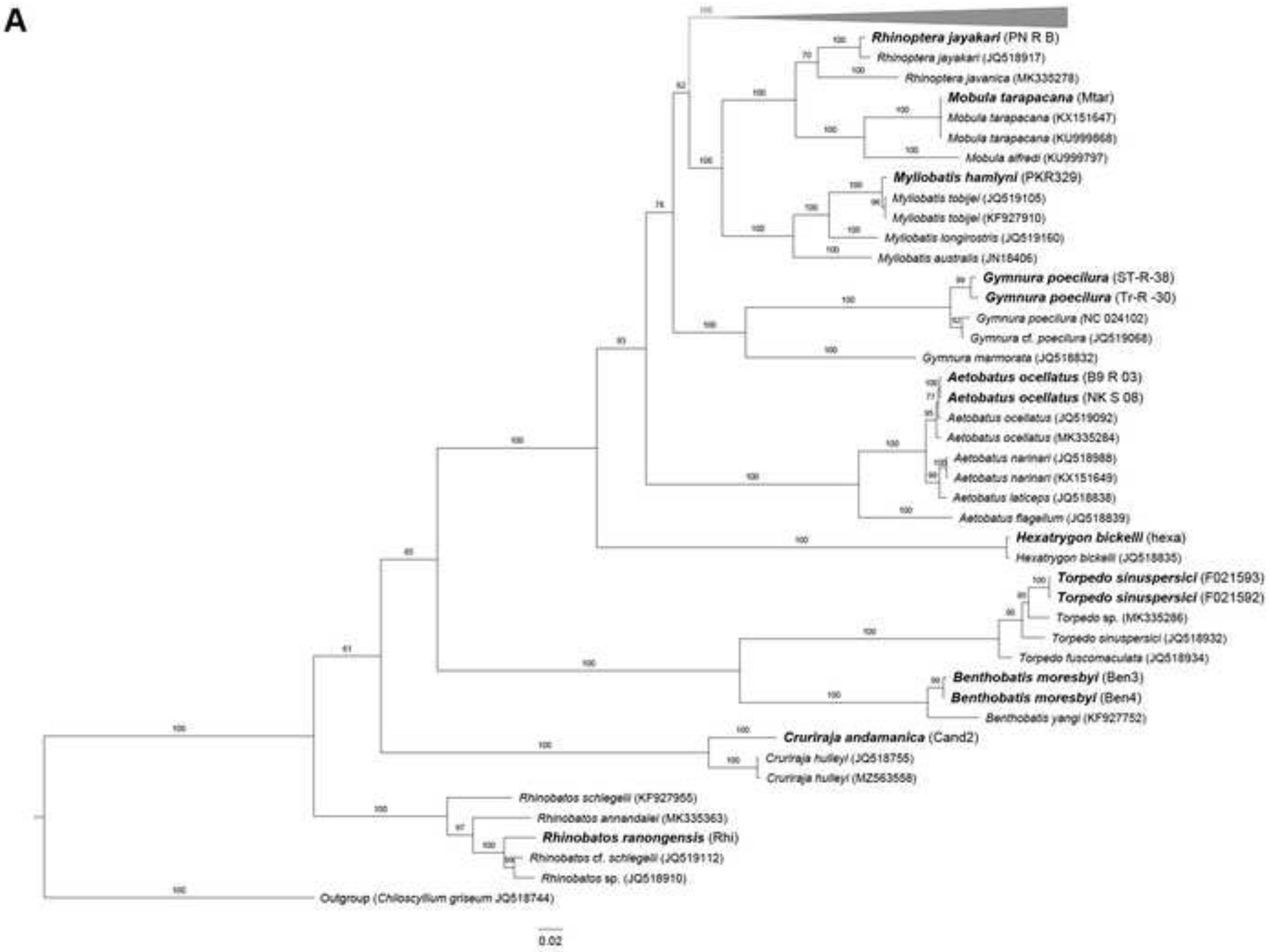

Fig 4B

Click here to access/download;Figure;fig4_B_Batoids_ND2.tif

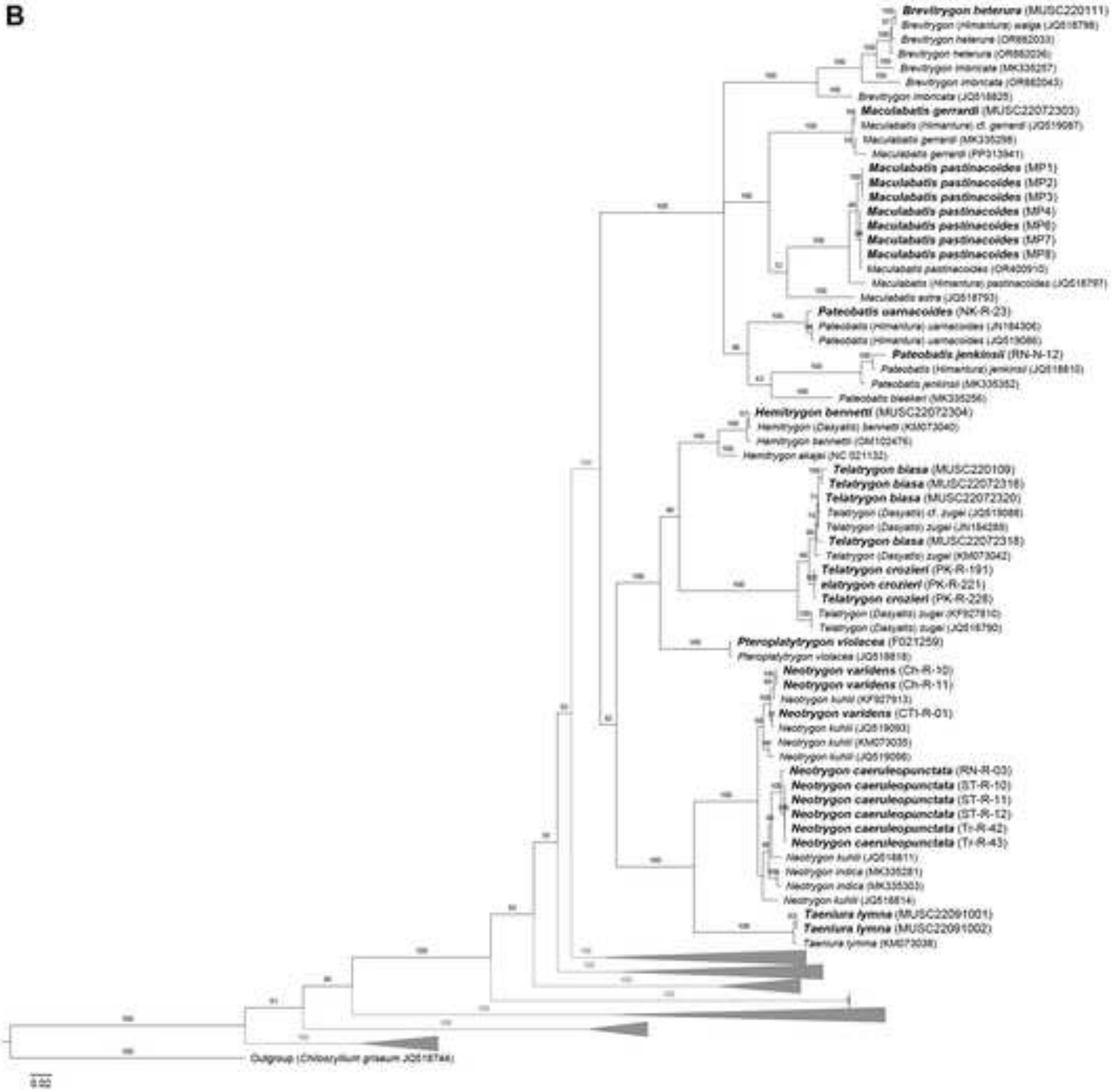

Fig 5

Click here to access/download;Figure;fig5_K2P_shark.tif

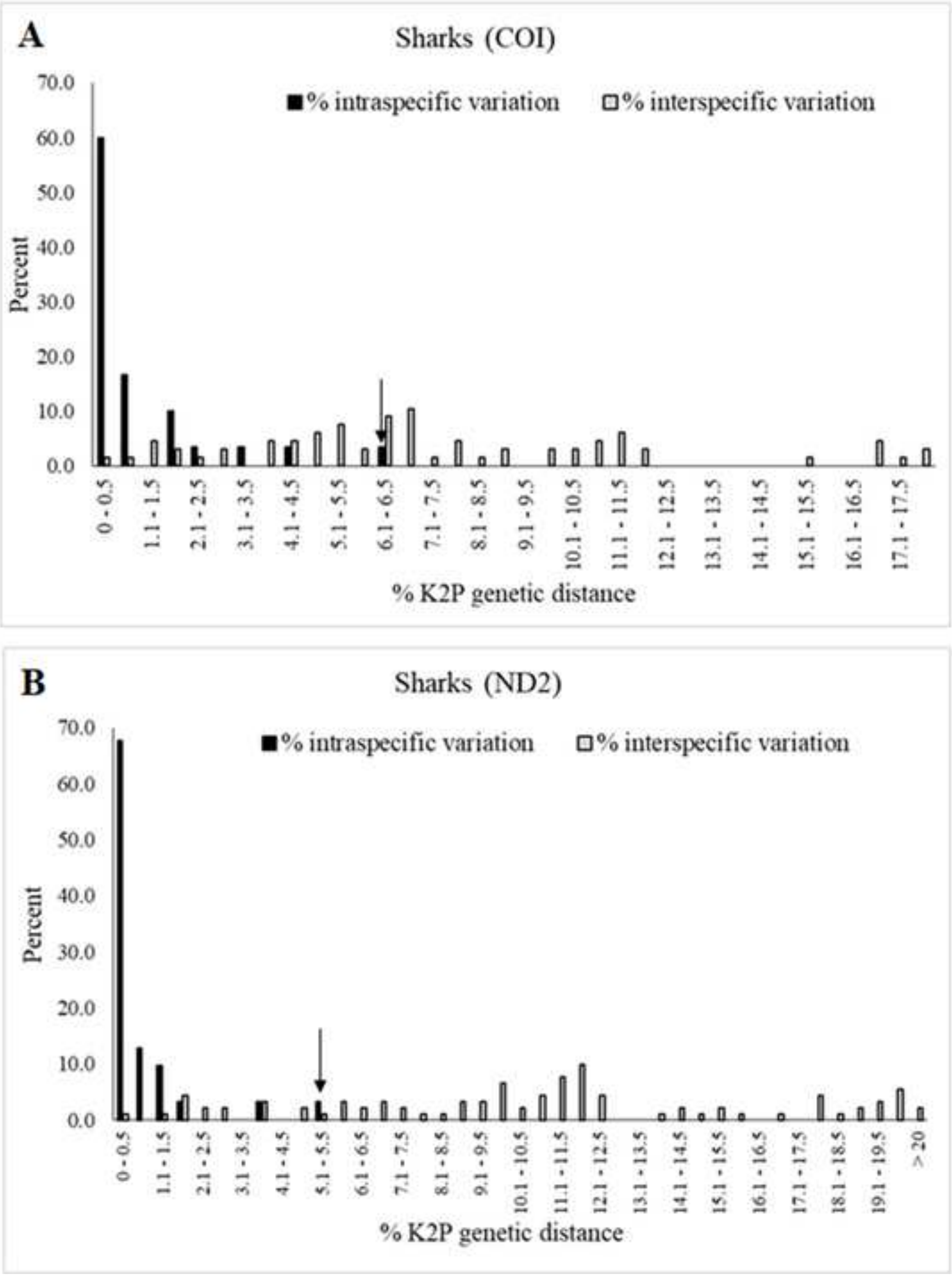

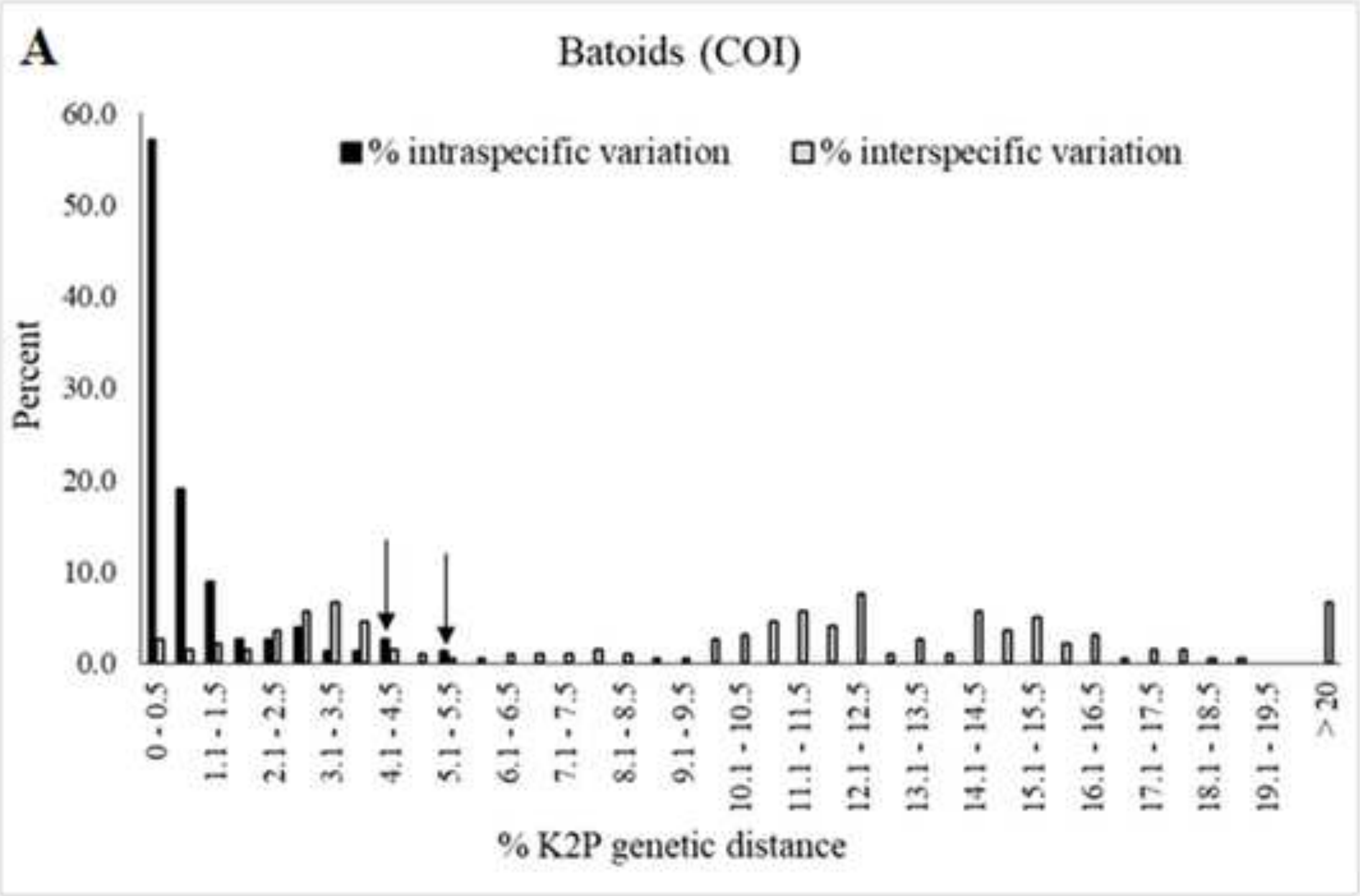
A
Batoids (COI)
% intraspecific variation
% interspecific variation
Percent
60.0
50.0
40.0
30.0
20.0
10.0
0.0
0 - 0.5
1.1 - 1.5
2.1 - 2.5
3.1 - 3.5
4.1 - 4.5
5.1 - 5.5
6.1 - 6.5
7.1 - 7.5
8.1 - 8.5
9.1 - 9.5
10.1 - 10.5
11.1 - 11.5
12.1 - 12.5
13.1 - 13.5
14.1 - 14.5
15.1 - 15.5
16.1 - 16.5
17.1 - 17.5
18.1 - 18.5
19.1 - 19.5
> 20
% K2P genetic distance

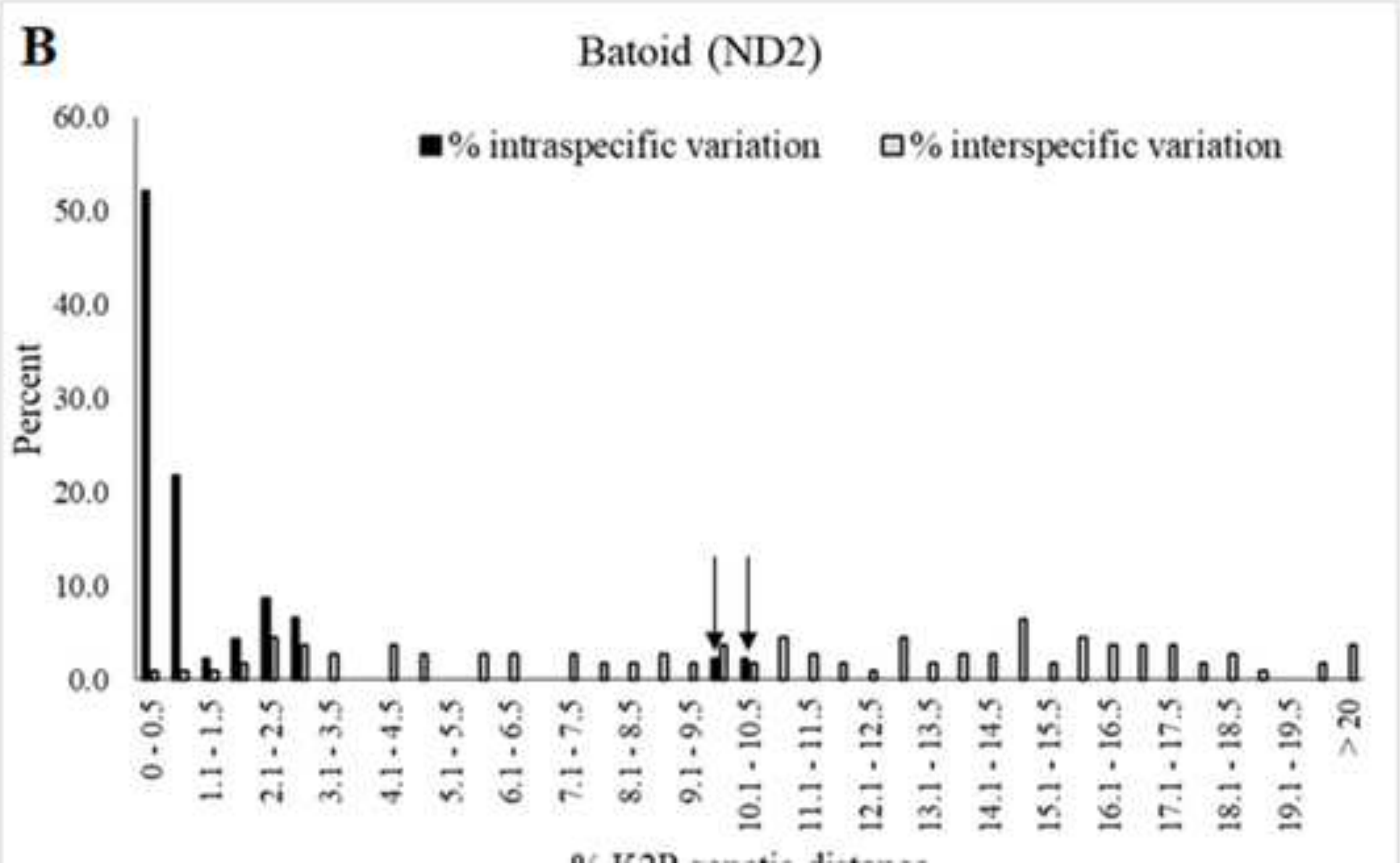
B
Batoid (ND2)
% intraspecific variation
% interspecific variation
Percent
60.0
50.0
40.0
30.0
20.0
10.0
0.0
0 - 0.5
1.1 - 1.5
2.1 - 2.5
3.1 - 3.5
4.1 - 4.5
5.1 - 5.5
6.1 - 6.5
7.1 - 7.5
8.1 - 8.5
9.1 - 9.5
10.1 - 10.5
11.1 - 11.5
12.1 - 12.5
13.1 - 13.5
14.1 - 14.5
15.1 - 15.5
16.1 - 16.5
17.1 - 17.5
18.1 - 18.5
19.1 - 19.5
> 20

Click here to access/download
Supporting Information
supporting_info_Khudamrongsawat_et_al.docx
