# Supplementary material for: DNA barcoding for elasmobranch diversity assessment in Thailand: Its advantages and limitations
Source: PLoS One. 2025 Oct 31;20(10):e0334640. doi: 10.1371/journal.pone.0334640 (PMC12578239; doi:10.1371/journal.pone.0334640)
Supplement: Attachment — Submitted filename: Review_Comments_Muhala.pdf [file pone.0334640.s005.pdf]

General Comments

The work presents an interesting structure and themes that are current and that help in understanding the classification of species.

The use of two mitochondrial genes for species delimitation can be a conjugation method to help in species discrimination, especially when it comes to cryptic species.

Comments

1. The work presents some spelling errors that the authors need to correct to allow for better understanding. For example, many sections on page 17 present a lot of redundancy and this is repeated in some other places. The lack of line numbering in the manuscript makes it difficult to provide better guidance, in this case I suggest that authors have access to the PDF to see the other comments.

2. Table 2 needs to be improved to allow clarity, although we are faced with a comparison between the two markers, I suggest that they be in different columns and that they include the voucher or codes from GENBANK or Bold System if they were submitted. Alias needs to make it clear that this data was submitted to these platforms.

3. In the methodologies chapter, there is a portion that must be reconstructed, it is results information.

4. You need to standardize the way you write species names.

5. Clarity is needed on some inconsistencies or impossibilities of the two markers to discriminate the species. It is important to make clear the methods used to validate the information.

6. Include a brief discussion regarding the high percentage of divergence within the species *Himantura leoparda and Narcine spp.*

7. Phylogenetic trees need to be reconstructed or better edited, they are not clear and the species evidenced in the study, especially the groups, need to have some signaling.

Considering all the aspects raised and after a review, the article can be considered for acceptance in this journal, so I recommend a minor review.
