# Supplementary material for: DNA barcoding for elasmobranch diversity assessment in Thailand: Its advantages and limitations
Source: PLoS One. 2025 Oct 31;20(10):e0334640. doi: 10.1371/journal.pone.0334640 (PMC12578239; doi:10.1371/journal.pone.0334640)
Supplement: Attachment — Submitted filename: comments of reviewer.pdf [file pone.0334640.s007.pdf]

The manuscript PONE-D-25-18018R1, entitled *"DNA barcoding for elasmobranch diversity assessment in Thailand: its advantages and limitations,"* is interesting; however, there are instances of redundancy, and some sentences could be rephrased for improved clarity and conciseness. Additionally, the absence of line numbers makes precise referencing challenging. Including line numbers would facilitate easier review and future revisions. Please ensure consistent formatting of species names (e.g., full genus names vs. abbreviations), particularly in the Results and Discussion sections.

Abstract

"mean for species identification" should be "means of species identification."
"exercise cautions" should be "exercise caution."

Introduction

"Available genetic information, though scant, poses many questions regarding taxonomic diversity in which the high levels of genetic differentiation that could not be ignored [11]": The phrasing "in which the high levels of genetic differentiation that could not be ignored" is a bit clunky. Consider rephrasing for smoother reading.

Methods

- Did the authors perform sequence analysis? Which software did the authors use? Mention it in the ms!

Results

- The authors' decision to keep Table 2 in the Results section as it illustrates and summarizes their findings is valid. The detailed information being in the supplementary section is also appropriate.
- The authors' preference for spelling out full generic names in the text to avoid confusion, especially given the similar initial letters of many elasmobranch genera, is a valid point. However, if the authors choose to abbreviate, ensure consistency (e.g., first use full name, then abbreviate).
- addition of morphometric ratios to differentiate *M. gerrardi* and *M. macrura* provides more robust identification criteria.
- "did not aligned": This should be "did not align."
