# Supplementary material for: DNA barcoding for elasmobranch diversity assessment in Thailand: Its advantages and limitations
Source: PLoS One. 2025 Oct 31;20(10):e0334640. doi: 10.1371/journal.pone.0334640 (PMC12578239; doi:10.1371/journal.pone.0334640)
Supplement: Attachment — Submitted filename: PONE-D-25-18018_R1_230725.pdf [file pone.0334640.s008.pdf]

PLOS ONE

DNA barcoding for elasmobranch diversity assessment in Thailand: its advantages and limitations

--Manuscript Draft--

**Manuscript Number:**	PONE-D-25-18018R1
**Article Type:**	Research Article
**Full Title:**	DNA barcoding for elasmobranch diversity assessment in Thailand: its advantages and limitations
**Short Title:**	DNA barcoding for elasmobranch diversity assessment in Thailand
**Corresponding Author:**	Wansuk Senanan, Ph.D.<br>Burapha University<br>Chonburi, Chonburi THAILAND
**Keywords:**	sharks, rays, batoid, COI, ND2, genetic variation, conservation
**Abstract:**	The assessment of elasmobranch biodiversity in Thailand benefits greatly from the application of DNA barcoding, which helps mitigate the challenge posed by a shortage of expert taxonomists. Fragments of COI and ND2 mitochondrial DNA were examined, and the strength and weaknesses of these two markers were compared. In this study, DNA products from 153 elasmobranch samples were amplifiable and revealed a total of 28 shark species and 32 batoid species. Many species could be confidently identified as their morphological characteristics aligned with DNA barcodes. However, several exceptions were recognized. The absence of reference sequences for rare species presented a challenge for species verification, and the misidentification of reference sequences, as well as changes in species names due to taxonomic revisions, added complexity when comparing DNA barcoding sequences. Conflicts between morphology and genetics were also observed. While intraspecific genetic variation based on both DNA barcodes generally indicated 0-2% variation, this metric could not always be used for species delimitation. This was particularly true for species displaying low genetic variation among closely related species and species where cryptic diversity remained hidden and yet to be uncovered. In such cases, the morphological characteristics of the samples served as a primary means of species identification. Despite these challenges, DNA barcoding remains an invaluable tool for biodiversity assessment, especially in light of the shortage of skilled experts and identification of products made of vulnerable species. However, it is essential to exercise caution and be aware of these complexities in its application.
**Order of Authors:**	Jenjit Khudamrongsawat, Ph.D.
	Tassapon Krajangdara
	Thadsin Panithanarak, Ph.D.
	Ratima Karuwancharoen
	Wanlada Klangnurak, Ph.D.
	Pattarapon Promnun
	Wansuk Senanan, Ph.D.
**Response to Reviewers:**	We make the corrections in response to reviewers' suggestions and questions (see attached response to reviewers for detailed revisions). In addition, we made some small adjustment to the content:<br>•Adding the section “Financial disclosure”<br>•Move the ethic statements (previously in the Acknowledgements) to method section<br>•Separate supporting information into 2 files: S1—primers and protocol used, S2—sample identification including morphological and genetic examination of each sample.
**Additional Information:**	
**Question**	**Response**

Powered by Editorial Manager® and ProduXion Manager® from Aries Systems Corporation

**Financial Disclosure**<br><br>Enter a financial disclosure statement that describes the sources of funding for the work included in this submission. Review the submission guidelines for detailed requirements. View published research articles from *PLOS ONE* for specific examples.<br><br>This statement is required for submission and **will appear in the published article** if the submission is accepted. Please make sure it is accurate.<br><br>**Funded studies**<br>Enter a statement with the following details:<br>• Initials of the authors who received each award<br>• Grant numbers awarded to each author<br>• The full name of each funder<br>• URL of each funder website<br>• Did the sponsors or funders play any role in the study design, data collection and analysis, decision to publish, or preparation of the manuscript?<br><br>Did you receive funding for this work?	Yes
Please add funding details.<br>as follow-up to "**Financial Disclosure**<br><br>Enter a financial disclosure statement that describes the sources of funding for the work included in this submission. Review the submission guidelines for detailed requirements. View published research articles from *PLOS ONE* for specific examples.<br><br>This statement is required for submission and **will appear in the published article** if the submission is accepted. Please make sure it is accurate.	This project is funded by National Research Council of Thailand (NRCT-N25A650485). The funders had no role in study design, data collection and analysis, decision to publish, or preparation of the manuscript.

Powered by Editorial Manager® and ProduXion Manager® from Aries Systems Corporation

**Funded studies**<br>Enter a statement with the following details:<br>• Initials of the authors who received each award<br>• Grant numbers awarded to each author<br>• The full name of each funder<br>• URL of each funder website<br>• Did the sponsors or funders play any role in the study design, data collection and analysis, decision to publish, or preparation of the manuscript?<br><br>Did you receive funding for this work?"	
Please select the country of your main research funder (please select carefully as in some cases this is used in fee calculation).<br>as follow-up to "**Financial Disclosure**<br><br>Enter a financial disclosure statement that describes the sources of funding for the work included in this submission. Review the submission guidelines for detailed requirements. View published research articles from *PLOS ONE* for specific examples.<br><br>This statement is required for submission and **will appear in the published article** if the submission is accepted. Please make sure it is accurate.<br><br>**Funded studies**<br>Enter a statement with the following details:<br>• Initials of the authors who received each award<br>• Grant numbers awarded to each author<br>• The full name of each funder<br>• URL of each funder website<br>• Did the sponsors or funders play any role in the study design, data collection and analysis, decision to publish, or preparation of the manuscript?<br><br>Did you receive funding for this work?"	THAILAND - TH
**Competing Interests**	The authors have declared that no competing interests exist.

Use the instructions below to enter a competing interest statement for this submission. On behalf of all authors, disclose any competing interests that could be perceived to bias this work—acknowledging all financial support and any other relevant financial or non-financial competing interests.<br><br>This statement is required for submission and **will appear in the published article** if the submission is accepted. Please make sure it is accurate and that any funding sources listed in your Funding Information later in the submission form are also declared in your Financial Disclosure statement.<br><br>View published research articles from *PLOS ONE* for specific examples.<br><br>**NO authors have competing interests**<br><br>Enter: *The authors have declared that no competing interests exist.*<br><br>**Authors with competing interests**<br><br>Enter competing interest details beginning with this statement:<br><br>*I have read the journal's policy and the authors of this manuscript have the following competing interests: [insert competing interests here]*<br><br>* typeset	
**Ethics Statement**<br><br>Enter an ethics statement for this submission. This statement is required if the study involved:<br><br>• Human participants<br>• Human specimens or tissue<br>• Vertebrate animals or cephalopods<br>• Vertebrate embryos or tissues	Permission for animal use was granted by MU-IACUC (permission MUSC62-025-489) and Burapha University (IACUC 002/2566).

Powered by Editorial Manager® and ProduXion Manager® from Aries Systems Corporation

• Field research<br><br>Write "N/A" if the submission does not require an ethics statement.<br><br>General guidance is provided below. Consult the submission guidelines for detailed instructions. **Make sure that all information entered here is included in the Methods section of the manuscript.**	

Powered by Editorial Manager® and ProduXion Manager® from Aries Systems Corporation

**Format for specific study types**<br><br>**Human Subject Research (involving human participants and/or tissue)**<br>• Give the name of the institutional review board or ethics committee that approved the study<br>• Include the approval number and/or a statement indicating approval of this research<br>• Indicate the form of consent obtained (written/oral) or the reason that consent was not obtained (e.g. the data were analyzed anonymously)<br><br>**Animal Research (involving vertebrate animals, embryos or tissues)**<br>• Provide the name of the Institutional Animal Care and Use Committee (IACUC) or other relevant ethics board that reviewed the study protocol, and indicate whether they approved this research or granted a formal waiver of ethical approval<br>• Include an approval number if one was obtained<br>• If the study involved *non-human primates*, add *additional details* about animal welfare and steps taken to ameliorate suffering<br>• If anesthesia, euthanasia, or any kind of animal sacrifice is part of the study, include briefly which substances and/or methods were applied<br><br>**Field Research**<br><br>Include the following details if this study involves the collection of plant, animal, or other materials from a natural setting:<br>• Field permit number<br>• Name of the institution or relevant body that granted permission	
**Data Availability**<br><br>Authors are required to make all data underlying the findings described fully available, without restriction, and from the time of publication. PLOS allows rare exceptions to address legal and ethical concerns. See the PLOS Data Policy and FAQ for detailed information.	Yes - all data are fully available without restriction

A Data Availability Statement describing where the data can be found is required at submission. Your answers to this question constitute the Data Availability Statement and **will be published in the article**, if accepted.<br><br>**Important:** Stating 'data available on request from the author' is not sufficient. If your data are only available upon request, select 'No' for the first question and explain your exceptional situation in the text box.<br><br>Do the authors confirm that all data underlying the findings described in their manuscript are fully available without restriction?	
**Describe where the data may be found in full sentences. If you are copying our sample text, replace any instances of XXX with the appropriate details.**<br><br>• If the data are **held or will be held in a public repository**, include URLs, accession numbers or DOIs. If this information will only be available after acceptance, indicate this by ticking the box below. For example: *All XXX files are available from the XXX database (accession number(s) XXX, XXX.*).<br>• If the data are all contained **within the manuscript and/or Supporting Information files**, enter the following: *All relevant data are within the manuscript and its Supporting Information files.*<br>• If neither of these applies but you are able to provide **details of access elsewhere**, with or without limitations, please do so. For example:<br><br>*Data cannot be shared publicly because of [XXX]. Data are available from the XXX Institutional Data Access / Ethics Committee (contact via XXX) for researchers who meet the criteria for access to confidential data.*<br><br>*The data underlying the results presented in the study are available from (include the name of the third party*	We included all the Genbank accession numbers for the nucleotide sequences in the Supporting Information file. These accession number will be released upon publication being published (https://www.ncbi.nlm.nih.gov/).

Powered by Editorial Manager® and ProduXion Manager® from Aries Systems Corporation

and contact information or URL).<br>• This text is appropriate if the data are owned by a third party and authors do not have permission to share the data.<br><br>* typeset	
Additional data availability information:	Tick here if the URLs/accession numbers/DOIs will be available only after acceptance of the manuscript for publication so that we can ensure their inclusion before publication.

Manuscript

Click here to access/download;Manuscript;text_1_2025-03-21_revision1w_cle

DNA barcoding for elasmobranch diversity assessment in Thailand: its advantages and limitations

Jenjit Khudamrongsawat[1], Tassapon Krajangdara[2], Thadsin Panithanarak[3], Ratima Karuwancharoen[3], Wanlada Klangnurak[4], Pattarapon Promnun[5], Wansuk Senanan[6**]

[1]Department of Biology, Faculty of Science, Mahidol University, Rama VI Road, Rajathewi District, Bangkok, 10400, Thailand
[2]66/27 Soi 5, Thep Thani Village, Moo 2, Rasada Subdistrict, Muang District, Phuket Province 83000, Thailand
[3]Institute of Marine Science, Burapha University, 169 Long Had Bangsaen Rd, Saen Suk, Chon Buri District, Chon Buri 20131, Thailand
[4]Department of Animal Production Technology and Fishery, School of Agricultural Technology, King Mongkut's Institute of Technology Ladkrabang, Bangkok, 10520, Thailand
[5]Geneus Genetics Co.,Ltd, 37/1 Sukhumvit 101/1, Bang Chak, Phra Kanong, Bangkok, 10260, Thailand
[6]Department of Aquatic Science, Burapha University, 169 Long Had Bangsaen Rd, Saen Suk, Chon Buri District, Chon Buri 20131, Thailand

******corresponding author

Abstract

The assessment of elasmobranch biodiversity in Thailand benefits greatly from the application of DNA barcoding, which helps mitigate the challenge posed by a shortage of expert taxonomists. Fragments of COI and ND2 mitochondrial DNA were examined, and the strength and weaknesses of these two markers were compared. In this study, DNA products from 153 elasmobranch samples were amplifiable and revealed a total of 28 shark species and 32 batoid species. Many species could be confidently identified as their morphological characteristics aligned with DNA barcodes. However, several exceptions were recognized. The absence of reference sequences for rare species presented a challenge for species verification, and the misidentification of reference sequences, as well as changes in species names due to taxonomic revisions, added complexity when comparing DNA barcoding sequences. Conflicts between morphology and genetics were also observed. While intraspecific genetic variation based on both DNA barcodes generally indicated 0-2% variation, this metric could not always be used for species delimitation. This was particularly true for species displaying low genetic variation among closely related species and species where cryptic diversity remained hidden and yet to be uncovered. In such cases, the morphological characteristics of the samples served as a primary means of species identification. Despite these challenges, DNA barcoding remains an invaluable tool for biodiversity assessment, especially in light of the shortage of skilled experts and identification of products made of vulnerable species. However, it is essential to exercise caution and be aware of these complexities in its application.

Keywords: sharks, rays, batoid, COI, ND2, genetic variation, conservation

Introduction

The Southeast Asian region (SEA) boasts a remarkable array of elasmobranch species in which the ongoing discovery of new, undescribed species is adding to our appreciation of the extraordinary diversity of this under-studied group [1, 2]. To safeguard these taxa in the region, the Southeast Asian Fisheries Development Center (SEAFDEC) has been actively encouraging SEA countries to assess their elasmobranch diversity so as to better formulate international conservation policies. Among SEA nations, Thailand is one of chondrichthyan biodiversity hotspots. This is attributable to the country's expansive marine ecosystem covering nearly 3,000 kilometers of coastlines, spanning both the Indo-West Pacific region and the Indian Ocean. The geological dynamics and intricate evolutionary history further contribute to this biodiversity wealth [3, 4]. The latest records unveil over 180 species (across 12 orders and 43 families), including some apparently distinct, yet morphologically similar lineages are yet to be confirmed as full species [5]. Evident disparities exist in elasmobranch diversity between the Gulf of Thailand and the Andaman Sea. The species composition within the Gulf of Thailand tends to closely resemble that of the western Pacific region, whereas the Andaman Sea shows resemblances to species prevalent in the east Indian Ocean. Notably, the Andaman Sea boasts a higher number of elasmobranch species compared to the Gulf of Thailand. This discrepancy is further underscored by the presence of Holocephali species exclusively recorded in the Andaman Sea [5, 6]. The escalating species count is a result of enhanced survey efforts and the integration of molecular tools for species identification. Despite ongoing surveys and the submission of DNA barcode information to the NCBI database and Barcode of Life Database (BOLD), Thailand's remarkable elasmobranch diversity remains poorly in the existing literature [7–10]. Available genetic information, though scant, poses many questions regarding taxonomic diversity in which the high levels of genetic differentiation that could not be ignored [11].

The evaluation of elasmobranch diversity hinges on the utilization of both morphological and molecular tools, with DNA barcoding especially the cytochrome c oxidase subunit I (COI) and the nicotinamide adenine dehydrogenase 2 (ND2) gene fragments, gaining prominence [7, 12–15]. DNA barcoding facilitates the differentiation of cryptic species that are difficult to distinguish based on appearance alone [16]. Even species with extensive and apparently continuous distributions may exhibit genetic variations that have implications for disparate evolutionary trajectories [17, 18]. Specifically, ND2 has shown superiority in uncovering genetic variations in cryptic sharks and is strongly recommended for use in chondrichthyan DNA barcoding [7, 19]. While DNA barcoding offers a powerful and expeditious tool for assessing biodiversity, it has proven challenging to establish clear criteria for genetic differentiation between species, particularly for non-taxonomists. In many instances, genetic variations within elasmobranchs fall within a 2% range at the intraspecific level [13, 15, 20–22]. However, exceptions do exist [13, 15, 19, 23, 24]. These strengths and weaknesses of DNA barcoding for biodiversity assessment in various taxa have also been acknowledged [25–33]. Nonetheless, as Thailand has an urgent need for improved the country's biodiversity assessment, the adoption of

DNA barcoding is a worthwhile endeavor. Genetic insights into species have yielded significant advances in shaping the Thailand National Plan of Action for the Conservation and Management of Sharks (NPOA-Sharks, Thailand). This aligns with the nation's economic and development agenda in accordance with the United Nations' 2030 Sustainable Development Goals (SDGs).

This research's objectives were to evaluate the extent of DNA barcoding application based on COI and ND2 fragments for both taxonomists and non-taxonomists in elasmobranch biodiversity assessment and to create DNA barcoding databases of elasmobranch diversity in Thailand. The outcomes hold the potential to provide valuable contributions to the regional conservation efforts by shedding light on Thailand's unique biological resources.

Methods

- **Tissue sampling locations and collections**

Permission for animal use was granted by MU-IACUC (permission MUSC62-025-489) and Burapha U-ethics (IACUC 002/2566). Samples were obtained from various sources, encompassing research vessels, fish markets, and fishing harbors situated in both the Gulf of Thailand and the Andaman Sea over the period from 2013 to 2023. These specimens were photographed and underwent subsequent species identification by T. Krajangdara following the guidance provided in Krajangdara et al. [5], Last et al. [34], Krajangdara [35], and Ebert et al. [36]. Instances where characteristics proved intricate or displayed unclear attributes were categorized as unidentified species, designated with "cf." or "sp." To definitively classify these cases, molecular analysis was performed. Muscle or fin clips were excised from the specimens and conserved in 95% ethanol, subsequently being frozen at -20°C at Burapha University, Mahidol University, King Mongkut's Institute of Technology Ladkrabang, and the Natural History Museum of Thailand. In cases involving sizeable, expensive, or live individuals, photographs were taken as records, with the focus being solely on acquiring tissue samples. To ensure the future viability of the research, selected samples were designated as voucher specimens and retained at these institutions: the Phuket Marine Biological Center, Burapha University, King Mongkut's Institute of Technology Ladkrabang, and Natural History Museum of Thailand.

- **Molecular examination**

DNA samples were extracted from more than 200 samples using commercial Extraction Kit (QIAGEN─DNeasy kit). The quality of the extracted DNA samples was assessed by electrophoresis on a 1% agarose gel. Their concentrations were measured using a Nanodrop (NanoVue^{TM}). DNA barcoding based on cytochrome c oxidase subunit I (COI) and NADH dehydrogenase subunit 2 (ND2) were amplified following the protocols by Ward et al. [12] and Naylor et al. [7]. Degraded tissue samples, especially those obtained before 2020, could not be amplified using universal primers, and so new primers were designed for investigation. List of primers used in this study were listed in Supporting information (S1 File). Polymerase chain reactions (PCR) were carried out with a reaction total volume of 25 ul using 5-7 ng of DNA template, 2 µl (0.2 mM) dNTP, 2.5 µl 10xPCR buffer, 1 µl (0.5 µM) for each primer, 1 µl (2 mM) $MgCl_2$, 0.05 µl (5 unit/µl) Taq DNA polymerase, and deionized water. PCR cycles were performed with an initial denaturation at 94°C for 3 min, followed by 35 cycles of 94°C for 50 s,

annealing at 50°C for 2 min, and extension at 72°C for 90 s, and a final extension at 72°C for 6 min. For samples posing difficulty in amplification, a touchdown PCR profile was performed including an initial denaturation at 94°C for 3 min, followed by 5 cycles of 94°C for 30 s, annealing at 48°C for 60 s, and extension at 72°C for 90 s, and then followed by 30 cycles of 94°C for 30 s, annealing at 50°C for 60 s, and extension at 72°C for 90 s, and a final extension at 72°C for 10 min. PCR products were checked by electrophoresis using 1.5% agarose gel and purified using commercial Gel/PCR purification kit (QIAGEN─QIAquick PCR purification kit). Sequences were sent for Sanger sequencing using an ABI 3730 automated sequencer. All sequences were edited, translated into amino acid using MEGA 11 [37].

- **Data analyses**

Species were confirmed through a dual process involving both morphological identification and cross-referencing with reference sequences available in the National Center for Biotechnology Information (NCBI) and the Barcode of Life Database (BOLD system). Taxonomic name changes, particularly in the family Dasyatidae, were also recognized [38]. The differences in the generic names of species in the genera previously named as *Himantura* and *Dasyatis*, were not treated as different from the newly assigned names. In instances where matches were inconclusive, a thorough assessment was conducted using supplementary information, notably morphology and geographic data. In cases of persistent uncertainty, the designation "cf." was attributed to those particular samples. The validation of species through molecular means relied on a similarity scoring system, demanding a minimum of 95% query coverage and a similarity level of 98% or higher. The 95% query coverage threshold was selected for its robustness and greater likelihood of yielding accurate matches. A similarity level of 98% or higher was chosen because intraspecific genetic variation in most fish species studied to date typically falls within this range [13, 15, 20–22]. This methodology utilized the BLAST algorithm (NCBI) and BOLD Identification Engine. Nevertheless, COI fragments from our samples did not overlap with most *Glaucostegus* COI sequences in the public database due to very poor DNA quality resulting in sequences that did not align well with published reference sequences, preventing the application of a 95% query coverage threshold. Instead, a minimum coverage of 60% or higher was used to achieve the best possible results. A separate file was created for *Glaucostegus* tree construction. Following these comprehensive validations, the genetic sequences were duly submitted to the NCBI database, contributing to the broader body of scientific knowledge and facilitating further research and insights into the studied species (S2 file).

To assess relationships between the samples and reference data, phylogenetic trees were constructed. Sequences were aligned in MEGA 11 and saved as FASTA files. For COI analysis in sharks, sequences of *Rhynchobatus australiae* and *Glaucostegus granulatus* served as outgroups, while that of *R. australiae* was used for ND2. In batoids, sequences of *Chiloscyllium punctatum* and *C. griseum* were used as outgroups for COI and ND2, respectively. The model for the sequence alignment was selected using IQ-TREE. ModelFinder [39]. This method, integrated within IQ-TREE V2.3.6, was employed to identify the best substitution model based on the Bayesian Information Criterion (BIC), which was TIM2+F+I+G4. Bayesian inference of phylogeny was conducted using MrBayes (v3.2.7) [40]. A Markov Chain Monte Carlo (MCMC) analysis was executed in MrBayes with a total of 1,000,000 generations. The posterior

distribution was sampled every 100 generations. Four chains were used to improve exploration of the posterior distribution. After the analysis, 25% of the samples were discarded as burn-in to eliminate the influence of the starting state. Convergence diagnostics, including the standard deviation of split frequencies and effective sample sizes (ESS), were assessed to ensure reliability of the analysis. The consensus tree was generated by Figtree V1.4.4 with posterior probabilities representing the clade support [41].

These trees offered a visual representation of the evolutionary placements for both sharks and batoids. The genetic distances were quantified using the Kimura-2-Parameter (K2P) method, gauging the variations of intra- and inter- specific diversity of species and genera of samples in this study. For this analysis, the reference sequences of unidentified species of reference sequences were excluded. Because several species in this study were represented by a small sample size, sequences in NCBI and BOLD databases under the same scientific names were used to enable intraspecific comparison.

Results

There were 153 samples with successful amplification of either COI or ND2 fragments or both. The two DNA barcoding fragments offered different advantages (Table 1). COI fragments showed better rate of amplification success than ND2, while ND2 provided more characteristics for comparison. A larger number of ND2 sequences could not be matched with any references in the database, whereas most COI sequences found their matches, but some matched sequences attributed to more than one species and were therefore inconclusive. Nevertheless, the matching results did not indicate correct species identification because conclusive species identification was conducted using morphological examination. The genetic data only showed possible identification if only DNA sequences were available.

Table 1. Comparisons of COI and ND2 fragments as DNA barcoding for elasmobranch species identification from 153 samples.

criteria	COI	ND2
number of sequences of successful amplification	130	99
length of fragments obtained (bp)	520-676	970-1047
number of sequences NOT matched with any reference sequences or matched unidentified species	9	31
number of sequences matched with multiple species	26	0

With the combined use of morphological and genetic data, the samples in this study unveiled a total of 28 shark species and 32 batoid species. The process of arriving at consensus species heavily relied on morphological traits, with genetic data serving a supplementary role. Identification to species was congruent when assessed through both morphological and genetic examination when allowing for intraspecific genetic variations of no more than 2% (Table 2; Figs 1– 4). Phylogenetic analysis of a sample assigned to *Orectolobus leptolineatus* on morphological criteria matched an unidentified *Orectolobus* sequence (Fig 2A).

Figure 1. Phylogenetic tree of sharks based on 654 bp of COI fragments; (A) overall relationships of sharks in this study (B) phylogenetic tree of sharks in the order Carcharhiniformes; bold = samples in this study; number on lines indicating bootstrap values.

Figure 2. Phylogenetic tree of sharks based on 1044 bp of ND2 fragments; (A) overall relationships of sharks in this study (B) phylogenetic tree of sharks in the order Carcharhinoformes; bold = samples in this study; number on lines indicating bootstrap values.

Figure 3. Phylogenetic tree of batoids based on 652 bp of COI fragments; (A) overall relationships of batoids in this study (B) phylogenetic tree of batoids in the family Dasyatidae; (C) phylogenetic tree of batoids in the genus *Glaucostegus*, bold = samples in this study, sequences MW431029, MW431018 identified as *G. younholeei* (Habib and Islam 2021).; number on lines indicating bootstrap values.

Figure 4. Phylogenetic tree of batoids based on 1044 bp of ND2 fragments; (A) overall relationships of batoids in this study (B) phylogenetic tree of sharks in the family Dasyatidae; bold = samples in this study; number on lines indicating bootstrap values.

Table 2. Species identification based on morphological and genetic characteristics.

Morphological identification	genetic identification based on COI and/or ND2 (best match)	Phylogenetic tree placement	consensus species	problem
**SHARKS**				
ECHINORHINIFORMES				
Echinorhinus brucus	*Echinorhinus* sp.	*Echinorhinus* sp.	*E. brucus*	Insufficient information for comparison
SQUALIFORMES				
Squalus hemipinnis	not matched	*S. brevirostris*	*S. hemipinnis*[*1]	Insufficient information for comparison
Squalus montalbani	*Squalus* sp.	*Squalus* sp. A	*S. montalbani*	Insufficient information for comparison
Centrophorus uyato	*C. granulosus/ C. zeehaani/ C. uyato*	*Cen. uyato*	*C. uyato*[*2]	Taxonomic revision
Etmopterus fusus	not matched	Not group with any species (COI); *E. splendidus* (ND2)	*E. fusus*	Discordance between morphology and genetics
ORECTOLOBIFORMES				
Orectolobus leptolineatus	*O.* cf. *leptolineatus*	*O. leptolineatus*	*O. leptolineatus*	None
Chiloscyllium griseum	*C. griseum*	*C. hasselti/ C. griseum*	*C. griseum*	None
Chiloscyllium hasselti	*C. hasselti/ C. griseum*	*C. hasselti/ C. griseum*	*C. hasselti*	Cryptic morphological similarity
Chiloscyllium indicum	*C. indicum*	*C. indicum*	*C. indicum*	None
Chiloscyllium punctatum	*C. punctatum*	*C. punctatum*	*C. punctatum*	None
Stegostoma tigrinum	*S. fasciatum*	*S. fasciatum*	*S. tigrinum*[*3]	None
LAMNIFORMES				
Isurus oxyrinchus	*I. oxyrinchus*	*I. oxyrinchus*	*I. oxyrinchus*	None
CARCHARHINIFORMES				
Atelomycterus marmoratus	*A. marmoratus*	*A. marmoratus*	*A. marmoratus*	None
Bythaelurus lutarius	*B. hispidus*	*B. hispidus* (COI); not grouped with any species (ND2)	*B.* cf. *lutarius*	Discordance between morphology and genetics
Iago mangalorensis	*Iago* sp.	*Iago* sp. A	*I. gopalakrishnani*[*4]	Taxonomic revision
Mustelus stevensi	*Mustelus* sp./ *M. lenticulatus/ M. stevensi*	*M. lenticulatus* (COI); *M. stevensi* (ND2)	*M. stevensi*	Cryptic morphological similarity
Carcharhinus amboinensis	*C. amboinensis*	*C. amboinensis*	*C. amboinensis*	None
Carcharhinus brevipinna	*C. brevipinna*	*C. brevipinna*	*C. brevipinna*	None
Carcharhinus falciformis	*C. falciformis/ C. brevipinna*	*C. falciformis*	*C. falciformis*	Cryptic morphological similarity
Carcharhinus leucas	*C. leucas*	*C. leucas*	*C. leucas*	None

Morphological identification	genetic identification based on COI and/or ND2 (best match)	Phylogenetic tree placement	consensus species	problem
Carcharhinus limbatus	*C. limbatus*	*C. limbatus*	*C. limbatus*	None
Carcharhinus longimanus	*C. longimanus*	*C. longimanus*	*C. longimanus*	None
Carcharhinus melanopterus	*C. melanopterus*	*C. melanopterus*	*C. melanopterus*	None
Carcharhinus sorrah	*C. sorrah/ C. sealei*	*C. sorrah*	*C. sorrah*	Cryptic morphological similarity
Prionace glauca	*P. glauca*	*P. glauca*	*P. glauca*	None
Galeocerdo cuvier	*G. cuvier*	*G. cuvier*	*G. cuvier*	None
Sphyrna lewini	*S. lewini*	*S. lewini*	*S. lewini*	None
Sphyrna mokarran	*S. mokarran*	*S. mokarran*	*S. mokarran*	None
**BATOIDS**				
RHINOPRISTIFORMES				
Rhynchobatus australiae	*R. australiae*	*R. australiae*	*R. australiae*	None
Rhinobatos ranongensis	no matched	*Rhinobatos* cf. *borneensis*	*R. ranongensis*	Insufficient information for comparison
Glaucostegus younholeei	*Glaucostegus* sp. (later as *G. younholeei*)/ *G. granulosus*	*Glaucostegus* sp./ *G. granulosus*	*G. younholeei*[*5]	Cryptic morphological similarity
Platyrhina psomadakisi	not matched	not grouped with any species	*P. psomadakisi*	Insufficient information for comparison
TORPEDINIFORMES				
Torpedo sinuspersici	not matched	*Torpedo* sp./ *T. sinuspersici*	*T. sinuspersici*	Insufficient information for comparison
Benthobatis moresbyi	*B. moresbyi*	*B. moresbyi* (COI); not grouped with any species (ND2)	*B. moresbyi*	None
Narcine maculata	*N. maculata*	*N. maculata*	*N. maculata*	None
Narcine prodorsalis	*N. maculata*/ *Narcine* sp./ *N.* cf. *oculifer*	*Narcine* sp.	*N. prodorsalis*	Cryptic morphological similarity
Narcine timlei	*N. timlei/ N. brunnea*	*N. timlei*	*N. timlei*	Cryptic morphological similarity
RAJIFORMES				
Orbiraja powelli	*O. powelli*	*O. powelli*	*O. powelli*	None
Cruriraja andamanica	not matched	*Cruriraja triangularis* (COI); *C. hulleyi* (ND2)	*C. andamanica*	Insufficient information for comparison
MYLIOBATIFORMES				
Hexatrygon bickelli	*H. bickelli*	*H. bickelli*	*H. bickelli*	None
Gymnura poecilura	*G. poecilura*	*G. poecilura*	*G. poecilura*	None

Morphological identification	genetic identification based on COI and/or ND2 (best match)	Phylogenetic tree placement	consensus species	problem
Brevitrygon heterura	*B.* (*Himantura*) *walga*/ *B. heterura*	*B. walga* / *B. heterura*	*B. heterura*[*6]	Taxonomic revision
Hemitrygon bennetti	*H.* (*Dasyatis*) *bennetti*	*H. bennetti*	*H. bennetti*	None
Hemitrygon laosensis	not matched	not grouped with any species	*H. laosensis*	Insufficient information for comparison
Himantula undulata	*H. undulata*	*H. undulata*	*H. undulata*	None
Maculabatis gerrardi	*M. gerrardi*/ *M. macrura*	*M. macrura*/ *M. gerrardi*	*M. gerrardi*	Cryptic morphological similarity
Maculabatis pastinacoides	*M. pastinacoides*	*M. pastinacoides*	*M. pastinacoides*	None
Neotrygon caeruleopunctata	*N. kuhlii*/ *N. malaccensis*/ *N. caeruleopunctata*	*N. kuhlii* / *N. varidens*/ *N. caeruleounctata*	*Neotrygon caeruleopunctata* [*7]	Taxonomic revision
Neotrygon varidens	*N. trigonoides*/ *N. kuhlii*/ *N.* cf. *kuhlii*	*N. kuhlii*/ *N. trigonoides*	*N. varidens*[*7]	Taxonomic revision
Pateobatis jenkinsii	*P.* (*Himantura*) *jenkinsii*	*P. jenkinsii*	*P. jenkinsii*	None
Pateobatis uarnacoides	*P.* (*Himantura*) *uarnacoides*	*P. uarnacoides*	*P. uarnacoides*	None
Pteroplatytrygon violacea	*P. violacea*	*P. violacea*	*P. violacea*	None
Taeniura lymma	*T. lymma*	*T. lymma*	*T. lymma*	None
Telatrygon biasa	*T.* (*Dasyatis*) *zugei*/ *T. biasa*	*T.* (*Dasyatis*) *zugei* (COI); *T.* (*Dasyatis*) *zugei* / *T. biasa* (ND2)	*T. biasa*[*8]	Taxonomic revision
Telatrygon crozieri	*T.* (*Dasyatis*) *zugei*	*T.* (*Dasyatis*) *zugei* / *T. biasa*	*T. crozieri*[*8]	Taxonomic revision
Urogymnus lobistoma	*U. lobistoma*	*U. lobistoma*	*U. lobistoma*	None
Myliobatis hamlyni	*M. tobijei*	*M. tobijei*	*M. hamlyni*[*9]	Taxonomic revision
Aetobatus ocellatus	*A. ocellatus*/ *A. narinari*	*A. ocellatus*	*A. ocellatus*[*10]	Taxonomic revision
Rhinoptera jayakari	*R. jayakari*	*R. jayakari*	*R. jayakari*	None
Mobula tarapacana	*M. tarapacana*	*M. tarapacana*	*M. tarapacana*	None

*1 short ND2 fragment comparison = *S. hemipinnis*

*2 taxonomic revision—*C. zeehaani* = syn. of *C. uyato* often misidentified with *C. granulosus* [42]

*3 listed as *Stegostoma fasciatum* in BOLD, listed as *S. fasciatum* and *S. tigrinum* in NCBI

*4 taxonomic revision and species resurrection [43]

*5 *Glaucostegus* sp. in GenBank described as *G. younholeei* sp. nov. in Habib and Islam [44]

*6 taxonomic revision and geographic distribution of *Brevitrygon* [38, 45]

*7 taxonomic revision of *Neotrygon kuhlii* [46-48]

*8 taxonomic revision and species resurrection [34]

*9 taxonomic revision and species resurrection [49]

*10 taxonomic revision and species resurrection [50]

Not all samples could be identified. Problems regarding species identification could be grouped into 4 categories.

1. Insufficient information for comparison/ lack of species registered in the database

Specific challenges emerged in cases where information on references was unavailable within established databases. In these instances, the primary criterion for species identification was based on morphological characteristics of the samples. These samples included rarely found species such as *Hemitrygon laosensis*, *Echinorhinus brucus*, *Squalus montalbani*, *Platyrhina psomadakisi*, *Torpedo sinuspersici*, and *Cruriraja andamanica*. Genetic data for some species such as *Rhinobatos ranongensis* were not available for public access. In these cases, we relied solely on morphology for species identification.

For *Squalus hemipinnis*, the reference sequences of which did not fully meet the sequence alignment criteria of this study, and did not align with any known species. Although the phylogenetic tree placed it in close proximity to *S. brevirostris*, there was a 2.5% genetic difference. An alignment of short fragments of ND2 of our specimen and sequences in the NCBI database (529 base pairs—not shown) listed under *S. hemipinnis* revealed their similarity. This information was omitted and not presented here. By combining morphological characteristics and partial DNA barcoding data, the consensus identification for this sample was determined to be *S. hemipinnis*.

2. Taxonomic revision/ redescription/ new species assignments

Taxonomic revisions present yet another challenge that impedes the comprehensive utilization of DNA barcodes in databases. Many of these barcodes were deposited prior to taxonomic revisions and have not been updated, resulting in classification of samples under different names. Such instances were observed in *Centrophorus uyato*, several species of rays in the family Dasyatidae and *Aetobatus ocellatus*, where they were matched with species bearing the names before revisions. Consensus as to species was drawn based on morphological characteristics and published distributional ranges of taxa in current literature. The names used when the samples were deposited were likely synonyms and require updating. Our DNA sequence data represented and comparable with recent DNA information deposited after the revision. Nevertheless, some revised taxa, namely *T. crozieri*, lacked references for their DNA barcodes. Given the unavailability of morphology data for these references, we attempted to establish DNA barcoding profiles that aligned with their corresponding morphological characteristics under the assigned names.

The samples of *Glaucostegus* in this study were previously recognized as *Glaucostegus* cf. *granulosus* due to their genetic similarity to this species but still showed morphological differences. Our DNA sequences matched an undescribed *Glaucostegus* sp. later named as a distinct species. The updated publication of the sequence information in the NCBI website allowed us to place the valid name on these samples as *G. younholeei* [44].

The reassignment of species in the genus *Myliobatis* clarified the presence of two closely related species, *M. hamlyni* and *M. tubijei*. *Myliobatis hamlyni* was previously considered a junior synonym of *M. tobijei* but later confirmed as a valid species. The name applied to sequences of *M. tobijei* that matched with our sample were likely following the original

designation, not subsequently updated. No sequences of *M. hamlyni* were available for comparison with our data. Based on morphological examination and distribution range, we conclude that the consensus species of this sample is *M. hamlyni*.

The houndshark sample was initially identified as *Iago mangalorensis* based on morphological similarities, which more closely resembled this species than other members of the genus *Iago*, prior to the formal description of the new species *I. gopalakrishnani* [43]. At the time, genetic data for *I. mangalorensis* were not available for comparison. Although our sample clustered with several undescribed *Iago* species, it was provisionally designated as *I. mangalorensis*. Following the formal description of *I. gopalakrishnani*, the sample is now considered to belong to this newly recognized species.

3. Cryptic morphological similarity

There were discrepancies in the labeling of sequences when comparing the NCBI and BOLD databases for a few species, such as *Chiloscyllium hasseltii*, which closely resembles *Chiloscyllium griseum*; *Carcharhinus falciformis*, misidentified as *Carcharhinus. brevipinna; Carcharhinus sorrah*, misidentified as *Carcharhinus seali*; and *Maculabatis gerrardi*, occasionally misidentified as *M. macrura*. These species pairs highly similar may be misidentified by non-specialists. For example, *Chiloscyllium hasseltii* and *Chiloscyllium griseum* are morphologically similar as adults but can be distinguished in the juvenile stage based on the presence of dark brown bands. Similarly, *M. gerrardi* and *M. macrura* both exhibit white spots [illegible] their bodies but differ in the distribution of these markings and the morphometric ratios (head length to disc width and disc length to disc width).

Some species are unfamiliar and sometimes misidentified as related species such as *Mustelus stevensi* occasionally misidentified as *M. lenticulatus*, *Narcine timlei* occasionally misidentified as *N. brunnea*. These samples exhibited distinct morphological characteristics from their congeners. In such cases, we placed reliance on the morphological characteristics of these species, recognizing the potential for mislabeling in the references. Furthermore, we cross-referenced synonyms of these species with available literature, giving priority to accepted names. Consequently, the consensus species identification remained consistent with the initially assigned species name.

Sequences under name *Narcine prodorsalis* were either not present or unavailable for public access in both NCBI and BOLD. However, our *N. prodorsalis* COI sequence matched with an undescribed *Narcine* sp. and one *N. maculata*. The phylogenetic placement of our sample and the other *Narcine* species showed that the *N. maculata* that matched our sequence was likely mislabeled because all other *N. maculata* reference sequences formed a distinct clade.

4. Discordance between morphology and genetics

We also encountered discrepancies between morphology and genetics as in the case of *Bythaelurus* cf. *lutarius*. This species exhibited clear morphological differences from its counterpart, *B. hispidus*, and other congeners. However, their COI sequences aligned with that of *B. hispidus* (Fig 1B) while ND2 sequences did not align with any *Bythaelurus* species (Fig 2B). These morphological distinctions included the absence of papillae in the tongue and roof of the mouth, variations in head shape and differences in the origins of the dorsal fins [5]. As a result, a

consensus identification could not be definitively established, and the specimens were provisionally assigned as *B.* cf. *lutarius*.

Another challenging case was found in a sample morphologically identified as *Etmopterus fusus*. The COI sequence of this sample did not match any references in both NCBI and BOLD while its ND2 sequence matched an *E. splendidus* sample from Taiwan. Our sample from the Andaman Sea displayed clear morphological characteristics consistent with *E. fusus*, including the presence of four black bands above the anal fin continuing to the caudal fin. This feature distinguished it from *E. splendidus*, which displayed a different black band pattern in the same position. The distribution ranges of these 2 species are clearly different with *E. fusus* in the Eastern Indian Ocean and northern Western Australia, and *E. splendidus* in the Northwestern Pacific Ocean. The single reference COI sequence of *E. fusus* which came from a sample in New Caledonia showed a genetic distance from our sample greater than 6% based on 659 bp. Based on the morphological characteristics and the collecting locality of our sample, the consensus identification was *E. fusus*.

The average intraspecific genetic distance for sharks and batoids across both gene fragments, COI and ND2, was about 1% (Table 3). However, the maximum intraspecific genetic distance was much greater. In sharks, the highest COI variation was between our *Etmopterus fusus* sample and references. A similar ND2 pattern was seen in *Echinorhinus brucus*. Among batoids, samples of *Brevitrygon imbricata* and *Aetobatus flagellum* presented highest intraspecific genetic distances for both genes. Notably, *Himantura leoparda* and *Narcine* spp. showed COI intraspecific variation exceeding 4%. The average genetic differentiation among congeneric species exceeded 7% for both gene fragments.

Table 3. Genetic distance (K2P percentage) based on COI and ND2 of elasmobranch species.

**Levels of genetic comparison**	**Taxa**	**COI**			**ND2**		
		Mean (S.E.)	Min	Max	Mean (S.E.)	Min	Max
Variations among individual of the same species	Sharks	0.92 (0.26)	0.00	6.32	0.76 (0.21)	0.00	5.37
	Batoids	0.86 (0.13)	0.00	5.12	1.26 (0.31)	0.00	10.49
Variations among species of the same genus	Sharks	7.50 (0.54)	0.05	17.73	10.88 (0.57)	0.39	21.01
	Batoids	10.42 (0.45)	0.21	26.40	11.31 (0.59)	0.58	32.38

However, very low interspecific genetic distances were observed between our *Etmopterus fusus* sample and the NCBI reference of *E. splendidus* and between our *Mustelus stevensi* sample and the reference *M. lenticulatus*. Among batoids, very low interspecific genetic variation was observed in *Neotrygon kuhlii* and other *Neotrygon* species and also within the genus *Telatrygon*. Similarly, low genetic variation was observed among species of *Rhinoptera*, *Aetobatus*, *Mobula*, and *Myliobatis* in the NCBI reference sequences.

The distribution of K2P genetic distances in sharks and batoids revealed distinct differences between intra- and interspecific variation (Figs 5, 6). Over 80% of our samples fell

within 2% genetic distance of their assigned species. However, interspecific genetic variation varied across both genes. Some genera, such as *Etmopterus* and *Echinorhinus*, showed highly similar sequences, likely due to limited available genetic data. In contrast, other genera, such as *Neotrygon*, *Telatrygon*, and *Aetobatus*, have undergone taxonomically revisions, and current scientific names of many sequences have not been updated.

Sequences from our samples of *Rhinobatos ranongensis*, *Cruriraja andamanica*, and *Hemitrygon laosensis*, did not match any published species since reference sequences were unavailable. These DNA sequences showed distinct genetic diversity compared to their congeneric species.

Figure 5. Distribution of K2P genetic distance in sharks (A) COI, arrow pointing to *Etmopterus fusus*; (B) ND2, arrow pointing to *Echinorhinus brucus*.

Figure 6. Distribution of K2P genetic distance in batoids (A) COI, arrows pointing *to Narcine timlei*, *N. brunnea*, *Brevitrygon imbricata*; (B) ND2 arrows pointing to *Brevitrygon imbricata*, *Aetobatus flagellum*.

Discussion

The implementation of DNA barcoding significantly enhances the taxonomic clarification of cartilaginous fishes, offering an alternative to the intricate and time-consuming process of species identification for non-taxonomists [15, 51]. However, morphological examination still remains the key method. Among the well-recognized mitochondrial gene candidates serving as DNA barcodes for cartilaginous fishes, COI and ND2 stand out [7, 12, 13, 15, 20]. COI tends to demonstrate higher success rates in amplification compared to ND2 due to its shorter length. The design of shorter fragments within the ND2 region amplification process has bolstered its efficacy, providing additional and valuable insights for future application. It is important not to pass judgment on the superiority of one gene over the other, as each presents distinct advantages. COI has enjoyed a decade-long legacy as a DNA barcode for fishes [12, 13, 15, 21, 22], whereas the acceptance of ND2 as a DNA barcode for chondrichthyans came later [7, 52]. The extensive utilization of COI is mirrored by the larger repository of COI sequences available across databases in comparison to ND2 sequences. However, ND2 sequences offer the advantage of increased length, subsequently affording more characteristics for meaningful comparison. Consequently, a synergistic employment of both genes holds promise in the accurate assessment of elasmobranch diversity. The number of ND2 sequences available for comparison is fewer than COI. COI still serves as important DNA barcode but need serious taxonomic updating.

The utility of DNA barcoding has contributed to new species records [5, 22, 53, 54]. In this study, DNA barcoding clarified specimens initially identified as *Glaucostegus* cf. *granulatus* [5], confirming them as *Glaucostegus younholeei* [44]. Additionally, DNA barcoding aids in

identifying cryptic species, leading to new species descriptions and taxonomic revisions [e.g.55-61].

Despite the considerable promise of DNA barcodes, this study has revealed several limitations. The effectiveness of this tool is contingent upon the availability of data from verified species, which is absent or limited from, especially, deep-sea species. Because of the rarity of specimens, identification based on morphological criteria may be mistaken resulting in erroneous molecular sequence assignment. The description of a new species, *Glaucostegus younholeei* sp. nov. [44], provided a clear identification of samples of the guitarfish previously described as *Glaucostegus* cf. *granulatus* [5] although the reference sequences in NCBI have not been updated and listed the species as *Glaucostegus* sp. The authors of the new species have included a reference to the description of their newly described species as an update, hence providing an official name for the taxon. In addition, the presence of synonyms for certain species, such as the zebra shark, has also presented a problem, with two names existing in the databases. Although a priority is accorded to the senior synonym, *Stegostoma tigrinum* [55], several sequences of this species are still listed as *S. fasciatum*.

The issue of cryptic morphological similarity, due to either specimen misidentification or failure to take account of taxonomic revision, also presents a significant challenge when utilizing public databases for species verification. In such cases, it becomes necessary to rely on the morphological characteristics of specimens to confirm species assignment. The landscape of taxonomic revisions within many taxa adds complexity to the direct utilization of DNA barcodes, as species names undergo alterations over time. For example, DNA barcodes of samples identified as *Centrophorus uyato* have been matched with different *Centrophorus* species due to recent revisions that revealed *C. granulosus* as a species complex [42, 56]. As a result, several COI of several species including *C. uyato* were erroneously labeled as *C. granulosus*. This problem is also observed in *Maculabatis gerrardi* complex [57]. On the contrary, information on ND2 sequences is generally more recent and up-to-date serving as a better reference than COI. A similar situation was found in *Aetobatus ocellatus* that was previously grouped within the *A. narinari* species complex but later recognized as a valid species based on molecular evidence [50, 58]. Sequences of samples labeled as *A. narinari* prior to this taxonomic revision could actually represent *A. ocellatus*, emphasizing the need for caution when using such references.

The extensive revision of stingrays of the family Dasyatidae has brought about significant changes in generic names and the erection of new species exemplified by *Brevitrygon* [45], *Neotrygon* [47], and *Telatrygon* [59]. These species belonging to these genera exhibit distinct morphological characteristics that serve as a primary mean of identification, albeit requiring the expertise of specialists for their precise differentiation. To accelerate the identification process, DNA profiles of these species should be established. This study demonstrated such genetic distinctions through DNA barcoding, which contributes to the improved assessment of biodiversity.

Several sharks and rays are still subject to taxonomic revision due to their morphological similarity and very wide distribution ranges. The *Squalus* species complex presents wide distribution range and similar morphological characteristics among congeners [60-62]. Some species are thought to be endemic to certain geographic locations, but their distribution is still unclear and requires further examination, which may result in further taxonomic revision [36]. Our *Squalus* samples provisionally identified as *S. hemipinnis* and *S. montalbani*, which did not match the reference sequences given for those taxa may be distinct lineages.

Discordance between morphology and the DNA barcode assigned to *Bythaelurus* cf. *lutarius* provided difficulty in reaching consensus identification. Identification based on morphological characteristics revealed that all examined samples collected from the Thai Andaman Sea were of *B. lutarius* [5]. This species, first described as *Halaelurus lutarius* [63], is clearly distinguishable from similar looking species in the same genus [64-69]. The lack of COI sequences of *B. lutarius* from the type locality and the unavailability of the ND2 sequence of *B. hispidus*, despite being studied [67], poses a challenge to the use of DNA barcoding for species confirmation. Because all *B.* cf. *lutarius* sampled formed a monophyletic cluster based on longer ND2 fragments, clearly distinct from other catsharks whose sequences were available for comparison, they likely all represented the same species. Although the samples show clear morphological distinction, their location falls outside the known distribution range of *B. lutarius* and their genetic affinity within this genus remains uncertain, the samples are tentatively assigned as *B.* cf. *lutarius*.

It has been long recognized that intraspecific genetic variations among metazoans generally fall within 2% [13, 15, 20–22]. However, low genetic variation among distinct but congeneric lineages is evident in *Mustelus* species [70]. The clear genetic differentiation between *M. stevensi* and *M. lenticulatus* [10] enabled accurate consensus identification. On the contrary, certain species, such as *Etmopterus fusus*, *Squalus montalbani*, *Himantura leoparda*, *Maculabatis gerrardi*, and *Narcine timlei*, display high genetic variation within species. This may arise from either cryptic diversity or misidentification of reference samples as seen in the species complex of *Himantura uarnak* [71], *Maculabatis gerrardi* [57], and *Narcine* species [72]. Therefore, while a 2% genetic difference within species is helpful, it cannot serve as an absolute criterion for species delimitation. Comparisons of genetic differentiation based on COI and ND2 fragments showed slightly greater interspecific differences in ND2 compared to COI possibly due to the utilization of longer fragments in the analyses. Nevertheless, low genetic differentiation among distinct congeneric species may confound their recognition. For example, our *Etmopterus fusus* sample showed high COI sequence variation compared to reference *E. fusus* but similar a ND2 sequence compared to *E. splendidus*. Although both species are closely related and rarely available for thorough examinations [73], clear difference in their geographical distribution [74] helps identify our sample as *E. fusus*. While the influx of DNA barcoding sequences is expected to continue, its application should be approached with caution. Given the dearth of expert chondrichthyan taxonomists at both local and global scales, the utilization of

molecular markers is important for facilitating biodiversity assessment [75]. The data gathered by our present study contribute both to the sample of genetic information from better-known species and new genetic information of several species not available in public databases. Despite this great benefit, the clarity of the species concept is crucial, and traditional taxonomic identification methods should not be disregarded [76].

Discordance between morphological and genetic characteristics and misidentification of closely related species may result from hybridization, which has been reported many times in elasmobranchs [77-82]. In such cases, DNA barcoding cannot resolve taxonomic identification and necessitates assessment of nuclear DNA. While hybridization and introgression have been documented in a few elasmobranch groups, many remain unexplored. Given that environmental changes may influence reproductive behaviors of these fishes, baseline data on genetics across different species are crucial for understanding these processes [80].

As elasmobranchs serve as resources for both local consumption and international export, prohibition of the use of these resources is impractical. On the other hand, regulation of fisheries based on solid scientific foundation and correct species assignment will help sustain long-term exploitation of these resources. To achieve this level of regulation, fundamental knowledge on species and genetic diversity is important and requires thorough examination, especially of local populations [18]. DNA barcoding data then serve as references for the authentication of meat products [14, 83]. However, since taxonomic revisions are ongoing and updates may not always keep pace, maintaining well-curated voucher libraries and local reference collections is essential for accurate species verification. Limited data on biodiversity jeopardize populations of these fishes, especially those commonly used in the preparation of luxurious dishes and pharmaceutical products, which usually come from non-specified species and some of which are globally vulnerable species [84-88].

Acknowledgements

We would like to thank our assistants, P. Chanmuang. T. Meejan, P. Laongdee, K. Panpong, A. Pankhort, and P. Wongmanee, who contributed to sample collection and laboratory work. Several organizations and individuals provided specimens for this project, including the Marine Fisheries Research and Development Division and Inland Fisheries Research Development Division of the Department of Fisheries, the Thailand National Science Museum, Department of Marine and Coastal Resources, C. Vidthayanon, and T. Vasinopas. This project is funded by National Research Council of Thailand (NRCT-N25A650485). Language editing was assisted by P. D. Round. The authors declare that they do not have any known financial interests or personal relationships that could have appeared to influence the work reported in this paper.

Financial disclosure

The funders had no role in study design, data collection and analysis, decision to publish, or preparation of the manuscript.

References

1. Weigmann S. Annotated checklist of the living sharks, batoids and chimaeras (Chondrichthyes) of the world, with a focus on biogeographical diversity: Annotated global checklist of Chondrichthyes. J Fish Biol. 2016;88(3):837–1037.
2. Ali A, Fahmi, Dharmadi, Krajangdara T, Khiok ALP. Biodiversity and habitat preferences of living sharks in the Southeast Asian region. Indones Fish Res J. 2018; 24: 133-140.
3. Voris HK. Maps of Pleistocene sea levels in Southeast Asia: shorelines, river systems and time durations. J Biogeogr. 2000;27(5):1153–67.
4. Costello MJ, Tsai P, Wong PS, Cheung AKL, Basher Z, Chaudhary C. Marine biogeographic realms and species endemicity. Nat Commun. 2017;8(1):1057.
5. Krajangdara T, Vidthayanon C, Ali A, Lim A, Sumontha M, Rodpradit R, et al. The Cartilaginous Fishes of Thailand and Adjacent Waters. Department of Fisheries, Ministry of Agriculture and Cooperatives. Chiang Mai, Thailand: Vanidakarnpim Publishing; 2022. Thai.
6. Krajangdara T, Fahmi, Ebert DA, Chaorattana C, and Khudamrongsawat J. Morphological and genetic evidence confirmed three new records of ghost shark species (Chimaeriformes) from the Andaman Sea of Thailand. Trop Nat His. 2021;21:218-233.
7. Naylor GJP, Caira JN, Jensen K, Rosana KAM, White WT, Last PR. A DNA sequence–based approach to the identification of shark and ray species and its implications for global elasmobranch diversity and parasitology. B Am Mus Nat Hist. 2012;367:1–262.
8. Khudamrongsawat J, Bhummakasikara B, Chansue N. Preliminary study of genetic diversity in the giant freshwater stingray, *Himantura chaophraya* (Batoidea: Dasyatidae) from the remnant populations in Thailand. Trop Nat His. 2017;17:175-180.
9. Last PR, Séret B, Naylor GJP. Description of *Rhinobatos ranongensis* sp. nov. (Rhinopristiformes: Rhinobatidae) from the Andaman Sea and Bay of Bengal with a review of its northern Indian Ocean congeners. Zootaxa. 2019;4576: 257–287.
10. White WT, Arunrugstichai S, Naylor GJP. Revision of the genus *Mustelus* (Carcharhiniformes: Triakidae) in the northern Indian Ocean, with description of a new species and a discussion on the validity of *M. walkeri* and *M. ravidus*. Mar Biodivers 2021;51: 42.
11. Weignmann S. Reply to Borsa (2017): Comment on ‘Annotated checklist of the living sharks, batoids and chimaeras (Chondrichthyes) of the world, with a focus on biogeographical diversity by Weigmann (2016)’. J Fish Biol. 2017;90(4):1176–1181.
12. Ward RD, Zemlak TS, Innes BH, Last PR, Hebert PDN. DNA barcoding Australia’s fish species. Philos Trans R Soc Lond B Biol Sci. 2005;360(1462):1847–57
13. Ward RD, Holmes BH, White WT, Last PR. DNA barcoding Australian chondrichthyans: results and potential uses in conservation. Mar Freshwater Res. 2008;59:57-71.
14. Holmes BH, Steinke D, Ward RD. Identification of shark and ray fins using DNA barcoding. Fish Res. 2009;95(2–3):280–288.
15. Cerutti-Pereyra F, Meekan MG, Wei N-WV, O’Shea O, Bradshaw CJA, Austin CM. Identification of rays through DNA barcoding: an application for ecologists. PLoS One. 2012;7(6):e36479.

16. Avise JC. Molecular Markers, Natural History, and Evolution. 2nd ed. Massachusetts: Sinauer, Sunderland; 2004.
17. Frankham R. Where are we in conservation genetics and where do we need to go? Conserv Genet. 2010;11(2):661–663.
18. Domingues RR, Hilsdorf AWS, Gadig OBF. The importance of considering genetic diversity in shark and ray conservation policies. Conserv Genet. 2018;19(3):501–525.
19. Moore ABM, White WT, Ward RD, Naylor GJP, Peirce R. Rediscovery and redescription of the smoothtooth blacktip shark, *Carcharhinus leiodon* (Carcharhinidae), from Kuwait, with notes on its possible conservation status. Mar Freshw Res. 2011;62(6):528.
20. Ward RD. DNA barcode divergence among species and genera of birds and fishes. Mol Ecol Resour. 2009;9(4):1077–1085.
21. Pavan-Kumar A, Gireesh-Babu P, Suresh Babu PP, Jaiswar AK, Prasad KP, Chaudhari A, et al. DNA barcoding of elasmobranchs from Indian Coast and its reliability in delineating geographically widespread specimens. Mitochondrial DNA. 2013;26(1):92–100.
22. Loh K-H, Lim K-C, Then AY-H, Adam S, Leung AJ-X, Hu W, et al. Advancing DNA barcoding to elucidate elasmobranch biodiversity in Malaysian waters. Animals. 2023;13(6):1002.
23. Kashiwagi T, Marshall AD, Bennett MB, Ovenden JR. The genetic signature of recent speciation in manta rays (*Manta alfredi* and *M. birostris*). Mol Phylogenet Evol. 2012;64(1):212–218.
24. Ariza AA, Adachi AMCL, Roque P, Hazin FHV, Vianna M, Rotundo MM, et al. DNA barcoding and species delimitation for dogfish sharks belonging to the *Squalus* genus (Squaliformes: Squalidae). Diversity. 2022;14(7):544.
25. Leray M, Knowlton N, Ho S-L, Nguyen BN, Machida RJ. GenBank is a reliable resource for 21st century biodiversity research. Proc Natl Acad Sci USA. 2019;116(45):22651–22656.
26. Pentinsaari M, Ratnasingham S, Miller SE, Hebert PDN. BOLD and GenBank revisited – Do identification errors arise in the lab or in the sequence libraries? PLoS One. 2020;15(4):e0231814.
27. Fontes JT, Vieira PE, Ekrem T, Soares P, Costa FO. BAGS: An automated Barcode, Audit & Grade System for DNA barcode reference libraries. Mol Ecol Resour. 2021;21(2):573–83.
28. Grant DM, Brodnicke OB, Evankow AM, Ferreira AO, Fontes JT, Hansen AK, et al. The future of DNA barcoding: Reflections from early career researchers. Diversity. 2021;13(7):313.
29. Mota M, Thomaz M, Fabrin C, Diamante NA, Alessandra, Filho HO, et al. DNA barcode is efficient for identifying bat species. J Mamm Evol. 2021;29(1):63–75.
30. Peláez ML, Horreo JL, García-Jiménez R, Valdecasas AG. An evaluation of errors in the mitochondrial COI sequences of Hydrachnidia (Acari, Parasitengona) in public databases. Exp Appl Acarol. 2022;86(3):371–384.
31. Baena-Bejarano N, Reina C, Martínez-Revelo DE, Medina CA, Tovar E, Uribe-Soto S, et al. Taxonomic identification accuracy from BOLD and GenBank databases using over a thousand insect DNA barcodes from Columbia. PLoS One. 2023;18: e0277379.

32. Lindner MF, Gonçalves LT, Bianchi FM, Ferrari A, Cavalleri A. Tiny insects, big troubles: a review of BOLD's COI database for Thysanooptera (Insecta). B Entomol Res. 2023;113: 703-715.
33. Ruedi M, Manzinallii J, Dietrich A, Vinciguerra L. Shortcoming of DNA barcodes: a perspective from the mammal fauna of Switzerland. Hystrix it J Mamm. 2023;34:54-61.
34. Last PR, White WT, de Carvalho MR, Séret B, Stehmann FW, Naylor GJP. Rays of the World. Ithaca: Cornell University Press; 2016.
35. Krajangdara T. The Cartilaginous Fishes (Sharks, Rays and Chimaeras) Found in Thai Waters and the Adjacent Areas. Marine Fisheries Research and Development Division, Department of Fisheries, Ministry of Agriculture and Cooperatives. Phuket: Phuketoffset Publishing; 2017. Thai.
36. Ebert, DA, Dando M, Fowler S. Sharks of the World – A Complete Guide. New Jersey: Princeton University Press; 2021.
37. Tamura K, Stecher G, Kumar S. MEGA11: Molecular Evolutionary Genetics Analysis version 11. Mol Biol Evol. 2021;38(7):3022–3027.
38. Last PR, Naylor GJP, Manjaji-Matsumoto BM. A revised classification of the family Dasyatidae (Chondrichthyes: Myliobatiformes) based on new morphological and molecular insights. Zootaxa. 2016;4139(3):345–368.
39. Kalyaanamoorthy S, Minh BQ, Wong TKF, von Haeseler A, Jermiin LS. ModelFinder: fast model selection for accurate phylogenetic estimates. Nat Methods. 2017;14(6):587–589.
40. Ronquist F, Teslenko M, van der Mark P, Ayres DL, Darling A, Höhna S, et al. MRBAYES 3.2: Efficient Bayesian phylogenetic inference and model selection across a large model space. Syst Biol. 2012;61:539-542.
41. Rambaut A. FigTree v1. 3.1. Institute of Evolutionary Biology, University of Edinburgh, Edinburgh [Internet]. 2010.
42. White WT, Guallart J, Ebert DA, Naylor GJP, Veríssimo A, Cotton CF, et al. Revision of the genus *Centrophorus* (Squaliformes: Centrophoridae): Part 3—redescription of *Centrophorus uyato* (Rafinesque) with a discussion of its complicated nomenclatural history. Zootaxa. 2022;5155:1-50.
43. Bineesh KK, Beura S, Das M, Nashad M, Akhilesh KV. Description of a new species of hound shark of the genus *Iago* (Carcharhiniformes: Triakidae) from the northern Indian Ocean. Indian J Fish. 2025;72(1):18-28.
44. Habib KA, Islam MJ. Description of a new species of giant guitarfish, *Glaucostegus younholeei* sp. nov. (Rhinopristiformes: Glaucostegidae) from the northern Bay of Bengal, Bangladesh. Zootaxa. 2021;4995(1):129–146.
45. Last PR, Weigmann S, Naylor GJ. The Indo-Pacific stingray genus *Brevitrygon* (Myliobatiformes: Dasyatidae): Clarification of historical names and description of a new species, *B. manjajiae* sp. nov., from the Western Indian Ocean. Diversity. 2023;15(12):1213.
46. Borsa P, Shen K-N, Arlyza IS, Hoareau TB. Multiple cryptic species in the blue-spotted maskray (Myliobatoidei: Dasyatidae: *Neotrygon* spp.): An update. C R Biol. 2016;339(9–10):417–26.

47. Last PR, White WT, Séret B. Taxonomic status of maskrays of the *Neotrygon kuhlii* species complex (Myliobatoidei: Dasyatidae) with the description of three new species from the Indo-West Pacific. Zootaxa. 2016;4083(4):533-561.
48. Borsa P, Arlyza IS, Hoareau TB, Shen K-N. Diagnostic description and geographic distribution of four new cryptic species of the blue-spotted maskray species complex (Myliobatoidei: Dasyatidae; *Neotrygon* spp.) based on DNA sequences. J Oceanol Limnol. 2018;36(3):827–41.
49. White WT, Kawauchi J, Corrigan S, Rochel E, Naylor GJP. Redescription of the eagle rays *Myliobatis hamlyni* Ogilby, 1911 and M. tobijei Bleeker, 1854 (Myliobatiformes: Myliobatidae) from the East Indo-West Pacific. Zootaxa. 2015;3948(3):521–48.
50. White WT, Last PR, Naylor GJP, Jensen K, Caira JN. Clarification of *Aetobatus ocellatus* (Kuhl, 1823) as a valid species, and a comparison with *Aetobatus narinari* (Euphrasen, 1790) (Rajiformes: Myliobatidae). In: Last PR, White WT, Pogonoski JJ. (Eds.), Descriptions of new sharks and rays from Borneo. CSIRO Marine and Atmospheric Research Paper 032. 2010;32:141-164.
51. Gostel MR, Kress WJ. The expanding role of DNA barcodes: Indispensable tools for ecology, evolution, and conservation. Diversity (Basel). 2022;14(3):213.
52. Henderson AC, Reeve AJ, Jabado RW, Naylor GJP. A molecular-assisted taxonomic assessment of elasmobranch fishes (Chondrichthyes: Elasmobranchii) from south-eastern Arabia revisited. Zootaxa. 2025;5575:429-446.
53. Fernando D, Bown RMK, Tanna A, Gobiraj R, Ralicki H, Jockusch EL, et al. New insights into the identities of the elasmobranch fauna of Sri Lanka. Zootaxa. 2019;4585(2):201-238.
54. Zhang M, Shan B, Liu Y, Wang L, Yang C, Liu M, et al. A new record of *Squalus montalbani* (Chondrichthyes: Squaliformes: Squalidae) from the Nansha (Spratly) Island, South China Sea. AleP. 2023;53:51-57.
55. Dahl RB, Sigsgaard EE, Mwangi G, Thomsen PF, Jørgensen RD, Torquato F de O, et al. The sandy zebra shark: A new color morph of the zebra shark *Stegostoma tigrinum*, with a redescription of the species and a revision of its nomenclature. Copeia. 2019;107(3):524.
56. White WT, Ebert DA, Naylor GJP, Ho H, Clerkin P, Verissimo A, et al. Revision of the genus *Centrophorus* (Squaliformes: Centrophoridae): Part 1—Redescription of *Centrophorus granulosus* (Bloch & Schneider), a senior synonym of *C. acus* Garman and *C. niaukang* Teng. Zootaxa. 2013;3752:035-072.
57. Muhala V, Guimarães-Costa A, Macate IE, Lutz Í, Bessa-Silva AR, et al. A possible new species of the *Maculabatis gerrardi* complex (Dasyatidae: Urogymninae) in the Indian Ocean coast off Southeastern Africa. J Mar Biol Assoc UK. 2024;104:e107,1-4.
58. Richards VP, Henning M, Witzell W, Shivji MS. Species delineation and evolutionary history of the globally distributed spotted eagle ray (*Aetobatus narinari*). J Hered. 2009;100(3):273–283.
59. Last PR, White WT, Naylor G. Three new stingrays (Myliobatiformes: Dasyatidae) from the Indo-West Pacific. Zootaxa. 2016;4147(4):377–402.
60. Marouani S, Chaâba R, Kadri H, Saidi B, Bouain A, Maltagliati F, et al. Taxonomic research on *Squalus megalops* (Macleay, 1881) and *Squalus blainvillei* (Risso, 1827)

(Chondrichthyes: Squalidae) in Tunisian waters (central Mediterranean Sea). Sci Mar. 2012;76(1):97–109.
61. Bonello JJ, Bonnici L, Ferrari A, Cariani A, Schembri PJ. Not all that clear cut: Intraspecific morphological variability in *Squalus blainville* (Risso, 1827) and implication for identification of this species. J Mar Biol Assoc UK. 2016;96(8):1585-1596.
62. Ferrari A, Di Crescenzo S, Cariani A, Crobe V, Benvenuto A, Piattoni F, et al. Puzzling over spurdogs: molecular taxonomy assessment of the *Squalus* species in the Strait of Sicily. Eur Zool J. 2021;88(1):181-190.
63. Springer S, D'Aubrey JD. Two new Scyliorhinid sharks from the east coast of Africa, with notes on related species. Oceanographic Research Institute, Investigational Report. 1972;29:1–19.
64. Springer, S. A revision of the catsharks, family Scyliorhinidae. NOAA Technical Report NMFS Circular. 1979;422:1–152.
65. Ebert DA, Clerkin PJ. A new species of deep-sea catshark (Scyliorhinidae: *Bythaelurus*) from the southwestern Indian Ocean. JOSF. 2015;15:53-63.
66. Kaschner CJ, Weigmann S, Thiel R. *Bythaelurus tenuicephalus* n. sp., a new deep-water catshark (Carcharhiniformes, Scyliorhinidae) from the western Indian Ocean. Zootaxa. 2015;4013(1):120–138.
67. Weigmann S, Ebert DA, Clerkin PJ, Stehmann MF, Naylor GJ. *Bythaelurus bachi* n. sp., a new deep-water catshark (Carcharhiniformes, Scyliorhinidae) from the southwestern Indian Ocean, with a review of *Bythaelurus* species and a key to their identification. Zootaxa. 2016;4208(5):401–432.
68. Weigmann S, Kaschner CJ. *Bythaelurus vivaldii*, a new deep-water catshark (Carcharhiniformes, Scyliorhinidae) from the northwestern Indian Ocean off Somalia. Zootaxa. 2017;4263(1):97–119.
69. Weigmann S, Kaschner CJ, Thiel R. A new microendemic species of the deep-water catshark genus *Bythaelurus* (Carcharhiniformes, Pentanchidae) from the northwestern Indian Ocean, with investigations of its feeding ecology, generic review and identification key. PLoS One. 2018;13(12):e0207887.
70. Boomer JJ, Harcourt RG, Francis MP, Stow AJ. Genetic divergence, speciation and biogeography of *Mustelus* (sharks) in the central Indo-Pacific and Australasia. Mol Phylogenet Evol. 2012;64(3):697–703.
71. Arlyza I, Shen K, Solihin DD, Soedharma D, Berrebi P, et al. Species boundaries in the *Himantura uarnak* species complex (Myliobatiformes: Dasyatidae). Mol Phylogenet Evol. 2013;66:429-435.
72. Melis R, Di Crescenzo S, Cariani A, Ferrari A, Crobe V, et al. I like this new me: unravelling population structure of Mediterranean electric rays and taxonomic uncertainties within Torpediniformes. Animals. 2023;13:2899.
73. Straube N, Iglésias SP, Sellos DY, Kriwet J, Schliewen UK. Molecular phylogeny and node time estimation of bioluminescent Lantern Sharks (Elasmobranchii: Etmopteridae). Mol Phylogenet Evol. 2010;56(3):905–917.
74. Last PR, Burgess GH, Séret B. Description of six new species of lantern-sharks of the genus *Etmopterus* (Squaloidea: Etmopteridae) from the Australasian region. Cybium. 2002;26:203-223.

75. Engel MS, Ceríaco LMP, Daniel GM, Dellapé PM, Löbl I, Marinov M, et al. The taxonomic impediment: a shortage of taxonomists, not the lack of technical approaches. Zool J Linn Soc. 2021;193(2):381–387.
76. Rheindt FE, Bouchard P, Pyle RL, Welter-Schultes F, Aescht E, Ahyong ST, et al. Tightening the requirements for species diagnoses would help integrate DNA-based descriptions in taxonomic practice. PLoS Biol. 2023;21(8):e3002251.
77. Morgan JAT, Harry AV, Welch DJ, Street R, White J, Geraghty PT, Macbeth WG, et al. Detection of interspecies hybridization in Chondrichthyes: hybrids and hybrid offspring between Australian (*Carcharhinus tilstoni*) and common (*C. limbatus*) blacktip shark found in an Australian fishery. Conserv Genet. 2012;13:455-463.
78. Cruz VP, Vera M, Mendoça FF, Pardo BG, Martinez P, Oliveira C, Foresti F. First identification of interspecies hybridization in the freshwater stingrays *Potamotrygon motoro* and *P. falkneri* (Myliobatiformes, Potamotrygonidae). Conserv Genet. 2015;16:241-245.
79. Marino IAM, Riginella E, Gristina M, Rasotto MB, Zane L, Mazzoldi C. Multiple paternity and hybridization in two smooth-hound sharks. Sci Rep. 2015;5:12919.
80. Corrigan S, Delser PM, Eddy C, Duffy C, Yang L, Li C, Bazinet AL, et al. Historical introgression drives pervasive mitochondrial admixture between two species of pelagic sharks. Mol Phylogenet Evol. 2017;110:122-126.
81. Baker AM, Adams DH, Briggers III WB, Frazier BS, Portnoy DS. Hybridization between sympatric hammerhead sharks in the western North Atlantic Ocean. Biol Lett. 2019;15:20190004.
82. Nevatte RJ, Williamson JE, Gillings MR. First evidence of multiple paternity and hybridization in Australian sawsharks. Mar Freshwater Res. 2023; 74(7):586-600.
83. Domingues RR, Bunholi IV, Pinhal D, Antuens A, Mendoça FF. From molecule to conservation: DNA-based methods to overcome frontiers in the shark and ray fin trade. Conserv Genet Resour. 2021;13:231-247.
84. Wainwright BJ, Ip YCA, Neo ML, Chang JJM, Gan CZ, Clark-Shen N, et al. DNA barcoding of traded shark fins, meat and mobulid gill plates in Singapore uncovers numerous threatened species. Conserv Genet. 2018;19(6):1393–1399.
85. Hobbs CAD, Potts RWA, Bjerregaard Walsh M, Usher J, Griffiths AM. Using DNA barcoding to investigate patterns of species utilisation in UK shark products reveals threatened species on sale. Sci Rep. 2019;9(1):1028.
86. Choo MY, Choy CPP, Ip YCA, Rao M, Huang D. Diversity and origins of giant guitarfish and wedgefish products in Singapore. Aquat Conserv. 2021;31(7):1636–1649.
87. Liu CJN, Neo S, Rengifo NM, French I, Chiang S, Ooi M, et al. Sharks in hot soup: DNA barcoding of shark species traded in Singapore. Fish Res.2021;241:105994.
88. Klangnurak W, Arunrugstichai S, Manopawitr P, Krajangdara. DNA-based species identification of shark fins traded in Thai markets. Conserv. Genet. 2023;24(4):537-546.

Fig 1A

Click here to access/download;Figure;fig1_sharks_COI_A_edit.jpg

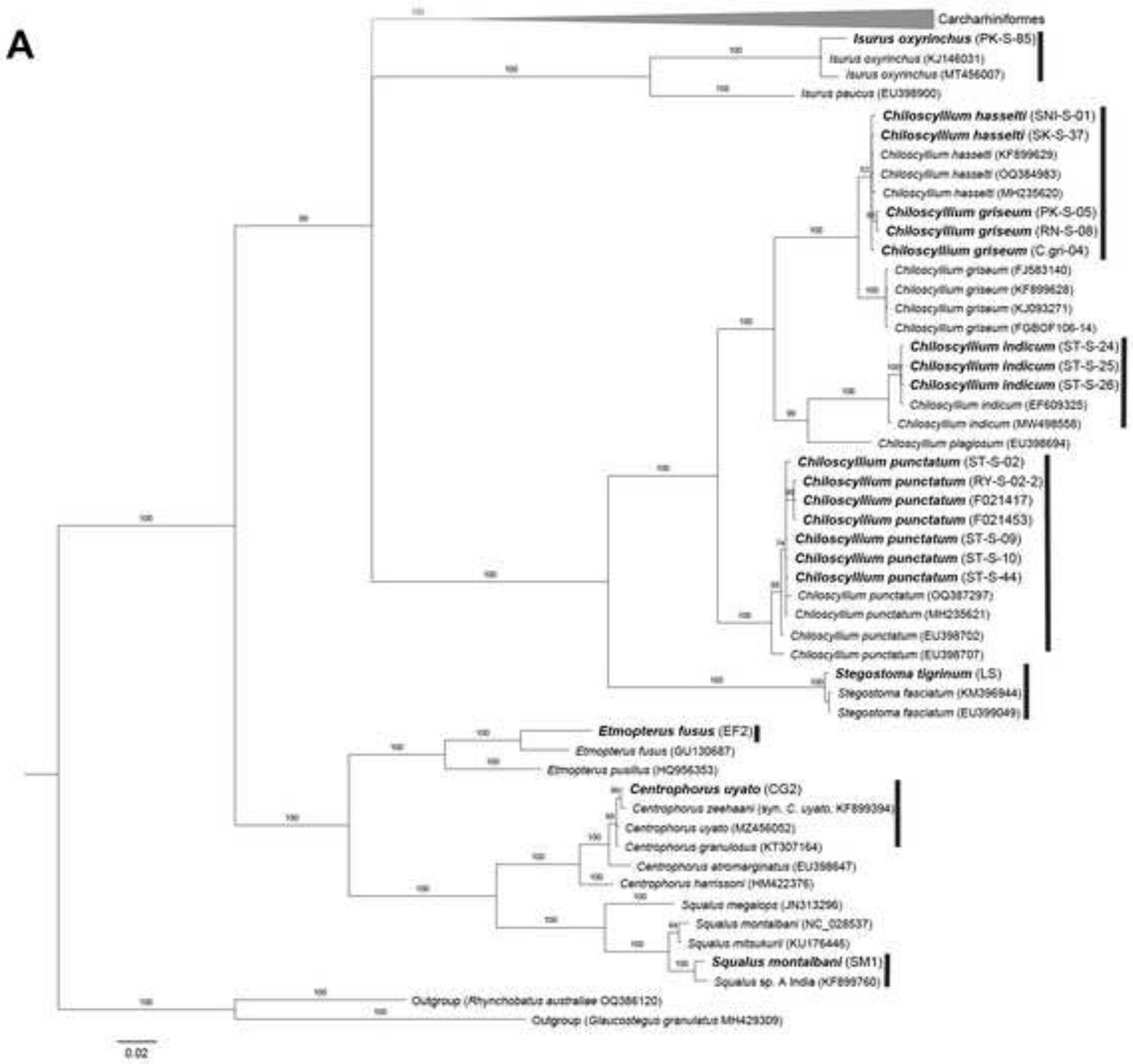

Fig 1B

Click here to
access/download;Figure;fig1_sharks_COI_B_edit.jpg

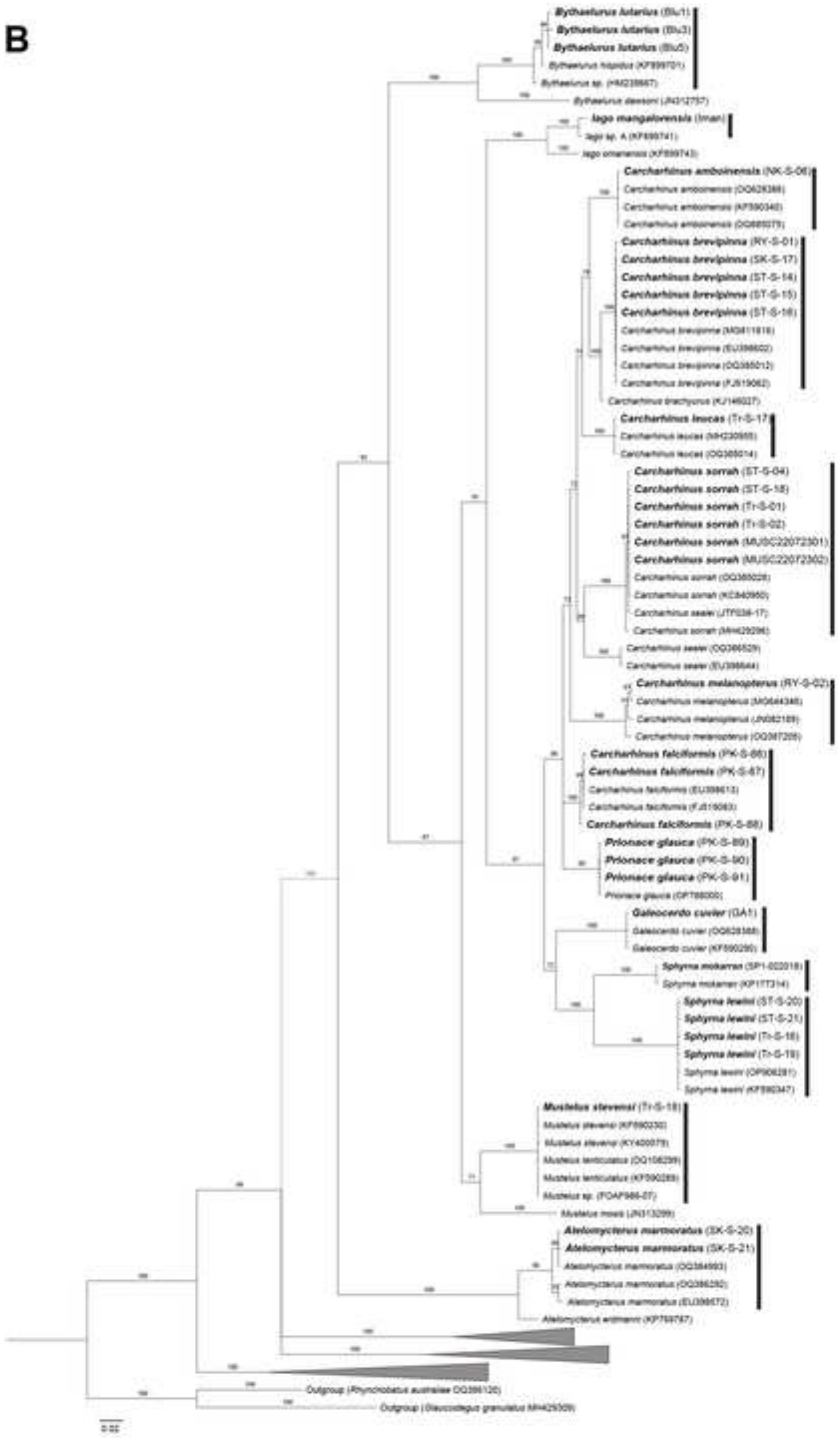

Fig 2A

Click here to access/download;Figure;fig2_sharks_ND2_A_edit.jpg

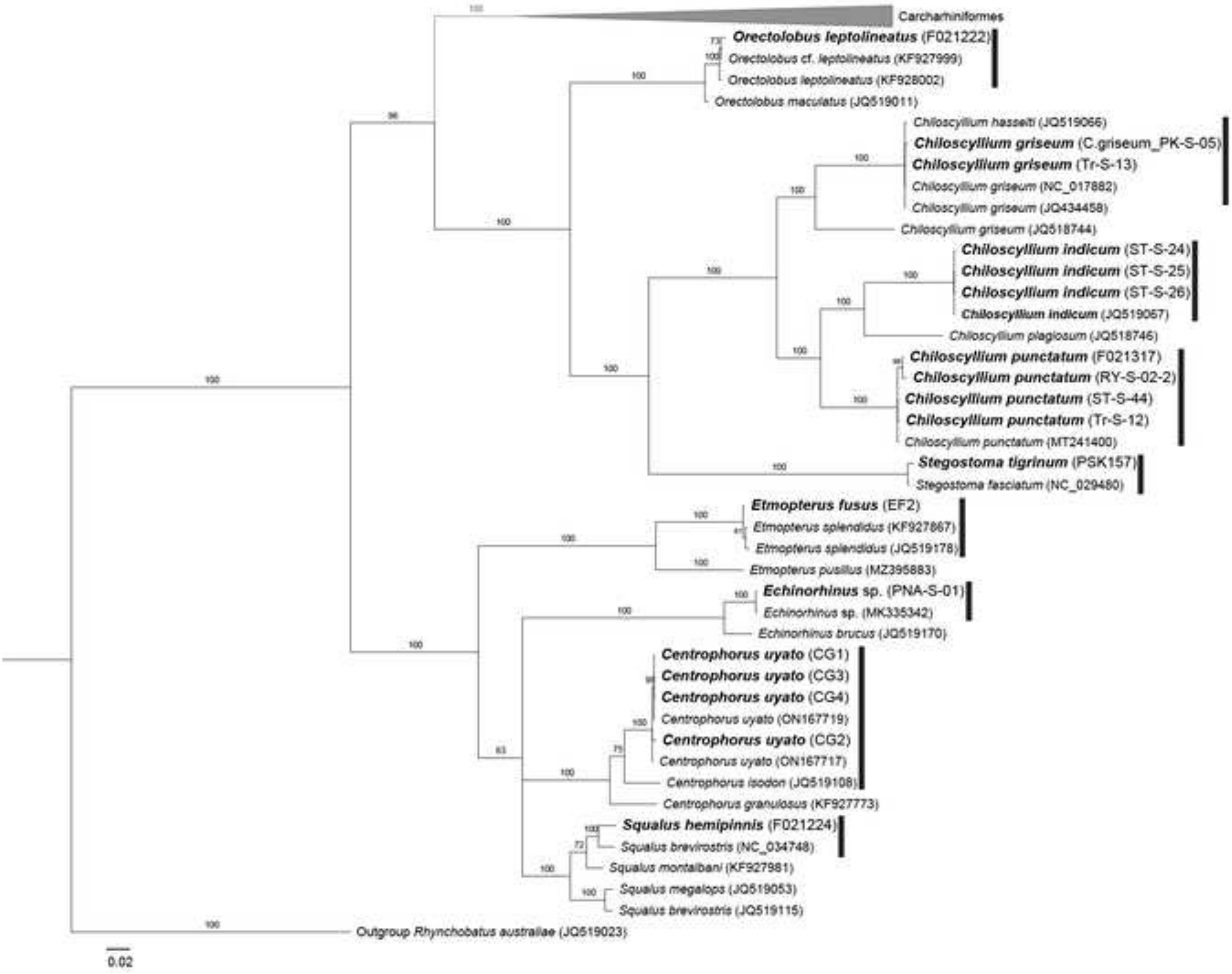

Fig 2B

Click here to access/download;Figure;fig2_sharks_ND2_B_edit.jpg

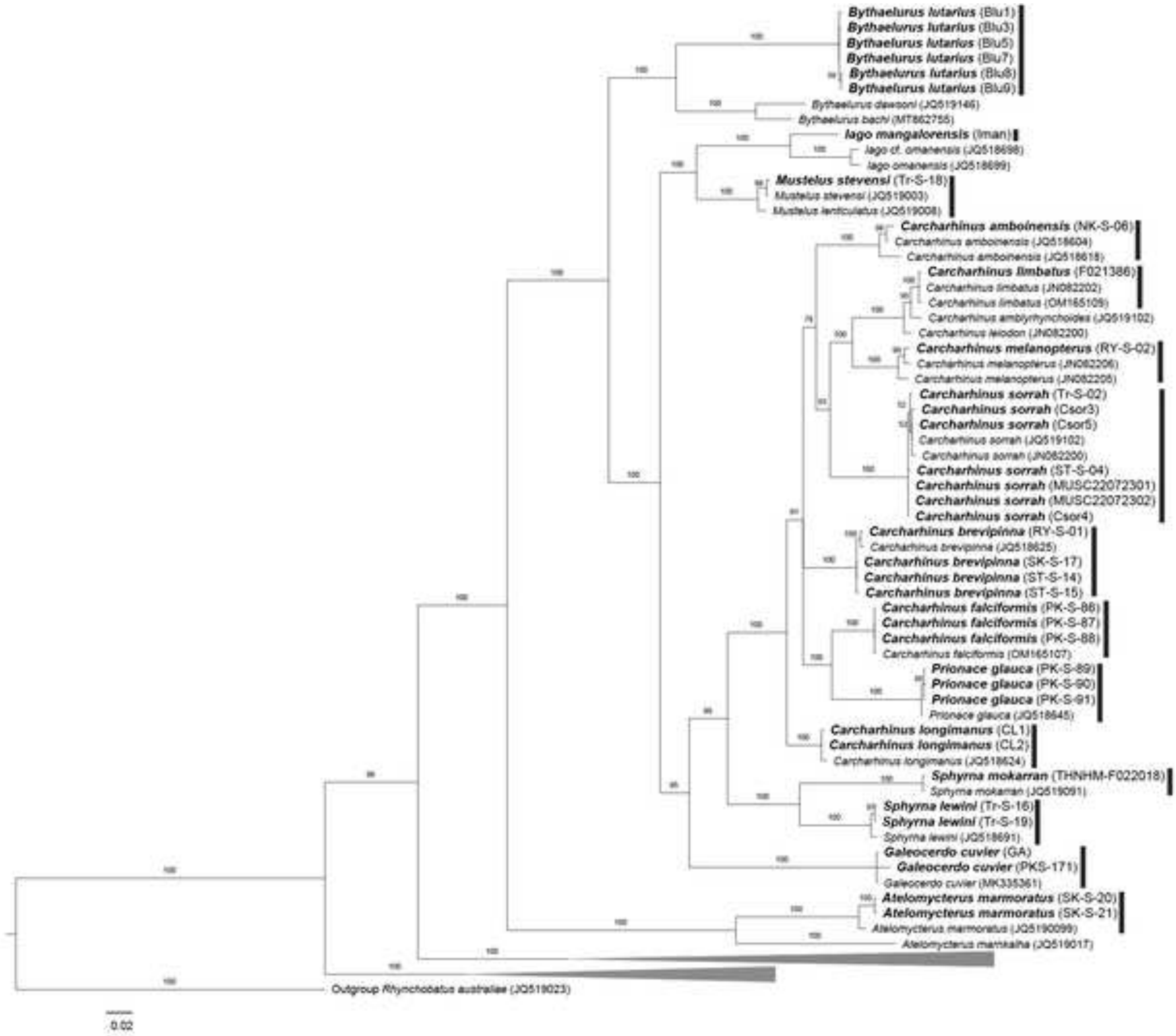

Fig 3A

Click here to access/download;Figure;fig3_Batoids_COI_A_edit.jpg

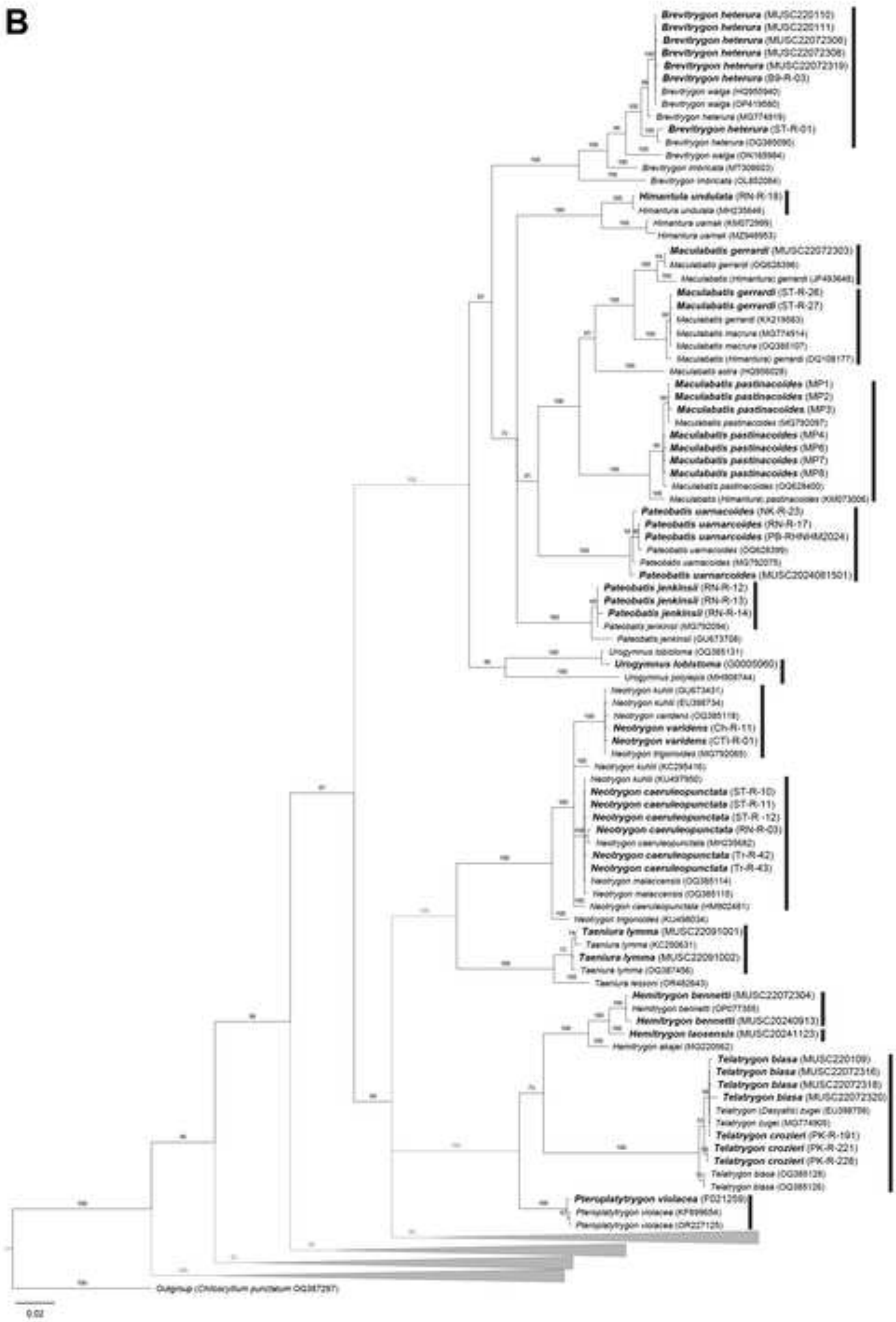

Fig 3B

Click here to access/download;Figure;fig3_Batoids_COI_B_edit.jpg

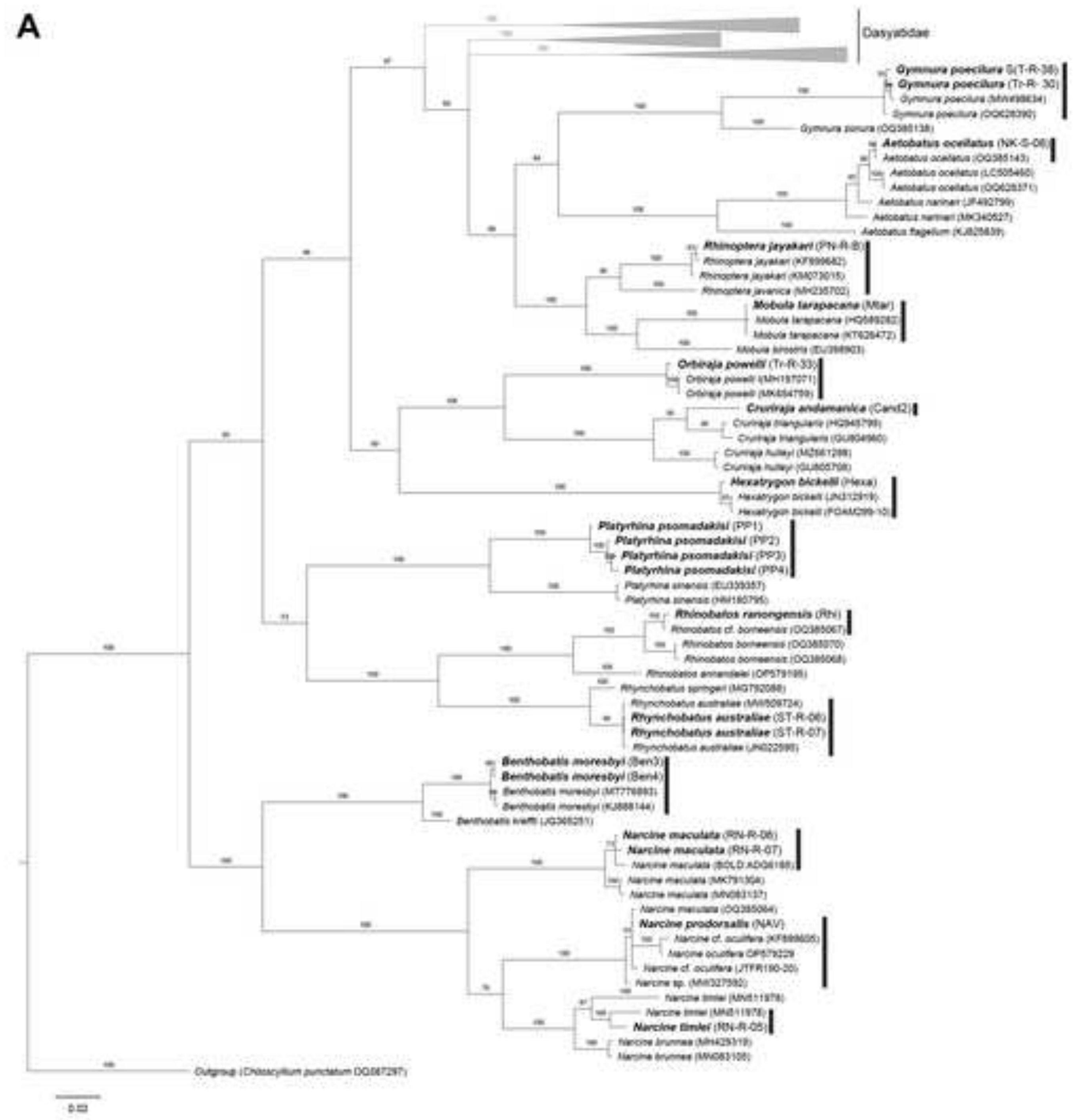

Fig 3C

Click here to access/download;Figure;fig3_batoids_COI_C_edit.jpg

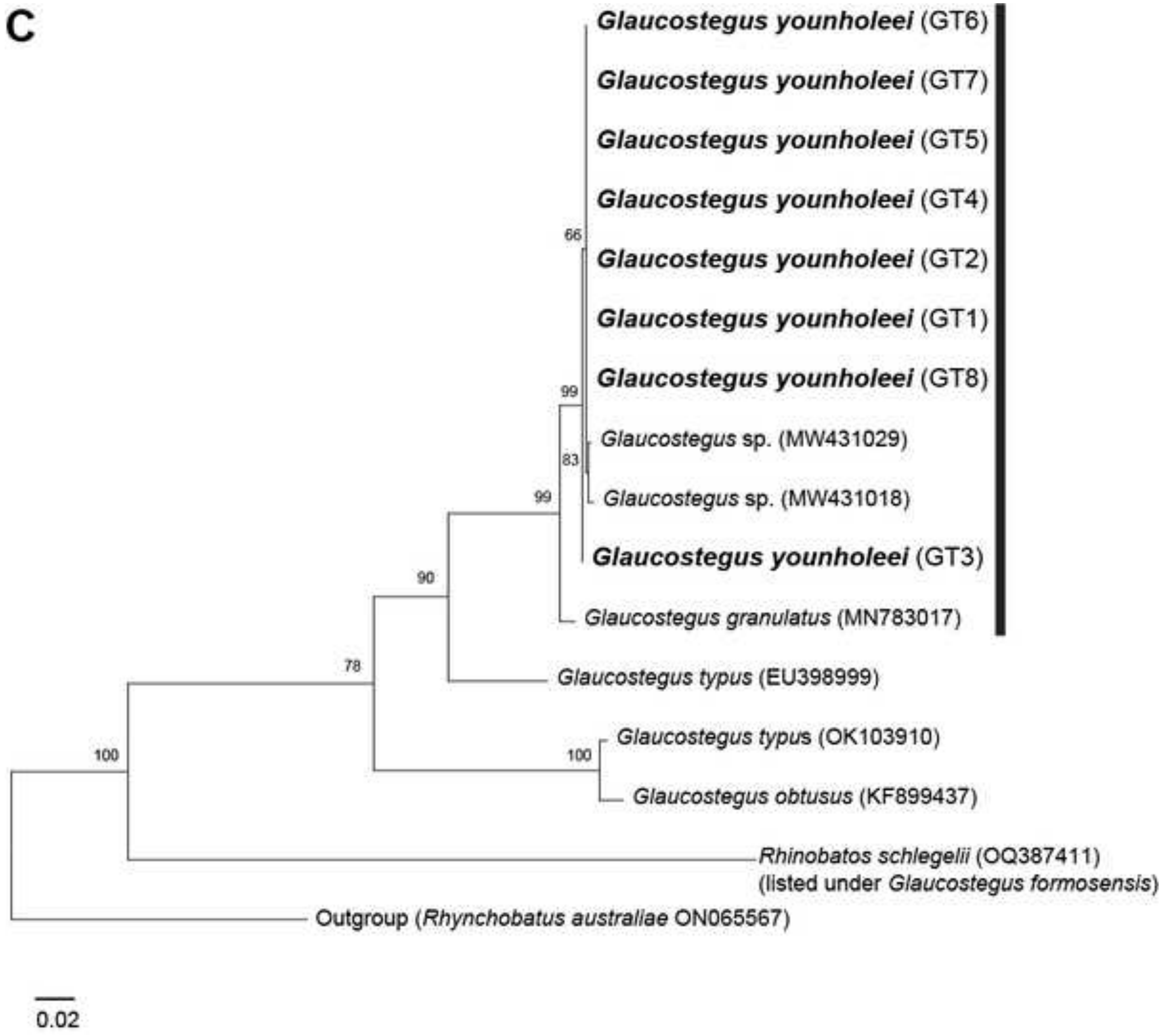

Fig 4A

Click here to access/download;Figure;fig4_Batoids_ND2_A_edit.jpg

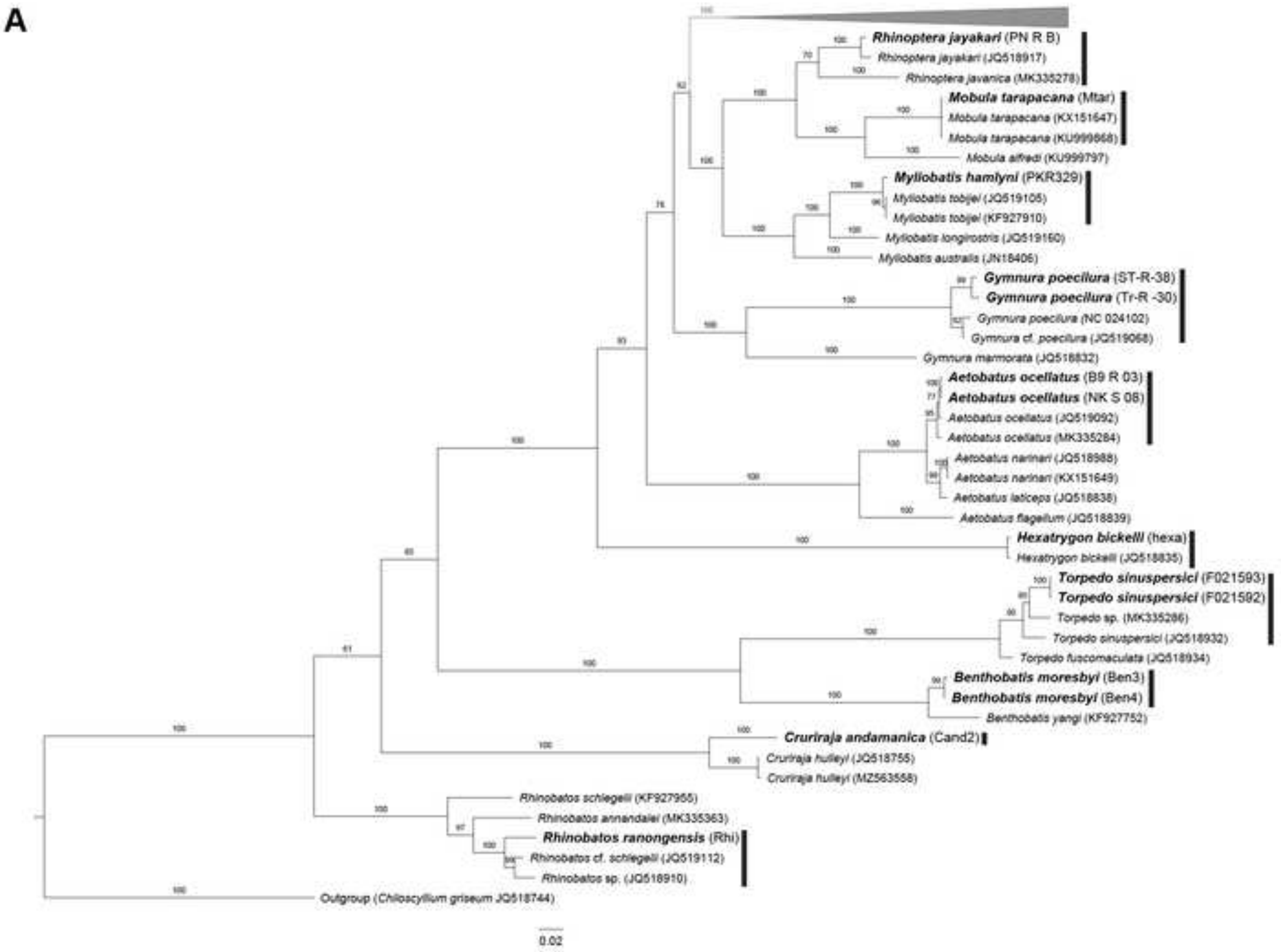

Fig 4B

Click here to access/download;Figure;fig4_Batoids_ND2_B_edit.jpg

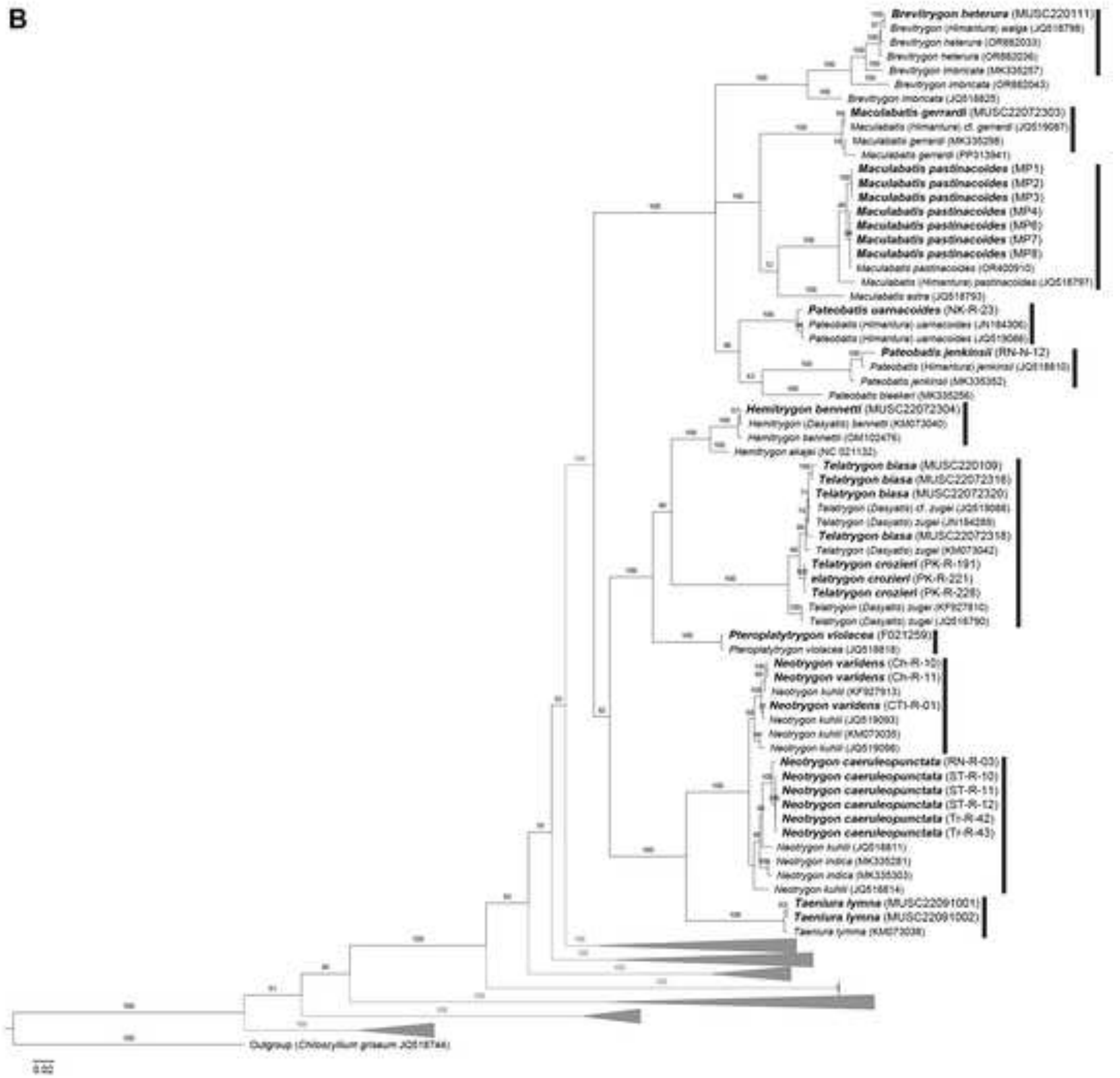

Fig 5

Click here to access/download;Figure;fig5_K2P_shark.tif

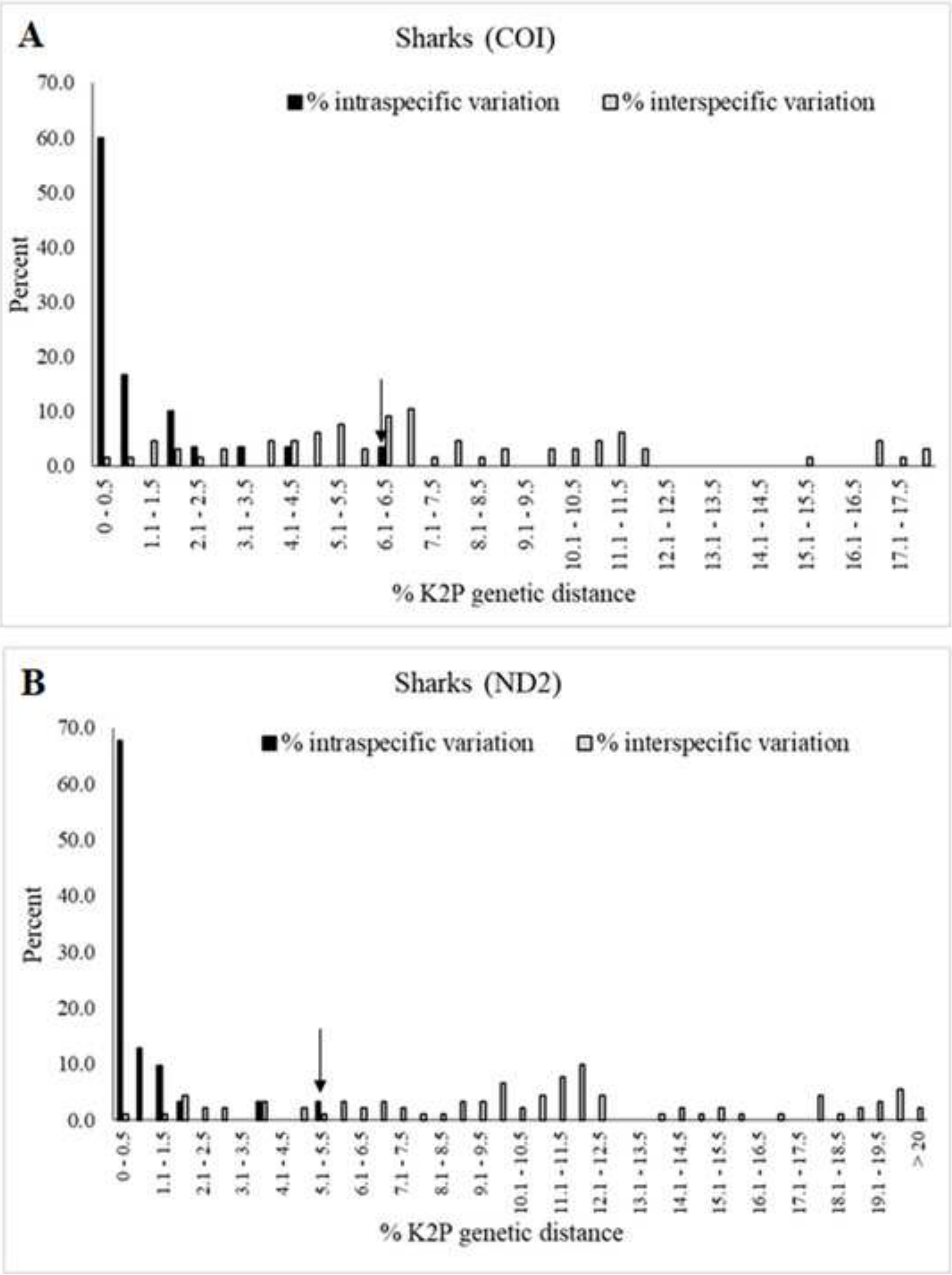

Click here to access/download;Figure;fig6_K2P_batoid.tif

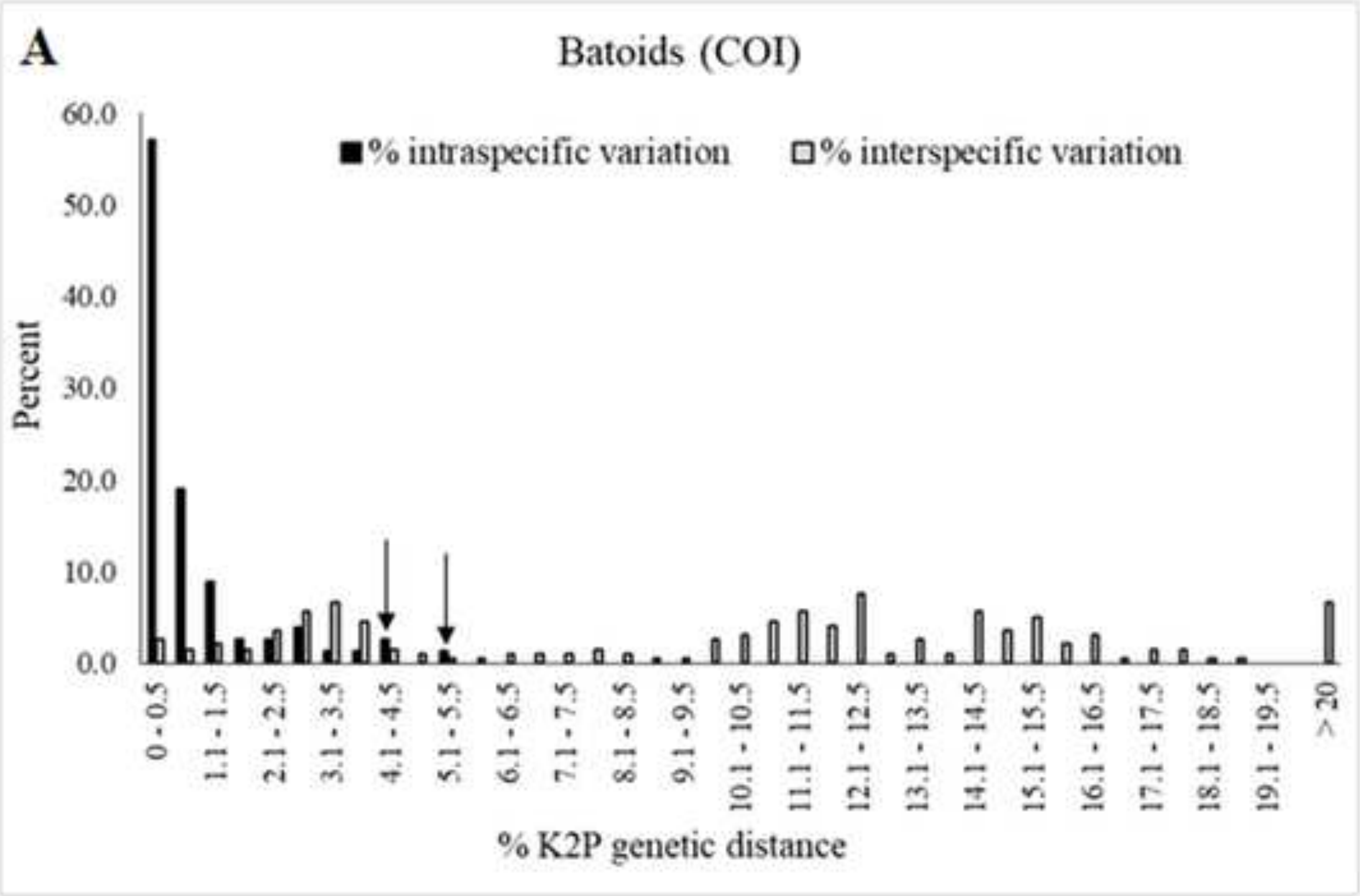

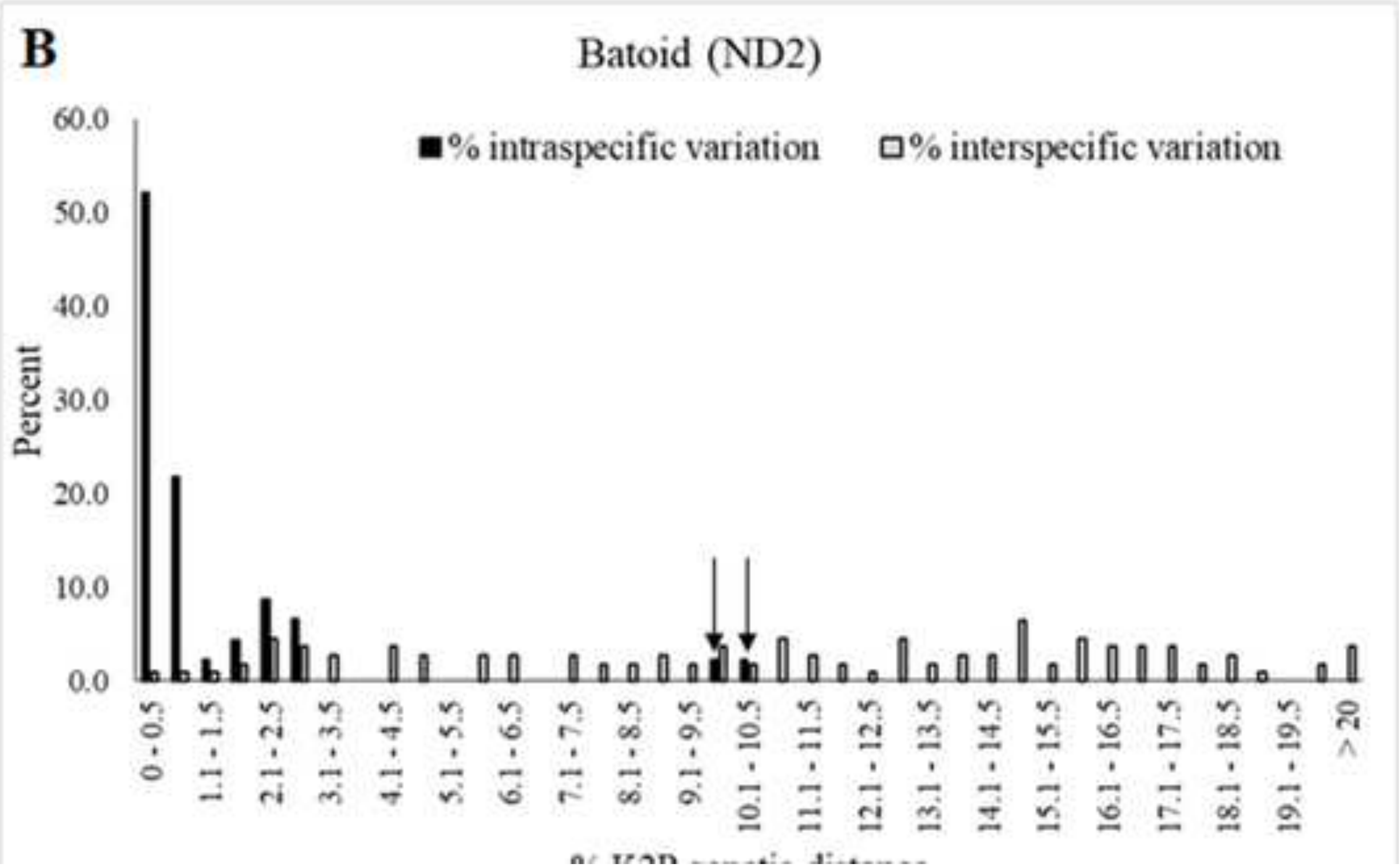

Click here to access/download
Supporting Information
supporting_info_S1.docx

Click here to access/download
Supporting Information
supporting_info_S2.docx

DNA barcoding for elasmobranch diversity assessment in Thailand: its advantages and limitations

Jenjit Khudamrongsawat[1], Tassapon Krajangdara[2], Thadsin Panithanarak[3], Ratima Karuwancharoen[3], Wanlada Klangnurak[4], Pattarapon Promnun[5], Wansuk Senanan[6**]

[1]Department of Biology, Faculty of Science, Mahidol University, Rama VI Road, Rajathewi District, Bangkok, 10400, Thailand
[2]66/27 Soi 5, Thep Thani Village, Moo 2, Rasada Subdistrict, Muang District, Phuket Province 83000, Thailand
[3]Institute of Marine Science, Burapha University, 169 Long Had Bangsaen Rd, Saen Suk, Chon Buri District, Chon Buri 20131, Thailand
[4]Department of Animal Production Technology and Fishery, School of Agricultural Technology, King Mongkut's Institute of Technology Ladkrabang, Bangkok, 10520, Thailand
[5]Geneus Genetics Co.,Ltd, 37/1 Sukhumvit 101/1, Bang Chak, Phra Kanong, Bangkok, 10260, Thailand
[6]Department of Aquatic Science, Burapha University, 169 Long Had Bangsaen Rd, Saen Suk, Chon Buri District, Chon Buri 20131, Thailand

******corresponding author

Abstract

The assessment of elasmobranch biodiversity in Thailand benefits greatly from the application of DNA barcoding, which helps mitigate the challenge posed by a shortage of expert taxonomists. Fragments of COI and ND2 mitochondrial DNA were examined, and the strength and weaknesses of these two markers were compared. In this study, DNA products from 153 elasmobranch samples were amplifiable and revealed a total of 28 shark species and 32 batoid species ~~were identified~~. Many species could be confidently identified as their morphological characteristics aligned with DNA barcodes. However, several exceptions were recognized. The absence of reference sequences for rare species presented a challenge for species verification, and the misidentification of reference sequences, as well as changes in species names due to taxonomic revisions, added complexity when comparing DNA barcoding sequences. Conflicts between morphology and genetics were also observed. While intraspecific genetic variation based on both DNA barcodes generally indicated 0-2% variation, this metric could not always be used for species delimitation. This was particularly true for species displaying low genetic variation among closely related species and species where cryptic diversity remained hidden and yet to be uncovered. In such cases, the morphological characteristics of the samples served as a primary means ~~for~~ of species identification. Despite these challenges, DNA barcoding remains an invaluable tool for biodiversity assessment, especially in light of the shortage of skilled experts and identification of products made of vulnerable species. However, it is essential to exercise caution~~s~~ and be aware of these complexities in its application.

Keywords: sharks, rays, batoid, COI, ND2, genetic variation, conservation

Introduction

The Southeast Asian region (SEA) boasts a remarkable array of elasmobranch species in which the ongoing discovery of new, undescribed species is adding to our appreciation of the extraordinary diversity of this under-studied group [1, 2]. To safeguard these taxa in the region, the Southeast Asian Fisheries Development Center (SEAFDEC) has been actively encouraging SEA countries to assess their elasmobranch diversity so as to better formulate international conservation policies. Among SEA nations, Thailand is one of chondrichthyan biodiversity hotspots. This is attributable to the country's expansive marine ecosystem covering nearly 3,000 kilometers of coastlines, spanning both the Indo-West Pacific region and the Indian Ocean. The geological dynamics and intricate evolutionary history further contribute to this biodiversity wealth [3, 4]. The latest records unveil over 180 species (across 12 orders and 43 families), including some apparently distinct, yet morphologically similar lineages are yet to be confirmed as full species [5]. Evident disparities exist in elasmobranch diversity between the Gulf of Thailand and the Andaman Sea. The species composition within the Gulf of Thailand tends to closely resemble that of the western Pacific region, whereas the Andaman Sea shows resemblances to species prevalent in the east Indian Ocean. Notably, the Andaman Sea boasts a higher number of elasmobranch species compared to the Gulf of Thailand. This discrepancy is further underscored by the presence of Holocephali species exclusively recorded in the Andaman Sea [5, 6]. The escalating species count is a result of enhanced survey efforts and the integration of molecular tools for species identification. Despite ongoing surveys and the submission of DNA barcode information to the NCBI database and Barcode of Life Database (BOLD), Thailand's remarkable elasmobranch diversity remains poorly in the existing literature [7–10]. Available genetic information, though scant, poses many questions regarding taxonomic diversity in which the high levels of genetic differentiation that could not be ignored [11].

The evaluation of elasmobranch diversity hinges on the utilization of both morphological and molecular tools, with DNA barcoding especially the cytochrome c oxidase subunit I (COI) and the nicotinamide adenine dehydrogenase 2 (ND2) gene fragments, gaining prominence [7, 12–15]. DNA barcoding facilitates the differentiation of cryptic species that are difficult to distinguish based on appearance alone [16]. Even species with extensive and apparently continuous distributions may exhibit genetic variations that have implications for disparate evolutionary trajectories [17, 18]. Specifically, ND2 has shown superiority in uncovering genetic variations in cryptic sharks and is strongly recommended for use in chondrichthyan DNA barcoding [7, 19]. While DNA barcoding offers a powerful and expeditious tool for assessing biodiversity, it has proven challenging to establish clear criteria for genetic differentiation between species, particularly for non-taxonomists. In many instances, genetic variations within elasmobranchs fall within a 2% range at the intraspecific level [13, 15, 20–22]. However, exceptions do exist [13, 15, 19, 23, 24]. These strengths and weaknesses of DNA barcoding for biodiversity assessment in various taxa have also been acknowledged [25–33]. Nonetheless, as Thailand has an urgent need for improved the country's biodiversity assessment, the adoption of

DNA barcoding is a worthwhile endeavor. Genetic insights into species have yielded significant advances in shaping the Thailand National Plan of Action for the Conservation and Management of Sharks (NPOA-Sharks, Thailand). This aligns with the nation's economic and development agenda in accordance with the United Nations' 2030 Sustainable Development Goals (SDGs).

This research's primary objectives were to evaluate the extent of DNA barcoding application based on COI and ND2 fragments for both taxonomists and non-taxonomists in elasmobranch biodiversity assessment and to create DNA barcoding databases of elasmobranch diversity in Thailand. The outcomes hold the potential to provide valuable contributions to the regional conservation efforts by shedding light on Thailand's unique biological resources.

Methods

- **Tissue sampling locations and collections**

Permission for animal use was granted by MU-IACUC (permission MUSC62-025-489) and Burapha U-ethics (IACUC 002/2566). Samples were obtained from various sources, encompassing research vessels, fish markets, and fishing harbors situated in both the Gulf of Thailand and the Andaman Sea over the period from 2013 to 2023. These specimens were photographed and underwent subsequent species identification by T. Krajangdara. The identification process relied on following the guidance provided in Krajangdara et al. [5], Last et al. [34], Krajangdara [35], and Ebert et al. [36]. Instances where characteristics proved intricate or displayed unclear attributes were categorized as unidentified species, designated with "cf." or "sp." To definitively classify these cases, molecular analysis was performed. Muscle or fin clips were excised from the specimens and conserved in 95% ethanol, subsequently being frozen at -20°C at Burapha University, Mahidol University, King Mongkut's Institute of Technology Ladkrabang, and the Natural History Museum of Thailand. In cases involving sizeable, expensive, or live individuals, photographs were taken as records, with the focus being solely on acquiring tissue samples. To ensure the future viability of the research, selected samples were designated as voucher specimens and retained at these institutions: the Phuket Marine Biological Center, Burapha University, King Mongkut's Institute of Technology Ladkrabang, and Natural History Museum of Thailand.

- **Molecular examination**

DNA samples were extracted from more than 200 samples using commercial Extraction Kit (QIAGEN━DNeasy kit). The quality of the extracted DNA samples was assessed by electrophoresis on a 1% agarose gel. Their concentrations were measured using a Nanodrop (NanoVue^{TM}). DNA barcoding based on cytochrome c oxidase subunit I (COI) and NADH dehydrogenase subunit 2 (ND2) were amplified following the protocols by Ward et al. [12] and Naylor et al. [7]. Degraded tissue samples, especially those obtained before 2020, could not be amplified using universal primers, and so new primers were designed for investigation. List of primers used in this study were listed in Supporting information (S1 File). Polymerase chain reactions (PCR) were carried out with a reaction total volume of 25 ul using 5-7 ng of DNA template, 2 µl (0.2 mM) dNTP, 2.5 µl 10xPCR buffer, 1 µl (0.5 µM) for each primer, 1 µl (2 mM) $MgCl_2$, 0.05 µl (5 unit/µl) Taq DNA polymerase, and deionized water. PCR cycles were performed with an initial denaturation at 94°C for 3 min, followed by 35 cycles of 94°C for 50 s,

Formatted: Font: Bold

Formatted: Font: Bold

Formatted: Superscript

annealing at 50°C for 2 min, and extension at 72°C for 90 s, and a final extension at 72°C for 6 min. For samples posing difficulty in amplification, a touchdown PCR profile was performed including an initial denaturation at 94°C for 3 min, followed by 5 cycles of 94°C for 30 s, annealing at 48°C for 60 s, and extension at 72°C for 90 s, and then followed by 30 cycles of 94°C for 30 s, annealing at 50°C for 60 s, and extension at 72°C for 90 s, and a final extension at 72°C for 10 min. PCR products were checked by electrophoresis using 1.5% agarose gel and purified using commercial Gel/PCR purification kit (QIAGEN━QIAquick PCR purification kit). Sequences were sent for Sanger sequencing using an ABI 3730 automated sequencer. All sequences were edited, translated into amino acid using MEGA 11 [37].

- **Data analyses**

Formatted: Font: Bold

Species were confirmed through a dual process involving both morphological identification and cross-referencing with reference sequences available in the National Center for Biotechnology Information (NCBI) and the Barcode of Life Database (BOLD system). Taxonomic name changes, particularly in the family Dasyatidae, were also recognized [38]. The differences in the generic names of species in the genera previously named as *Himantura* and *Dasyatis*, were not treated as different from the newly assigned names. In instances where matches were inconclusive, a thorough assessment was conducted using supplementary information, notably morphology and geographic data. In cases of persistent uncertainty, the designation "cf." was attributed to those particular samples. The validation of species through molecular means relied on a similarity scoring system, demanding a minimum of 95% query coverage and a similarity level of 98% or higher. The 95% query coverage threshold was selected for its robustness and greater likelihood of yielding accurate matches. A similarity level of 98% or higher was chosen because intraspecific genetic variation in most fish species studied to date typically falls within this range [13, 15, 20–22]. This methodology utilized the BLAST algorithm within (NCBI) and BOLD Identification Engine., as well as the Identification Engine embedded in BOLDSYSTEMS. Nevertheless, COI fragments from our samples did not overlap with most *Glaucostegus* COI sequences in the public database due to very poor DNA quality resulting in sequences that did not align well with published reference sequences, preventing the application of a 95% query coverage threshold. Instead, a minimum coverage of 60% or higher was used to achieve the best possible results. A separate file was created for *Glaucostegus* tree construction. Following these comprehensive validations, the genetic sequences were duly submitted to the NCBI database, contributing to the broader body of scientific knowledge and facilitating further research and insights into the studied species (S2 file).

~~Furthermore, to elucidate the relationships between the samples under investigation and the reference data, phylogenetic trees were constructed.~~ To assess relationships between the samples and reference data, phylogenetic trees were constructed. Sequences were aligned in MEGA 11 and saved as FASTA files. For COI analysis in sharks, sequences of *Rhynchobatus australiae* and *Glaucostegus granulatus* served as outgroups, while that of *R. australiae* was used for ND2. In batoids, sequences of *Chiloscyllium punctatum* and *C. griseum* were used as outgroups for COI and ND2, respectively. The ~~best-fit substitution~~ model for the sequence alignment was selected using IQ-TREE. ModelFinder [39]. This method, integrated within IQ-TREE V2.3.6, was employed to identify the best substitution model based on the Bayesian Information Criterion (BIC), which was TIM2+F+I+G4. Bayesian inference of phylogeny was

Formatted: Font: Italic

Formatted: Font: Italic

Formatted: Font: Italic

Formatted: Font: Italic

Formatted: Font: Italic

conducted using MrBayes (v3.2.7) [40]. A Markov Chain Monte Carlo (MCMC) analysis was executed in MrBayes with a total of 1,000,000 generations. The posterior distribution was sampled every 100 generations. Four chains were used to improve exploration of the posterior distribution. After the analysis, 25% of the samples were discarded as burn-in to eliminate the influence of the starting state. Convergence diagnostics, including the standard deviation of split frequencies and effective sample sizes (ESS), were assessed to ensure reliability of the analysis. The consensus tree was generated ~~and visualized~~ by Figtree V1.4.4 with posterior probabilities representing the clade support [41].

These trees offered a visual representation of the evolutionary placements for both sharks and batoids. The genetic distances were quantified using the Kimura-2-Parameter (K2P) method, gauging the variations of intra- and inter- specific diversity of species and genera of samples in this study. For this analysis, the reference sequences of unidentified species of reference sequences were excluded. Because several species in this study were represented by a small sample size, sequences in NCBI and BOLD databases under the same scientific names were used to enable intraspecific comparison.

Results

There were 153 samples with successful amplification of either COI or ND2 fragments or both. The two DNA barcoding fragments offered different advantages (Table 1). COI fragments showed better rate of amplification success than ND2, while ND2 provided more characteristics for comparison. A larger number of ND2 sequences could not be matched with any references in the database, whereas most COI sequences found their matches, but some matched sequences attributed to more than one species and were therefore inconclusive. Nevertheless, the matching results did not indicate correct species identification because conclusive species identification was conducted using morphological examination. The genetic data only showed possible identification if only DNA sequences were available.

Table 1. Comparisons of COI and ND2 fragments as DNA barcoding for elasmobranch species identification from 153 samples.

criteria	COI	ND2
number of sequences of successful amplification	130	99
length of fragments obtained (bp)	520-676	970-1047
number of sequences NOT matched with any reference sequences or matched unidentified species	9	31
number of sequences matched with multiple species	26	0

With the combined use of morphological and genetic data, the samples in this study unveiled a total of 28 shark species and 32 batoid species. The process of arriving at consensus species heavily relied on morphological traits, with genetic data serving a supplementary role. Identification to species was congruent when assessed through both morphological and genetic examination when allowing for intraspecific genetic variations of no more than 2% (Table 2; Figs 1– 4). Phylogenetic analysis of a sample assigned to *Orectolobus leptolineatus* on morphological criteria matched an unidentified *Orectolobus* sequence (Fig 2A).

Figure 1. Phylogenetic tree of sharks based on 654 bp of COI fragments; (A) overall relationships of sharks in this study (B) phylogenetic tree of sharks in the order Carcharhiniformes; bold = samples in this study; number on lines indicating bootstrap values.

Figure 2. Phylogenetic tree of sharks based on 1044 bp of ND2 fragments; (A) overall relationships of sharks in this study (B) phylogenetic tree of sharks in the order Carcharhinoformes; bold = samples in this study; number on lines indicating bootstrap values.

Figure 3. Phylogenetic tree of batoids based on 652 bp of COI fragments; (A) overall relationships of batoids in this study (B) phylogenetic tree of batoids in the family Dasyatidae; (C) phylogenetic tree of batoids in the genus *Glaucostegus*, bold = samples in this study, sequences MW431029, MW431018 identified as *G. younholeei* (Habib and Islam 2021).; number on lines indicating bootstrap values.

Figure 4. Phylogenetic tree of batoids based on 1044 bp of ND2 fragments; (A) overall relationships of batoids in this study (B) phylogenetic tree of sharks in the family Dasyatidae; bold = samples in this study; number on lines indicating bootstrap values.

Table 2. Species identification based on morphological and genetic characteristics.

Morphological identification	genetic identification based on COI and/or ND2 (best match)	Phylogenetic tree placement	consensus species	problem
**SHARKS**				
ECHINORHINIFORMES				
Echinorhinus brucus	*Echinorhinus* sp.	*Echinorhinus* sp.	*E. brucus*	Insufficient information for comparison
SQUALIFORMES				
Squalus hemipinnis	not matched	*S. brevirostris*	*S. hemipinnis*[*1]	Insufficient information for comparison
Squalus montalbani	*Squalus* sp.	*Squalus* sp. A	*S. montalbani*	Insufficient information for comparison
Centrophorus uyato	*C. granulosus/ C. zeehaani/ C. uyato*	*Cen. uyato*	*C. uyato*[*2]	Taxonomic revision
Etmopterus fusus	not matched	Not group with any species (COI); *E. splendidus* (ND2)	*E. fusus*	Discordance between morphology and genetics
ORECTOLOBIFORMES				
Orectolobus leptolineatus	*O.* cf. *leptolineatus*	*O. leptolineatus*	*O. leptolineatus*	None
Chiloscyllium griseum	*C. griseum*	*C. hasselti/ C. griseum*	*C. griseum*	None
Chiloscyllium hasselti	*C. hasselti/ C. griseum*	*C. hasselti/ C. griseum*	*C. hasselti*	Cryptic morphological similarity
Chiloscyllium indicum	*C. indicum*	*C. indicum*	*C. indicum*	None
Chiloscyllium punctatum	*C. punctatum*	*C. punctatum*	*C. punctatum*	None
Stegostoma tigrinum	*S. fasciatum*	*S. fasciatum*	*S. tigrinum*[*3]	None
LAMNIFORMES				
Isurus oxyrinchus	*I. oxyrinchus*	*I. oxyrinchus*	*I. oxyrinchus*	None
C~~H~~ARCHARHINIFORMES				
Atelomycterus marmoratus	*A. marmoratus*	*A. marmoratus*	*A. marmoratus*	None
Bythaelurus lutarius	*B. hispidus*	*B. hispidus* (COI); not grouped with any species (ND2)	*B.* cf. *lutarius*	Discordance between morphology and genetics
Iago mangalorensis	*Iago* sp.	*Iago* sp. A	*I.* ~~*mangalorensis*~~ *gopalakrishnani*[*4]	~~Insufficient information for comparison~~ Taxonomic revision
Mustelus stevensi	*Mustelus* sp./ *M. lenticulatus/ M. stevensi*	*M. lenticulatus* (COI); *M. stevensi* (ND2)	*M. stevensi*	Cryptic morphological similarity
Carcharhinus amboinensis	*C. amboinensis*	*C. amboinensis*	*C. amboinensis*	None
Carcharhinus brevipinna	*C. brevipinna*	*C. brevipinna*	*C. brevipinna*	None
Carcharhinus falciformis	*C. falciformis/ C. brevipinna*	*C. falciformis*	*C. falciformis*	Cryptic morphological similarity
Carcharhinus leucas	*C. leucas*	*C. leucas*	*C. leucas*	None

Formatted: Superscript

Morphological identification	genetic identification based on COI and/or ND2 (best match)	Phylogenetic tree placement	consensus species	problem
Carcharhinus limbatus	*C. limbatus*	*C. limbatus*	*C. limbatus*	None
Carcharhinus longimanus	*C. longimanus*	*C. longimanus*	*C. longimanus*	None
Carcharhinus melanopterus	*C. melanopterus*	*C. melanopterus*	*C. melanopterus*	None
Carcharhinus sorrah	*C. sorrah/ C. sealei*	*C. sorrah*	*C. sorrah*	Cryptic morphological similarity
Prionace glauca	*P. glauca*	*P. glauca*	*P. glauca*	None
Galeocerdo cuvier	*G. cuvier*	*G. cuvier*	*G. cuvier*	None
Sphyrna lewini	*S. lewini*	*S. lewini*	*S. lewini*	None
Sphyrna mokarran	*S. mokarran*	*S. mokarran*	*S. mokarran*	None
**BATOIDS**				
RHINOPRISTIFORMES				
Rhynchobatus australiae	*R. australiae*	*R. australiae*	*R. australiae*	None
Rhinobatos ranongensis	no matched	*Rhinobatos* cf. *borneensis*	*R. ranongensis*	Insufficient information for comparison
Glaucostegus younholeei	*Glaucostegus* sp. (later as *G. younholeei*)/ *G. granulosus*	*Glaucostegus* sp./ *G. granulosus*	*G. younholeei*[*45]	Cryptic morphological similarity
Platyrhina psomadakisi	not matched	not grouped with any species	*P. psomadakisi*	Insufficient information for comparison
TORPEDINIFORMES				
Torpedo sinuspersici	not matched	*Torpedo* sp./ *T. sinuspersici*	*T. sinuspersici*	Insufficient information for comparison
Benthobatis moresbyi	*B. moresbyi*	*B. moresbyi* (COI); not grouped with any species (ND2)	*B. moresbyi*	None
Narcine maculata	*N. maculata*	*N. maculata*	*N. maculata*	None
Narcine prodorsalis	*N. maculata/ Narcine* sp./ *N.* cf. *oculifer*	*Narcine* sp.	*N. prodorsalis*	Cryptic morphological similarity
Narcine timlei	*N. timlei/ N. brunnea*	*N. timlei*	*N. timlei*	Cryptic morphological similarity
RAJIFORMES				
Orbiraja powelli	*O. powelli*	*O. powelli*	*O. powelli*	None
Cruriraja andamanica	not matched	*Cruriraja triangularis* (COI); *C. hulleyi* (ND2)	*C. andamanica*	Insufficient information for comparison
MYLIOBATIFORMES				
Hexatrygon bickelli	*H. bickelli*	*H. bickelli*	*H. bickelli*	None
Gymnura poecilura	*G. poecilura*	*G. poecilura*	*G. poecilura*	None

Morphological identification	genetic identification based on COI and/or ND2 (best match)	Phylogenetic tree placement	consensus species	problem
Brevitrygon heterura	*B.* (*Himantura*) *walga*/ *B. heterura*	*B. walga* / *B. heterura*	*B. heterura*[*65]	Taxonomic revision
Hemitrygon bennetti	*H.* (*Dasyatis*) *bennetti*	*H. bennetti*	*H. bennetti*	None
Hemitrygon laosensis	not matched	not grouped with any species	*H. laosensis*	Insufficient information for comparison
Himantula undulata	*H. undulata*	*H. undulata*	*H. undulata*	None
Maculabatis gerrardi	*M. gerrardi*/ *M. macrura*	*M. macrura*/ *M. gerrardi*	*M. gerrardi*	Cryptic morphological similarity
Maculabatis pastinacoides	*M. pastinacoides*	*M. pastinacoides*	*M. pastinacoides*	None
Neotrygon caeruleopunctata	*N. kuhlii*/ *N. malaccensis*/ *N. caeruleopunctata*	*N. kuhlii* / *N. varidens*/ *N. caeruleounctata*	*Neotrygon caeruleopunctata* [*76]	Ttaxonomic revision
Neotrygon varidens	*N. trigonoides*/ *N. kuhlii*/ *N.* cf. *kuhlii*	*N. kuhlii*/ *N. trigonoides*	*N. varidens*[*76]	Taxonomic revision
Pateobatis jenkinsii	*P.* (*Himantura*) *jenkinsii*	*P. jenkinsii*	*P. jenkinsii*	None
Pateobatis uarnacoides	*P.* (*Himantura*) *uarnacoides*	*P. uarnacoides*	*P. uarnacoides*	None
Pteroplatytrygon violacea	*P. violacea*	*P. violacea*	*P. violacea*	None
Taeniura lymma	*T. lymma*	*T. lymma*	*T. lymma*	None
Telatrygon biasa	*T.* (*Dasyatis*) *zugei*/ *T. biasa*	*T.* (*Dasyatis*) *zugei* (COI); *T.* (*Dasyatis*) *zugei* / *T. biasa* (ND2)	*T. biasa*[*8]	Taxonomic revision
Telatrygon crozieri	*T.* (*Dasyatis*) *zugei*	*T.* (*Dasyatis*) *zugei* / *T. biasa*	*T. crozieri*[*87]	Taxonomic revision
Urogymnus lobistoma	*U. lobistoma*	*U. lobistoma*	*U. lobistoma*	None
Myliobatis hamlyni	*M. tobijei*	*M. tobijei*	*M. hamlyni*[*98]	Taxonomic revision
Aetobatus ocellatus	*A. ocellatus*/ *A. narinari*	*A. ocellatus*	*A. ocellatus*[*109]	Taxonomic revision
Rhinoptera jayakari	*R. jayakari*	*R. jayakari*	*R. jayakari*	None
Mobula tarapacana	*M. tarapacana*	*M. tarapacana*	*M. tarapacana*	None

*1 short ND2 fragment comparison = *S. hemipinnis*

*2 taxonomic revision—*C. zeehaani* = syn. of *C. uyato* often misidentified with *C. granulosus* [42]

*3 lListed as *Stegostoma fasciatum* in BOLD, listed as *S. fasciatum* and *S. tigrinum* in NCBI

*4 *Glaucostegus* sp. in GenBank described as *G. younholeei* sp. nov. in Habib and Islam [43] taxonomic revision and species resurrection [43]

*5 *Glaucostegus* sp. in GenBank described as *G. younholeei* sp. nov. in Habib and Islam [443]

*65 taxonomic revision and geographic distribution of *Brevitrygon* [38, 45]

*76 taxonomic revision of *Neotrygon kuhlii* [44-4546-48]

*87 taxonomic revision and new species erectionspecies resurrection [34]

*98 taxonomic revision and new species erection species resurrection [4649]

*109 taxonomic revision and new species erection species resurrection [4750]

Not all samples could be identified. Problems regarding species identification could be grouped into 4 categories.

1. Insufficient information for comparison/ lack of species registered in the database

Specific challenges emerged in cases where information on references was unavailable within established databases. In these instances, the primary criterion for species identification was based on morphological characteristics of the samples. These samples included rarely found species such as *Hemitrygon laosensis*, *Echinorhinus brucus*, *Squalus montalbani*, ~~*Iago mangalorensis*, ~~*Platyrhina psomadakisi*, *Torpedo sinuspersici*, and *Cruriraja andamanica*. Genetic data for some species ~~are newly described ~~such as *Rhinobatos ranongensis* were not available for public access. In these cases, we relied solely on morphology for species identification.

For *Squalus hemipinnis*, the reference sequences of which did not fully meet the sequence alignment criteria of this study, and did not align with any known species. Although the phylogenetic tree placed it in close proximity to *S. brevirostris*, there was a 2.5% genetic difference. An alignment of short fragments of ND2 of our specimen and sequences in the NCBI database (529 base pairs—not shown) listed under *S. hemipinnis* revealed their similarity. This information was omitted and not presented here. By combining morphological characteristics and partial DNA barcoding data, the consensus identification for this sample was determined to be *S. hemipinnis*.

2. Taxonomic revision/ redescription/ new species assignments

Taxonomic revisions present yet another challenge that impedes the comprehensive utilization of DNA barcodes in databases. Many of these barcodes were deposited prior to taxonomic revisions and have not been updated, resulting in classification of samples under different names. Such instances were observed in *Centrophorus uyato*, several species of rays in the family Dasyatidae and *Aetobatus ocellatus*, where they were matched with species bearing the names before revisions. Consensus as to species was drawn based on morphological characteristics and published distributional ranges of taxa in current literature. The names used when the samples were deposited were likely synonyms and require updating. Our DNA sequence data represented and comparable with recent DNA information deposited after the revision. Nevertheless, some revised taxa, namely *T. crozieri*, lacked references for their DNA barcodes. Given the unavailability of morphology data for these references, we attempted to establish DNA barcoding profiles that aligned with their corresponding morphological characteristics under the assigned names.

The samples of *Glaucostegus* in this study were previously recognized as *Glaucostegus* cf. *granulosus* due to their genetic similarity to this species but still showed morphological differences. Our DNA sequences matched an undescribed *Glaucostegus* sp. later named as a distinct species. The updated publication of the sequence information in the NCBI website allowed us to place the valid name on these samples as *G. younholeei* [44~~3~~].

The reassignment of species in the genus *Myliobatis* clarified the presence of two closely related species, *M. hamlyni* and *M. tubijei*. *Myliobatis hamlyni* was previously considered a junior synonym of *M. tobijei* but later confirmed as a valid species. The name applied to

sequences of *M. tobijei* that matched with our sample were likely following the original designation, not subsequently updated. No sequences of *M. hamlyni* were available for comparison with our data. Based on morphological examination and distribution range, we conclude that the consensus species of this sample is *M. hamlyni*.

~~-~~The houndshark sample was initially identified as *Iago mangalorensis* based on morphological similarities, which more closely resembled this species than other members of the genus *Iago*, prior to the formal description of the new species *I. gopalakrishnani* [43]. At the time, genetic data for *I. mangalorensis* were not available for comparison. Although our sample clustered with several undescribed *Iago* species, it was provisionally designated as *I. mangalorensis*. Following the formal description of *I. gopalakrishnani*, the sample is now considered to belong to this newly recognized species.

3. Cryptic morphological similarity

There were discrepancies in the labeling of sequences when comparing the NCBI and BOLD databases for a few species, such as *Chiloscyllium hasseltii*, which closely resembles *Chiloscyllium~~C.~~ griseum*; *Carcharhinus falciformis*, misidentified as *Carcharhinus~~C~~. brevipinna; Carcharhinus~~C.~~ sorrah*, misidentified as *Carcharhinus~~C.~~ seali*; and *Maculabatis gerrardi*, occasionally misidentified as *M. macrura*. These species pairs highly similar may be misidentified by non-specialists. For example, *Chiloscyllium~~C.~~ hasseltii* and *Chiloscyllium~~C.~~ griseum* are morphologically similar as adults but can be distinguished in the juvenile stage based on the presence of dark brown bands. Similarly, *M. gerrardi* and *M. macrura* both exhibit white spots on their bodies but differ in the distribution of these markings and the morphometric ratios (head length to disc width and disc length to disc width).

Some species are unfamiliar and sometimes misidentified as related species such as *Mustelus stevensi* occasionally misidentified as *M. lenticulatus*, *Narcine timlei* occasionally misidentified as *N. brunnea*. These samples exhibited distinct morphological characteristics from their congeners. In such cases, we placed reliance on the morphological characteristics of these species, recognizing the potential for mislabeling in the references. Furthermore, we cross-referenced synonyms of these species with available literature, giving priority to accepted names. Consequently, the consensus species identification remained consistent with the initially assigned species name.

Sequences under name *Narcine prodorsalis* were either not present or unavailable for public access in both NCBI and BOLD. However, our *N. prodorsalis* COI sequence matched with an undescribed *Narcine* sp. and one *N. maculata*. The phylogenetic placement of our sample and the other *Narcine* ~~spp.~~ species showed that the *N. maculata* that matched our sequence was likely mislabeled because all other *N. maculata* reference sequences formed a distinct clade.

4. Discordance between morphology and genetics

We also encountered discrepancies between morphology and genetics as in the case of *Bythaelurus* cf. *lutarius*. This species exhibited clear morphological differences from its ~~sympatric~~ counterpart, *B. hispidus*, and other congeners. However, their COI sequences aligned with that of *B. hispidus* (Fig 1B) while ND2 sequences did not align~~ed~~ with any *Bythaelurus*

Formatted: Font: Italic

Formatted: Font: Italic

Formatted: Font: Italic

Formatted: Font: Italic

Formatted: Font: Italic

Formatted: Font: Italic

Formatted: Font: Italic

species (Fig 2B). These morphological distinctions included the absence of papillae in the tongue and roof of the mouth, variations in head shape and differences in the origins of the dorsal fins [5]. As a result, a consensus identification could not be definitively established, and the specimens were provisionally assigned as *B.* cf. *lutarius*.

Another challenging case was found in a sample morphologically identified as *Etmopterus fusus*. The COI sequence of this sample did not match any references in both NCBI and BOLD while its ND2 sequence matched an *E. splendidus* sample from Taiwan. Our sample from the Andaman Sea displayed clear morphological characteristics consistent with *E. fusus*, including the presence of four black bands above the anal fin continuing to the caudal fin. This feature distinguished it from *E. splendidus*, which displayed a different black band pattern in the same position. The distribution ranges of these 2 species are clearly different with *E. fusus* in the Eastern Indian Ocean and northern Western Australia, and *E. splendidus* in the Northwestern Pacific Ocean. The single reference COI sequence of *E. fusus* which came from a sample in New Caledonia showed a genetic distance from our sample greater than 6% based on 659 bp. Based on the morphological characteristics and the collecting locality of our sample, the consensus identification was *E. fusus*.

The average intraspecific genetic distance for sharks and batoids across both gene fragments, COI and ND2, was about 1% (Table 3). However, the maximum intraspecific genetic distance was much greater. ~~In sharks, the highest COI intraspecific variation was observed between our *Etmopterus fusus* sample and the NCBI reference, while the greatest ND2 variation was observed in our *Echinorhinus brucus* sample and the reference.~~ In sharks, the highest COI variation was between our *Etmopterus fusus* sample and references. A similar ND2 pattern was seen in *Echinorhinus brucus*. Among batoids, samples of *Brevitrygon imbricata* and *Aetobatus flagellum* presented highest intraspecific genetic distances for both genes. ~~Additionally~~Notably, *Himantura leoparda* and *Narcine* spp. showed COI intraspecific variation exceeding 4%. The average genetic differentiation among congeneric species exceeded 7% for both gene fragments. ~~Additionally, *Himantura leoparda* and *Narcine* spp. showed COI intraspecific variation exceeding 4%.~~

Formatted: Font: Italic

Formatted: Font: Italic

Table 3. Genetic distance (K2P percentage) based on COI and ND2 of elasmobranch species.

**Levels of genetic comparison**	**Taxa**	**COI**			**ND2**		
		Mean (S.E.)	Min	Max	Mean (S.E.)	Min	Max
Variations among individual of the same species	Sharks	0.92 (0.26)	0.00	6.32	0.76 (0.21)	0.00	5.37
	Batoids	0.86 (0.13)	0.00	5.12	1.26 (0.31)	0.00	10.49
Variations among species of the same genus	Sharks	7.50 (0.54)	0.05	17.73	10.88 (0.57)	0.39	21.01
	Batoids	10.42 (0.45)	0.21	26.40	11.31 (0.59)	0.58	32.38

However, very low interspecific genetic distances were observed between our *Etmopterus fusus* sample and the NCBI reference of *E. splendidus* and between our *Mustelus*

stevensi sample and the reference *M. lenticulatus*. Among batoids, very low interspecific genetic variation was observed in *Neotrygon kuhlii* and other *Neotrygon* spp. species and also within the genus *Telatrygon*. Similarly, low genetic variation was observed among species of *Rhinoptera*, *Aetobatus*, *Mobula*, and *Myliobatis* in the NCBI reference sequences.

The distribution of K2P genetic distances in sharks and batoids revealed distinct differences between intra- and interspecific variation (Figs 5, 6). Over 80% of our samples fell within 2% genetic distance of their assigned species. However, interspecific genetic variation varied across both genes. Some genera, such as *Etmopterus* and *Echinorhinus*, showed highly similar sequences, likely due to limited available genetic data. In contrast, other genera, such as *Neotrygon*, *Telatrygon*, and *Aetobatus*, have undergone taxonomically revisions, and current scientific names of many sequences have not been updated.

Sequences from our samples of *Iago mangalorensis*, *Rhinobatos ranongensis*, *Cruriraja andamanica*, and *Hemitrygon laosensis*, did not match any published species since reference sequences were unavailable. These DNA sequences showed distinct genetic diversity compared to their congeneric species.

Figure 5. Distribution of K2P genetic distance in sharks (A) COI, arrow pointing to *Etmopterus fusus*; (B) ND2, arrow pointing to *Echinorhinus brucus*.

Figure 6. Distribution of K2P genetic distance in batoids (A) COI, arrows pointing *to Narcine timlei*, *N. brunnea*, *Brevitrygon imbricata*; (B) ND2 arrows pointing to *Brevitrygon imbricata*, *Aetobatus flagellum*.

Discussion

The implementation of DNA barcoding significantly enhances the taxonomic clarification of cartilaginous fishes, offering an alternative to the intricate and time-consuming process of species identification for non-taxonomists [15, 51]. However, morphological examination still remains the key method for non-taxonomists [15, 48]. Among the well-recognized mitochondrial gene candidates serving as DNA barcodes for cartilaginous fishes, COI and ND2 stand out [7, 12, 13, 15, 20]. COI tends to demonstrate higher success rates in amplification compared to ND2 due to its shorter length. The design of shorter fragments within the ND2 region amplification process has bolstered its efficacy, providing additional and valuable insights for future application. It is important not to pass judgment on the superiority of one gene over the other, as each presents distinct advantages. COI has enjoyed a decade-long legacy as a DNA barcode for fishes [12, 13, 15, 21, 22], whereas the acceptance of ND2 as a DNA barcode for chondrichthyans came later [7, 5249]. The extensive utilization of COI is mirrored by the larger repository of COI sequences available across databases in comparison to ND2 sequences. However, ND2 sequences offer the advantage of increased length, subsequently affording more characteristics for meaningful comparison. Consequently, a synergistic employment of both genes holds promise in the accurate assessment of elasmobranch diversity.

The number of ND2 sequences available for comparison is fewer than COI. COI still serves as important DNA barcode but need serious taxonomic updating.

The utility of DNA barcoding has contributed to new species records [5, 22, 53, 54~~50. 51~~]. In this study, DNA barcoding clarified specimens initially identified as *Glaucostegus* cf. *granulatus* [5], confirming them as *Glaucostegus younholeei* [44~~43~~]. Additionally, DNA barcoding aids in identifying cryptic species, leading to new species descriptions and taxonomic revisions [e.g.~~ 52–58~~55-61].

Despite the considerable promise of DNA barcodes, this study has revealed several limitations. The effectiveness of this tool is contingent upon the availability of data from verified species, which is absent or limited from, especially, deep-sea species. Because of the rarity of specimens, identification based on morphological criteria may be mistaken resulting in erroneous molecular sequence assignment. The description of a new species, *Glaucostegus younholeei* sp. nov. [44~~43~~], provided a clear identification of samples of the guitarfish previously described as *Glaucostegus* cf. *granulatus* [5] although the reference sequences in NCBI have not been updated and listed the species as *Glaucostegus* sp. The authors of the new species have included a reference to the description of their newly described species as an update, hence providing an official name for the taxon. In addition, the presence of synonyms for certain species, such as the zebra shark, has also presented a problem, with two names existing in the databases. Although a priority is accorded to the senior synonym, *Stegostoma tigrinum* [~~59~~55], several sequences of this species are still listed as *S. fasciatum*.

The issue of cryptic morphological similarity, due to either specimen misidentification or failure to take account of taxonomic revision, also presents a significant challenge when utilizing public databases for species verification. In such cases, it becomes necessary to rely on the morphological characteristics of specimens to confirm species assignment. The landscape of taxonomic revisions within many taxa adds complexity to the direct utilization of DNA barcodes, as species names undergo alterations over time. For example, DNA barcodes of samples identified as *Centrophorus uyato* have been matched with different *Centrophorus* species due to recent revisions that revealed *C. granulosus* as a species complex [42, 56~~52~~]. As a result, several COI of several species including *C. uyato* were erroneously labeled as *C. granulosus*. This problem is also observed in *Maculabatis gerrardi* complex [57]. On the contrary, information on ND2 sequences is generally more recent and up-to-date serving as a better reference than COI. A similar situation was found in *Aetobatus ocellatus* that was previously grouped within the *A. narinari* species complex but later recognized as a valid species based on molecular evidence [~~47, 53~~50, 58]. Sequences of samples labeled as *A. narinari* prior to this taxonomic revision could actually represent *A. ocellatus*, emphasizing the need for caution when using such references.

The extensive revision of stingrays of the family Dasyatidae has brought about significant changes in generic names and the erection of new species exemplified by *Brevitrygon* [~~38, 54~~45], *Neotrygon* [47~~11~~], and *Telatrygon* [~~55~~59]. These species belonging to these genera exhibit

Formatted: Font: Italic

distinct morphological characteristics that serve as a primary mean of identification, albeit requiring the expertise of specialists for their precise differentiation. To accelerate the identification process, DNA profiles of these species should be established. This study demonstrated such genetic distinctions through DNA barcoding, which contributes to the improved assessment of biodiversity.

Several sharks and rays are still subject to taxonomic revision due to their morphological similarity and very wide distribution ranges. The *Squalus* species complex presents wide distribution range and similar morphological characteristics among congeners [~~56–58~~60-62]. Some species are thought to be endemic to certain geographic locations, but their distribution is still unclear and requires further examination, which may result in further taxonomic revision [36]. Our *Squalus* samples provisionally identified as *S. hemipinnis* and *S. montalbani*, which did not match the reference sequences given for those taxa may be distinct lineages.

Discordance between morphology and the DNA barcode assigned to *Bythaelurus* cf. *lutarius* provided difficulty in reaching consensus identification. Identification based on ~~distinctive~~ morphological characteristics revealed that all examined samples collected from the Thai Andaman Sea were of *B. lutarius* [5]~~.,~~. This species, first described as *Halaelurus lutarius* [63], is clearly distinguishable from similar looking species in the same genus [64-69]. ~~clearly distinguishable from the only similar-looking species found in the same region, *B. hispidus* [60], *B. stewarti* [61], *B. naylori* [62], *B. dawsoni* and *B. canescens* [63].~~ The lack of COI sequences of *B. lutarius* from the type locality and the unavailability of the ND2 sequence of ~~-~~*B. hispidus*~~-~~, despite being studied [67], poses a challenge~~s~~ to the use of DNA barcoding for species confirmation. Because all *B.* cf. *lutarius* sampled formed a monophyletic cluster based on longer ND2 fragments, clearly distinct from other catsharks whose sequences were available for comparison, they likely all represented the same species. Although the samples show ~~With~~ clear morphological distinction, their location falls outside the known distribution range of *B. lutarius* and ~~uncertain~~ their genetic affinity within this genus remains uncertain, the samples are tentatively assigned as *B.* cf. *lutarius*.

It has been long recognized that intraspecific genetic variations among metazoans generally fall within 2% [13, 15, 20–22]. However, low genetic variation among distinct but congeneric lineages is evident in *Mustelus* species [~~64~~70]. The clear genetic differentiation between *M. stevensi* and *M. lenticulatus* [10] enabled accurate consensus identification~~, which allowed a correct consensus identification~~. On the contrary, certain species, such as *Etmopterus fusus*, *Squalus montalbani*, *Himantura leoparda*, *Maculabatis gerrardi*, and *Narcine timlei*, display high genetic variation within species. This may arise from either cryptic diversity or misidentification of reference samples as seen in the species complex of *Himantura uarnak* [71], *Maculabatis gerrardi* [57], and *Narcine* species [72]~~.~~. Therefore, while a 2% genetic difference within species is helpful, it cannot serve as an absolute criterion for species delimitation. Comparisons of genetic differentiation based on COI and ND2 fragments showed slightly greater interspecific differences in ND2 compared to COI possibly due to the utilization of longer

Formatted: Font: Italic

Formatted: Font: Italic

Formatted: Font: Italic

Formatted: Font: Italic

Formatted: Font: Italic

Formatted: Font: Italic

fragments in the analyses. Nevertheless, low genetic differentiation among distinct congeneric species may confound their recognition. For example, our *Etmopterus fusus* sample showed high COI sequence variation compared to reference *E. fusus* but similar a ND2 sequence compared to *E. splendidus*. Although both species are closely related and rarely available for thorough examinations [~~65~~73], clear difference in their geographical distribution [~~66~~74] helps identify our sample as *E. fusus*. While the influx of DNA barcoding sequences is expected to continue, its application should be approached with caution. Given the dearth of expert chondrichthyan taxonomists at both local and global scales, the utilization of molecular markers is important for facilitating biodiversity assessment [~~67~~75]. The data gathered by our present study contribute both to the sample of genetic information from better-known species and new genetic information of several species not available in public databases. Despite this great benefit, the clarity of the species concept is crucial, and traditional taxonomic identification methods should not be disregarded [~~68~~76].

Discordance between morphological and genetic characteristics and misidentification of closely related species may result from hybridization, which has been reported many times in elasmobranchs [~~69–74~~77-82]. In such cases, DNA barcoding cannot resolve taxonomic identification and necessitates assessment of nuclear DNA. While hybridization and introgression have been documented in a few elasmobranch groups, many remain unexplored. Given that environmental changes may influence reproductive behaviors of these fishes, baseline data on genetics across different species are crucial for understanding these processes [80].

As elasmobranchs serve as resources for both local consumption and international export, prohibition of the use of these resources is impractical. On the other hand, regulation of fisheries based on solid scientific foundation and correct species assignment will help sustain long-term exploitation of these resources. To achieve this level of regulation, fundamental knowledge on species and genetic diversity is important and requires thorough examination, especially of local populations [18]. DNA barcoding data then serve as references for the authentication of meat products [14, 83~~75~~]. However, since taxonomic revisions are ongoing and updates may not always keep pace, maintaining well-curated voucher libraries and local reference collections is essential for accurate species verification. Limited data on biodiversity jeopardize populations of these fishes, especially those commonly used in the preparation of luxurious dishes and pharmaceutical products, which usually come from non-specified species and some of which are globally vulnerable species [~~76–80~~84-88].

Acknowledgements

We would like to thank our assistants, P. Chanmuang. T. Meejan, P. Laongdee, K. Panpong, A. Pankhort, and P. Wongmanee, who contributed to sample collection and laboratory work. Several organizations and individuals provided specimens for this project, including the Marine Fisheries Research and Development Division and Inland Fisheries Research Development Division of the Department of Fisheries, the Thailand National Science Museum, Department of

Marine and Coastal Resources, C. Vidthayanon, and T. Vasinopas. This project is funded by National Research Council of Thailand (NRCT-N25A650485). Language editing was assisted by P. D. Round. Permission for animal use was granted by MU-IACUC (permission MUSC62-025-489) and Burapha U-ethics. The authors declare that they do not have any known financial interests or personal relationships that could have appeared to influence the work reported in this paper.

Financial disclosure

The funders had no role in study design, data collection and analysis, decision to publish, or preparation of the manuscript.

Formatted: Font: Bold

References

1. Weigmann S. Annotated checklist of the living sharks, batoids and chimaeras (Chondrichthyes) of the world, with a focus on biogeographical diversity: Annotated global checklist of Chondrichthyes. J Fish Biol. 2016;88(3):837–1037.
2. Ali A, Fahmi, Dharmadi, Krajangdara T, Khiok ALP. Biodiversity and habitat preferences of living sharks in the Southeast Asian region. Indones Fish Res J. 2018; 24: 133-140.
3. Voris HK. Maps of Pleistocene sea levels in Southeast Asia: shorelines, river systems and time durations. J Biogeogr. 2000;27(5):1153–67.
4. Costello MJ, Tsai P, Wong PS, Cheung AKL, Basher Z, Chaudhary C. Marine biogeographic realms and species endemicity. Nat Commun. 2017;8(1):1057.
5. Krajangdara T, Vidthayanon C, Ali A, Lim A, Sumontha M, Rodpradit R, et al. 2022. The Cartilaginous Fishes of Thailand and Adjacent Waters. Department of Fisheries, Ministry of Agriculture and Cooperatives. Chiang Mai, Thailand: Vanidakarnpim Publishing; 2022. Thai.
6. Krajangdara T, Fahmi, Ebert DA, Chaorattana C, and Khudamrongsawat J. Morphological and genetic evidence confirmed three new records of ghost shark species (Chimaeriformes) from the Andaman Sea of Thailand. Trop Nat His. 2021;21:218-233.
7. Naylor GJP, Caira JN, Jensen K, Rosana KAM, White WT, Last PR. A DNA sequence–based approach to the identification of shark and ray species and its implications for global elasmobranch diversity and parasitology. B Am Mus Nat Hist. 2012;367:1–262.
8. Khudamrongsawat J, Bhummakasikara B, Chansue N. Preliminary study of genetic diversity in the giant freshwater stingray, *Himantura chaophraya* (Batoidea: Dasyatidae) from the remnant populations in Thailand. Trop Nat His. 2017;17:175-180.
9. Last PR, Séret B, Naylor GJP. Description of *Rhinobatos ranongensis* sp. nov. (Rhinopristiformes: Rhinobatidae) from the Andaman Sea and Bay of Bengal with a review of its northern Indian Ocean congeners. Zootaxa. 2019;4576: 257–287.
10. White WT, Arunrugstichai S, Naylor GJP. Revision of the genus *Mustelus* (Carcharhiniformes: Triakidae) in the northern Indian Ocean, with description of a new species and a discussion on the validity of *M. walkeri* and *M. ravidus*. Mar Biodivers 2021; 51: 42.
11. Borsa P. Comments on "Annotated checklist of the living sharks, batoids and chimaeras (Chondrichthyes) of the world, with a focus on biogeographical diversity" (Weigmann, 2016). J Fish Biol. 2016;90: 1170-1175.Weignmann S. Reply to Borsa (2017): Comment

on 'Annotated checklist of the living sharks, batoids and chimaeras (Chondrichthyes) of the world, with a focus on biogeographical diversity by Weigmann (2016)'. J Fish Biol. 2017; 90(4): 1176–1181.
12. Ward RD, Zemlak TS, Innes BH, Last PR, Hebert PDN. DNA barcoding Australia's fish species. Philos Trans R Soc Lond B Biol Sci. 2005;360(1462):1847–1857
13. Ward RD, Holmes BH, White WT, Last PR. DNA barcoding Australian chondrichthyans: results and potential uses in conservation. Mar Freshwater Res. 2008;59:57-71.
14. Holmes BH, Steinke D, Ward RD. Identification of shark and ray fins using DNA barcoding. Fish Res. 2009;95(2–3):280–288.
15. Cerutti-Pereyra F, Meekan MG, Wei N-WV, O'Shea O, Bradshaw CJA, Austin CM. Identification of rays through DNA barcoding: an application for ecologists. PLoS One. 2012;7(6):e36479.
16. Avise JC. Molecular Markers, Natural History, and Evolution. 2nd ed. Massachusetts: Sinauer, Sunderland; 2004.
17. Frankham R. Where are we in conservation genetics and where do we need to go? Conserv Genet. 2010;11(2):661–663.
18. Domingues RR, Hilsdorf AWS, Gadig OBF. The importance of considering genetic diversity in shark and ray conservation policies. Conserv Genet. 2018;19(3):501–525.
19. Moore ABM, White WT, Ward RD, Naylor GJP, Peirce R. Rediscovery and redescription of the smoothtooth blacktip shark, *Carcharhinus leiodon* (Carcharhinidae), from Kuwait, with notes on its possible conservation status. Mar Freshw Res. 2011;62(6):528.
20. Ward RD. DNA barcode divergence among species and genera of birds and fishes. Mol Ecol Resour. 2009;9(4):1077–1085.
21. Pavan-Kumar A, Gireesh-Babu P, Suresh Babu PP, Jaiswar AK, Prasad KP, Chaudhari A, et al. DNA barcoding of elasmobranchs from Indian Coast and its reliability in delineating geographically widespread specimens. Mitochondrial DNA. 2013;26(1):92–100.
22. Loh K-H, Lim K-C, Then AY-H, Adam S, Leung AJ-X, Hu W, et al. Advancing DNA barcoding to elucidate elasmobranch biodiversity in Malaysian waters. Animals. 2023;13(6):1002.
23. Kashiwagi T, Marshall AD, Bennett MB, Ovenden JR. The genetic signature of recent speciation in manta rays (*Manta alfredi* and *M. birostris*). Mol Phylogenet Evol. 2012;64(1):212–218.
24. Ariza AA, Adachi AMCL, Roque P, Hazin FHV, Vianna M, Rotundo MM, et al. DNA barcoding and species delimitation for dogfish sharks belonging to the *Squalus* genus (Squaliformes: Squalidae). Diversity. 2022;14(7):544.
25. Leray M, Knowlton N, Ho S-L, Nguyen BN, Machida RJ. GenBank is a reliable resource for 21st century biodiversity research. Proc Natl Acad Sci USA. 2019;116(45):22651–22656.
26. Pentinsaari M, Ratnasingham S, Miller SE, Hebert PDN. BOLD and GenBank revisited – Do identification errors arise in the lab or in the sequence libraries? PLoS One. 2020;15(4):e0231814.
27. Fontes JT, Vieira PE, Ekrem T, Soares P, Costa FO. BAGS: An automated Barcode, Audit & Grade System for DNA barcode reference libraries. Mol Ecol Resour. 2021;21(2):573–583.

Formatted: Font: Italic

28. Grant DM, Brodnicke OB, Evankow AM, Ferreira AO, Fontes JT, Hansen AK, et al. The future of DNA barcoding: Reflections from early career researchers. Diversity. 2021;13(7):313.
29. Mota M, Thomaz M, Fabrin C, Diamante NA, Alessandra, Filho HO, et al. DNA barcode is efficient for identifying bat species. J Mamm Evol. 2021;29(1):63–75.
30. Peláez ML, Horreo JL, García-Jiménez R, Valdecasas AG. An evaluation of errors in the mitochondrial COI sequences of Hydrachnidia (Acari, Parasitengona) in public databases. Exp Appl Acarol. 2022;86(3):371–384.
31. Baena-Bejarano N, Reina C, Martínez-Revelo DE, Medina CA, Tovar E, Uribe-Soto S, et al. Taxonomic identification accuracy from BOLD and GenBank databases using over a thousand insect DNA barcodes from Columbia. PLoS One. 2023;18: e0277379.
32. Lindner MF, Gonçalves LT, Bianchi FM, Ferrari A, Cavalleri A. Tiny insects, big troubles: a review of BOLD’s COI database for Thysanooptera (Insecta). B Entomol Res. 2023;113: 703-715.
33. Ruedi M, Manzinallii J, Dietrich A, Vinciguerra L. Shortcoming of DNA barcodes: a perspective from the mammal fauna of Switzerland. Hystrix it J Mamm. 2023;34:54-61.
34. Last PR, White WT, de Carvalho MR, Séret B, Stehmann FW, Naylor GJP. Rays of the World. Ithaca: Cornell University Press; 2016.
35. Krajangdara T. The Cartilaginous Fishes (Sharks, Rays and Chimaeras) Found in Thai Waters and the Adjacent Areas. Marine Fisheries Research and Development Division, Department of Fisheries, Ministry of Agriculture and Cooperatives. Phuket: Phuketoffset Publishing; 2017. Thai.
36. Ebert, DA, Dando M, Fowler S. Sharks of the World – A Complete Guide. New Jersey: Princeton University Press; 2021.
37. Tamura K, Stecher G, Kumar S. MEGA11: Molecular Evolutionary Genetics Analysis version 11. Mol Biol Evol. 2021;38(7):3022–3027.
38. Last PR, Naylor GJP, Manjaji-Matsumoto BM. A revised classification of the family Dasyatidae (Chondrichthyes: Myliobatiformes) based on new morphological and molecular insights. Zootaxa. 2016a;4139(3):345–368.
39. Kalyaanamoorthy S, Minh BQ, Wong TKF, von Haeseler A, Jermiin LS. ModelFinder: fast model selection for accurate phylogenetic estimates. Nat Methods. 2017;14(6):587–589.
40. Ronquist F, Teslenko M, van der Mark P, Ayres DL, Darling A, Höhna S, et al. MRBAYES 3.2: Efficient Bayesian phylogenetic inference and model selection across a large model space. Syst Biol. 2012;61:539-542.
41. Rambaut A. FigTree v1. 3.1. Institute of Evolutionary Biology, University of Edinburgh, Edinburgh [Internet]. 2010.
42. White WT, Guallart J, Ebert DA, Naylor GJP, Veríssimo A, Cotton CF, et al. Revision of the genus *Centrophorus* (Squaliformes: Centrophoridae): Part 3—redescription of *Centrophorus uyato* (Rafinesque) with a discussion of its complicated nomenclatural history. Zootaxa. 2022;5155:1-50.~~Habib KA, Islam MJ. Description of a new species of giant guitarfish, *Glaucostegus younholeei* sp. nov. (Rhinopristiformes: Glaucostegidae) from the northern Bay of Bengal, Bangladesh. Zootaxa. 2021;4995(1):129–146.~~

43. Bineesh KK, Beura S, Das M, Nashad M, Akhilesh KV. Description of a new species of hound shark of the genus *Iago* (Carcharhiniformes: Triakidae) from the northern Indian Ocean. Indian J Fish. 2025;72(1):18-28.

44. Habib KA, Islam MJ. Description of a new species of giant guitarfish, *Glaucostegus younholeei* sp. nov. (Rhinopristiformes: Glaucostegidae) from the northern Bay of Bengal, Bangladesh. Zootaxa. 2021;4995(1):129–146.~~White WT, Guallart J, Ebert DA, Naylor GJP, Veríssimo A, Cotton CF, et al. Revision of the genus *Centrophorus* (Squaliformes: Centrophoridae): Part 3—redescription of *Centrophorus uyato* (Rafinesque) with a discussion of its complicated nomenclatural history. Zootaxa. 2022;5155:1-50.~~

~~43.~~45. Last PR, Weigmann S, Naylor GJ. The Indo-Pacific stingray genus *Brevitrygon* (Myliobatiformes: Dasyatidae): Clarification of historical names and description of a new species, *B. manjajiae* sp. nov., from the Western Indian Ocean. Diversity. 2023;~~12;~~15(12):1213.

46. Borsa P, Shen K-N, Arlyza IS, Hoareau TB. Multiple cryptic species in the blue-spotted maskray (Myliobatoidei: Dasyatidae: *Neotrygon* spp.): An update. C R Biol. 2016;339(9–10):417–26.

~~44.~~47. Last PR, White WT, Séret B. Taxonomic status of maskrays of the *Neotrygon kuhlii* species complex (Myliobatoidei: Dasyatidae) with the description of three new species from the Indo-West Pacific. Zootaxa. 2016;4083(4):533-561.

~~45.~~48. Borsa P, Arlyza IS, Hoareau TB, Shen K-N. Diagnostic description and geographic distribution of four new cryptic species of the blue-spotted maskray species complex (Myliobatoidei: Dasyatidae; *Neotrygon* spp.) based on DNA sequences. J Oceanol Limnol. 2018;36(3):827–41.

~~46.~~49. White WT, Kawauchi J, Corrigan S, Rochel E, Naylor GJP. Redescription of the eagle rays *Myliobatis hamlyni* Ogilby, 1911 and M. tobijei Bleeker, 1854 (Myliobatiformes: Myliobatidae) from the East Indo-West Pacific. Zootaxa. 2015;3948(3):521–48.

~~47.~~50. White WT, Last PR, Naylor GJP, Jensen K, Caira JN. Clarification of *Aetobatus ocellatus* (Kuhl, 1823) as a valid species, and a comparison with *Aetobatus narinari* (Euphrasen, 1790) (Rajiformes: Myliobatidae). In: Last PR, White WT, Pogonoski JJ. (Eds.), Descriptions of new sharks and rays from Borneo. CSIRO Marine and Atmospheric Research Paper 032. 2010;32:141-164.

~~48.~~51. Gostel MR, Kress WJ. The expanding role of DNA barcodes: Indispensable tools for ecology, evolution, and conservation. Diversity (Basel). 2022;14(3):213.

~~49.~~52. Henderson AC, Reeve AJ, Jabado RW, Naylor GJP. A molecular-assisted taxonomic assessment of elasmobranch fishes (Chondrichthyes: Elasmobranchii) from south-eastern Ara~~i~~bia revisited. Zootaxa. 2025;5575:429-446.

~~50.~~53. Fernando D, Bown RMK, Tanna A, Gobiraj R, Ralicki H, Jockusch EL, et al. New insights into the identities of the elasmobranch fauna of Sri Lanka. Zootaxa. 2019;4585(2):201-238.

~~51.~~54. Zhang M, Shan B, Liu Y, Wang L, Yang C, Liu M, et al. A new record of *Squalus montalbani* (Chondrichthyes: Squaliformes: Squalidae) from the Nansha (Spratly) Island, South China Sea. AleP. 2023; 53: 51-57.

Formatted: Font: (Default) Times New Roman, 12 pt

Formatted: Font: Italic

Formatted: Font: Italic

Formatted: Font: Italic

Formatted: Font: Italic

52.55. Dahl RB, Sigsgaard EE, Mwangi G, Thomsen PF, Jørgensen RD, Torquato F de O, et al. The sandy zebra shark: A new color morph of the zebra shark *Stegostoma tigrinum*, with a redescription of the species and a revision of its nomenclature. Copeia. 2019;107(3):524.

56. White WT, Ebert DA, Naylor GJP, Ho H, Clerkin P, Verissimo A, et al. Revision of the genus *Centrophorus* (Squaliformes: Centrophoridae): Part 1—Redescription of *Centrophorus granulosus* (Bloch & Schneider), a senior synonym of *C. acus* Garman and *C. niaukang* Teng. Zootaxa. 2013;3752:035-072.

53.57. Muhala V, Guimarães-Costa A, Macate IE, Lutz Í, Bessa-Silva AR, et al. A possible new species of the *Maculabatis gerrardi* complex (Dasyatidae: Urogymninae) in the Indian Ocean coast off Southeastern Africa. J Mar Biol Assoc UK. 2024;104:e107,1-4.

54. Richards VP, Henning M, Witzell W, Shivji MS. Species delineation and evolutionary history of the globally distributed spotted eagle ray (*Aetobatus narinari*). J Hered. 2009;100(3):273–83.

55.58. ~~Last PR, Weigmann S, Naylor GJ. The Indo-Pacific stingray genus *Brevitrygon* (Myliobatiformes: Dasyatidae): Clarification of historical names and description of a new species, *B. manjajiae* sp. nov., from the Western Indian Ocean. Diversity. 12;15(12):1213.~~

56.59. Last PR, White WT, Naylor G. Three new stingrays (Myliobatiformes: Dasyatidae) from the Indo-West Pacific. Zootaxa. 2016e;4147(4):377–402.

57.60. Marouani S, Chaâba R, Kadri H, Saidi B, Bouain A, Maltagliati F, et al. Taxonomic research on *Squalus megalops* (Macleay, 1881) and *Squalus blainvillei* (Risso, 1827) (Chondrichthyes: Squalidae) in Tunisian waters (central Mediterranean Sea). Sci Mar. 2012;76(1):97–109.

58.61. Bonello JJ, Bonnici L, Ferrari A, Cariani A, Schembri PJ. Not all that clear cut: Intraspecific morphological variability in *Squalus blainville* (Risso, 1827) and implication for identification of this species. J Mar Biol Assoc UK. 2016;96(8):1585-1596.

62. Ferrari A, Di Crescenzo S, Cariani A, Crobe V, Benvenuto A, Piattoni F, et al. Puzzling over spurdogs: molecular taxonomy assessment of the *Squalus* species in the Strait of Sicily. Eur Zool J. 2021;88(1):181-190.

63. Springer S, D'Aubrey JD. Two new Scyliorhinid sharks from the east coast of Africa, with notes on related species. Oceanographic Research Institute, Investigational Report. 1972; 29: 1–19.

64. Springer, S. A revision of the catsharks, family Scyliorhinidae. NOAA Technical Report NMFS Circular. 1979;422:1–152.

65. Ebert DA, Clerkin PJ. A new species of deep-sea catshark (Scyliorhinidae: *Bythaelurus*) from the southwestern Indian Ocean. JOSF. 2015;15:53-63.

66. Kaschner CJ, Weigmann S, Thiel R. *Bythaelurus tenuicephalus* n. sp., a new deep-water catshark (Carcharhiniformes, Scyliorhinidae) from the western Indian Ocean. Zootaxa. 2015;4013(1):120–138.

67. Weigmann S, Ebert DA, Clerkin PJ, Stehmann MF, Naylor GJ. *Bythaelurus bachi* n. sp., a new deep-water catshark (Carcharhiniformes, Scyliorhinidae) from the southwestern Indian Ocean, with a review of *Bythaelurus* species and a key to their identification. Zootaxa. 2016;4208(5):401–432.

Formatted: Adjust space between Latin and Asian text, Adjust space between Asian text and numbers, Tab stops: Not at 0.38"

Formatted: Font: (Default) Times New Roman, 12 pt

Formatted: Font: (Default) Times New Roman, 12 pt

Formatted: Font: Italic

Formatted: Font: Italic

Formatted: Font: Italic

59.
60.68. Akhilesh KV, White WT, Bineesh KK, Ganga U, Pillai NGK. Biological observations on the bristly catshark *Bythaelurus hispidus* from deep waters off the south-west coast of India. J Fish Biol. 2013; 82: 1582-1591. Weigmann S, Kaschner CJ. *Bythaelurus vivaldii*, a new deep-water catshark (Carcharhiniformes, Scyliorhinidae) from the northwestern Indian Ocean off Somalia. Zootaxa. 2017;4263(1):97–119.
61.69. Weigmann S, Kaschner CJ, Thiel R. A new microendemic species of the deep-water catshark genus *Bythaelurus* (Carcharhiniformes, Pentanchidae) from the northwestern Indian Ocean, with investigations of its feeding ecology, generic review and identification key. PLoS One. 2018;13(12):e0207887.
62. Ebert DA, Clerkin PJ. A new species of deep-sea catshark (Scyliorhinidae: *Bythaelurus*) from the southwestern Indian Ocean. JOSF. 2015;15:53-63.
63. Springer S. A revision of the catsharks, family Scyliorhinidae. NOAA Tech Rep NMFS. 1979;422: p. 1-152.
64.70. Boomer JJ, Harcourt RG, Francis MP, Stow AJ. Genetic divergence, speciation and biogeography of *Mustelus* (sharks) in the central Indo-Pacific and Australasia. Mol Phylogenet Evol. 2012;64(3):697–703.
71. Arlyza I, Shen K, Solihin DD, Soedharma D, Berrebi P, et al. Species boundaries in the *Himantura uarnak* species complex (Myliobatiformes: Dasyatidae). Mol Phylogenet Evol. 2013;66:429-435.
72. Melis R, Di Crescenzo S, Cariani A, Ferrari A, Crobe V, et al. I like this new me: unravelling population structure of Mediterranean electric rays and taxonomic uncertainties within Torpediniformes. Animals. 2023;13:2899.
65.73. Straube N, Iglésias SP, Sellos DY, Kriwet J, Schliewen UK. Molecular phylogeny and node time estimation of bioluminescent Lantern Sharks (Elasmobranchii: Etmopteridae). Mol Phylogenet Evol. 2010;56(3):905–917.
66.74. Last PR, Burgess GH, Séret B. Description of six new species of lantern-sharks of the genus *Etmopterus* (Squaloidea: Etmopteridae) from the Australasian region. Cybium. 2002;26:203-223.
67.75. Engel MS, Ceríaco LMP, Daniel GM, Dellapé PM, Löbl I, Marinov M, et al. The taxonomic impediment: a shortage of taxonomists, not the lack of technical approaches. Zool J Linn Soc. 2021;193(2):381–387.
68.76. Rheindt FE, Bouchard P, Pyle RL, Welter-Schultes F, Aescht E, Ahyong ST, et al. Tightening the requirements for species diagnoses would help integrate DNA-based descriptions in taxonomic practice. PLoS Biol. 2023;21(8):e3002251.
69.77. Morgan JAT, Harry AV, Welch DJ, Street R, White J, Geraghty PT, Macbeth WG, et al. Detection of interspecies hybridization in Chondrichthyes: hybrids and hybrid offspring between Australian (*Carcharhinus tilstoni*) and common (*C. limbatus*) blacktip shark found in an Australian fishery. Conserv Genet. 2012;13:455-463.
70.78. Cruz VP, Vera M, Mendoça FF, Pardo BG, Martinez P, Oliveira C, Foresti F. First identification of interspecies hybridization in the freshwater stingrays *Potamotrygon motoro* and *P. falkneri* (Myliobatiformes, Potamotrygonidae). Conserv Genet. 2015;16:241-245.
71.79. Marino IAM, Riginella E, Gristina M, Rasotto MB, Zane L, Mazzoldi C. Multiple paternity and hybridization in two smooth-hound sharks. Sci Rep. 2015;5:12919.

Formatted: Font: Italic

Formatted: Font: Italic

Formatted: Font: Italic

72.80. Corrigan S, Delser PM, Eddy C, Duffy C, Yang L, Li C, Bazinet AL, et al. Historical introgression drives pervasive mitochondrial admixture between two species of pelagic sharks. Mol Phylogenet Evol. 2017;110:122-126.
73.81. Baker AM, Adams DH, Briggers III WB, Frazier BS, Portnoy DS. Hybridization between sympatric hammerhead sharks in the western North Atlantic Ocean. Biol Lett. 2019;15:20190004. http://dx.doi.org/10.1098/rsbl.2019.0004.
74.82. Nevatt e RJ, Williamson JE, Gillings MR. First evidence of multiple paternity and hybridization in Australian sawsharks. Mar Freshwater Res. 2023; 74(7):586-600.
75.83. Domin gues RR, Bunholi IV, Pinhal D, Antuens A, Mendoça FF. From molecule to conservation: DNA-based methods to overcome frontiers in the shark and ray fin trade. Conserv Genet Resour. 2021;13:231-247.
76.84. Wainw right BJ, Ip YCA, Neo ML, Chang JJM, Gan CZ, Clark-Shen N, et al. DNA barcoding of traded shark fins, meat and mobulid gill plates in Singapore uncovers numerous threatened species. Conserv Genet. 2018;19(6):1393–1399.
77.85. Hobbs CAD, Potts RWA, Bjerregaard Walsh M, Usher J, Griffiths AM. Using DNA barcoding to investigate patterns of species utilisation in UK shark products reveals threatened species on sale. Sci Rep. 2019;9(1):1028.
78.86. Choo MY, Choy CPP, Ip YCA, Rao M, Huang D. Diversity and origins of giant guitarfish and wedgefish products in Singapore. Aquat Conserv. 2021;31(7):1636–1649.
79.87. Liu CJN, Neo S, Rengifo NM, French I, Chiang S, Ooi M, et al. Sharks in hot soup: DNA barcoding of shark species traded in Singapore. Fish Res.2021;241:105994.
80.88. Klangnurak W, Arunrugstichai S, Manopawitr P, Krajangdara. DNA-based species identification of shark fins traded in Thai markets. Conserv. Genet. 2023;24(4):537-546.

Letter of response to reviewer's comments

- Adding the section "Financial disclosure"
- Move the ethic statements (previously in the Acknowledgements) to method section
- Separate supporting information into 2 files: S1—primers and protocol used, S2—sample identification including morphological and genetic examination of each sample.

Reviewer #1

Reviewers' comments	Response
Nevertheless, if the morphology was indeed examined, it needs to be explained how it was examined and the morphological data should be presented in the manuscript.	Morphological identification of ALL samples is in the supplementary section II. The identification was on site. Some uncertain individuals were brought to the lab for thorough examination. If identification was uncertain, they were marked as unsure.
Page 2, sentence "Available genetic information, though scant, poses many questions regarding taxonomic diversity in which the high levels of genetic differentiation that could not be ignored [11]": This message was not published in the cited paper but in the reply to that paper. Therefore, the reference needs to be changed to the following one:<br><br>Weigmann 2017: Reply to Borsa (2017): Comment on 'Annotated checklist of the living sharks, batoids and chimaeras (Chondrichthyes) of the world, with a focus on biogeographical diversity by Weigmann (2016)'. Journal of Fish Biology, 90(4), 1176–1181.	We felt that Weigmann 2017 Reply to Borsa 2017 appeared to place priority to morphology rather than genetics, and take genetics as "not important" or something that can be ignored.<br><br>However, if the reviewer feels that we should cite Weigmann 2017, we would do so.
Table 2: The correct name of the order is "CARCHARHINIFORMES"	Done.
Page 7, Iago mangalorensis: The specimens should also be compared with the very recently described Iago gopalakrishnani.	We used the name *Iago mangalorensis* before the knowledge of this publication by the time of manuscript submission.<br>We changed the name and provided the citation as suggested.<br><br>The edited version moves *Iago mangalorensis* to #2 Taxonomic revision/ redescription/ new species assignments. In table 2, the consensus species of this sample is changed to *I. gopalakrishnani*, but the phylogenetic tree remains unchanged as the genetic sequences of this new species are not available for public use. Only samples labelled as Iago sp. are.
Page 9, *2: Reference 42 is the paper currently listed as reference 43 in the reference section.	There are additional references being added. This reference is now #42
Page 9, *4: Reference 43 is the paper currently listed as reference 42 in the reference section.	There are additional references being added. This reference is now #44

Reviewers' comments	Response
Page 9, *5: Wrong paper cited, a taxonomic and biogeographic revision of Brevitrygon was carried out in the following paper:<br><br>Last, P. R., Weigmann, S., & Naylor, G. J. P. (2023). The Indo-Pacific Stingray Genus Brevitrygon (Myliobatiformes: Dasyatidae): Clarification of Historical Names and Description of a New Species, B. manjajiae sp. nov., from the Western Indian Ocean. Diversity, 15(12), 1213. https://doi.org/10.3390/d15121213	We would like to give credit to the original paper that described the genus *Brevitrygon*. We know that Last et al. (2023) provided a revision of species in this genus, but some information in GenBank referred to this genus as Himantura.<br><br>We put 2 references in Table 2.
Page 9, *6: A taxonomic revision of Neotrygon kuhlii was not carried out in the cited references but in the following reference: Last, P.R. & White, W.T. & Séret, B. (2016) Taxonomic status of maskrays of the Neotrygon kuhlii species complex (Myliobatoidei: Dasyatidae) with the description of three new species from the Indo-West Pacific. Zootaxa, 4083(4), 533–561	Reference added.
Page 9, *7-9: Write "species resurrection" instead of "new species erection".	Done
Page 10, reference 43: see my comment 6 (regarding Glaucostegus younholeei); same applies to mentioning of this reference on page 13.	Done
Page 13, first sentence of Discussion: This sentence gives the impression that barcoding can be used without making any morphological examinations. This is a very dangerous way as many examples from the literature have shown that solely relying on genetics can lead to clearly incorrect conclusions and even incorrect new species descriptions. One example for such wrong conclusions the following paper (see their Etmopterus lucifer in the order Carcharhiniformes):<br><br>Vélez-Zuazo X, Agnarsson I. Shark tales: a molecular species-level phylogeny of sharks (Selachimorpha, Chondrichthyes). Mol Phylogenet Evol. 2011 Feb;58(2):207-17. doi: 10.1016/j.ympev.2010.11.018.<br><br>The importance of integrating morphological and molecular taxonomies has been summarized, e.g., in the previously mentioned paper by Weigmann	Our intention does NOT mean that barcoding can be used without making any morphological examinations. We relied on morphological examination before the use of genetics. We use genetics to HELP with the identification when we are uncertain.<br>It is difficult to work in taxonomic studies that require long-term training and experiences. We know that there is a conflict between taxonomists and phylogeneticist.<br>The 2 references cited in this sentence (Cerutii-Pereyra et al. 2012 and Gostel and Kress 2022) just pointed out the usefulness of genetics to help with species identification.<br>We agree with the reviewer that using molecular data alone to identify species can lead to wrong conclusion. To clarify this sentence, we revised the sentence as following:<br><br>The implementation of DNA barcoding significantly enhances the taxonomic clarification of cartilaginous

Reviewers' comments	Response
(2017) (see my comment 2 for the full citation of this paper).	fishes, offering an alternative to the intricate and time-consuming process of species identification for non-taxonomists [15, 51]. However, morphological examination still remains the key method.
isPage 14: Reference 59 must be reference 52.	The reference number moved to #55.
Page 14: Reference 42 must be reference 43 and reference 52 must be reference 53.	Done.
Page 14: Reference 53 must be reference 54.	The reference number moved to #57
Page 14: Reference 54 must be reference 55 (revision of Brevitrygon).	The reference number moved to #45
Page 14: Reference 11 is incorrect for revision of Neotrygon, it must be the following paper:<br><br>Last, P.R. & White, W.T. & Séret, B. (2016) Taxonomic status of maskrays of the Neotrygon kuhlii species complex (Myliobatoidei: Dasyatidae) with the description of three new species from the Indo-West Pacific. Zootaxa, 4083(4), 533–561	The reference number of this articles is now #47.
Page 14: Reference 55 must be reference 56.	The reference number moved to #58
Page 14: Reference 56-58 must be references 57-59.	Done.
Pages 14-15: You state that B. hispidus, B. stewarti, B. naylori, B. dawsoni, and B. canescens are found in the same region (Thai Andaman Sea). However, only B. hispidus has been found in the Andaman Sea and the paper cited for B. hispidus (reference 60) is about specimens collected from off Southwest India, not the Andaman Sea. Specimens of B. hispidus from the Andaman Sea were described in the following references:<br><br>Alcock, A. (1891) Class Pisces. In: II.–Natural history notes from H.M. Indian marine survey steamer 'Investigator', Commander R.F. Hoskyn, R.N. commanding.–Series II., No. 1. On the results of deep-sea dredging during the season 1890–91. The Annals and Magazine of Natural History, 8 (6), 16–34.<br><br>Springer, S. & D'Aubrey, J.D. (1972) Two New Scyliorhinid Sharks from the East Coast of Africa with Notes on Related Species. Oceanographic Research Institute, Investigational Report, 29, 1–19.	"You state that B. hispidus, B. stewarti, B. naylori, B. dawsoni, and B. canescens are found in the same region (Thai Andaman Sea)." → This is **<u>NOT</u>** what we stated.<br>We tried to say that we compared morphological characteristics of our samples with known Bythaelurus spp. The only known Bythaelurus is our study locations is B. hispidus. The word "region" in this case is very broad including all the Indian Ocean and Indo-Pacific region since this genus is not widely distributed. If we only review a few species, we would have been criticized for missing others.<br><br>To clarify this misunderstanding, we revise a sentence as following:<br><br>Identification based on morphological characteristics revealed that all examined samples collected from the Thai Andaman Sea were of B. lutarius [5]. This species, first described as Halaelurus lutarius [62], is clearly distinguishable from similar looking species in the same genus [63-67].

Reviewers' comments	Response
Springer, S. (1979) A revision of the catsharks, family Scyliorhinidae. NOAA Technical Report NMFS Circular, 422, 1–152.<br><br>Séret, B. (1987) Halaelurus clevai, sp. n., a new species of catshark (Scyliorhinidae) from off Madagascar, with remarks on the taxonomic status of the genera Halaelurus Gill & Galeus Rafinesque. J.L.B. Smith Institute of Ichthyology Special Publication, 44, 1–27.<br><br>Kaschner, C.J., Weigmann, S. & Thiel, R. (2015) Bythaelurus tenuicephalus n. sp., a new deep-water catshark (Carcharhiniformes, Scyliorhinidae) from the western Indian Ocean. Zootaxa, 4013 (1), 120–138. http://dx.doi.org/10.11646/zootaxa.4013.1.9<br><br>Weigmann S, Ebert DA, Clerkin PJ, Stehmann MF, Naylor GJ. Bythaelurus bachi n. sp., a new deep-water catshark (Carcharhiniformes, Scyliorhinidae) from the southwestern Indian Ocean, with a review of Bythaelurus species and a key to their identification. Zootaxa. 2016; 4208(5): 401–432. https://doi.org/10.11646/zootaxa.4208.5.1<br><br>Weigmann S, Kaschner CJ. Bythaelurus vivaldii, a new deep-water catshark (Carcharhiniformes, Scyliorhinidae) from the northwestern Indian Ocean off Somalia. Zootaxa. 2017; 4263(1): 97–119. https://doi.org/10.11646/zootaxa.4263.1.4<br><br>Weigmann S, Kaschner CJ, Thiel R. A new microendemic species of the deep-water catshark genus Bythaelurus (Carcharhiniformes, Pentanchidae) from the northwestern Indian Ocean, with investigations of its feeding ecology, generic review and identification key. PLoS One. 2018;13(12):e0207887.	We did not include Alcock 1891 and Séret 1987 because we did not use them.
The next sentence is not correct, either, as ND2 sequences for B. hispidus were published in the following paper:<br><br>Weigmann S, Ebert DA, Clerkin PJ, Stehmann MF, Naylor GJ. Bythaelurus bachi n. sp., a new deep-water catshark (Carcharhiniformes,	ND2 of B. hispidus is NOT available for public assessment. Therefore, it cannot be used for reference.<br>This is a problem found in many species as **some authors did not share** information to public.<br><br>We revised the sentence as following:

Reviewers' comments	Response
Scyliorhinidae) from the southwestern Indian Ocean, with a review of Bythaelurus species and a key to their identification. Zootaxa. 2016; 4208(5): 401–432. https://doi.org/10.11646/zootaxa.4208.5.1	The lack of COI sequences of B. lutarius from the type locality and the unavailability of the ND2 sequence of B. hispidus, despite being studied [65], poses a challenge to the use of DNA barcoding for species confirmation.
Tentative identification should be indicated by writing B. cf. lutarius (with cf. not written in italics) and likewise also for other spp. with tentative identification.	Done.

Reviewer #2

Reviewers’ comments	Response
**Abstract**<br>"mean for species identification" should be "means of species identification."	Edited as suggested
"exercise cautions" should be "exercise caution."	Edited as suggested
**Methodological Clarifications**<br>• Mention the primers used in this study	Lists of primers used in this study in addition to the universal primers (Ward et al. 2005) are provided in the Supplementary section.
• DNA quantification method: Mention how DNA concentration was measured (e.g., Nanodrop, Qubit?).	More explanation as following:<br>The quality of the extracted DNA samples was assessed by electrophoresis on a 1% agarose gel. Their concentrations were measured using a Nanodrop (NanoVue^{TM}).
• Threshold justification: Explain why 98% identity and 95% coverage were chosen as cutoffs (e.g., precedent in literature).	This justification is explained as following:<br>The 95% query coverage threshold was selected for its robustness and greater likelihood of yielding accurate matches. A similarity level of 98% or higher was chosen because intraspecific genetic variation in most fish species studied to date typically falls within this range [13, 15, 20–22].
• *Glaucostegus* exception: The lowering of query coverage to 60% should be explained more clearly is this due to database fragment length?	The DNA quality of the *Glaucostegus* samples was very poor, requiring the design of new primers for targeted amplification. However, the resulting sequences did not align well with published reference sequences. The best alignment achieved was approximately 60% coverage with the available data.<br>Revision:<br>Nevertheless, COI fragments from our samples did not overlap with most *Glaucostegus* COI sequences in the public database due to very poor DNA quality resulting in sequences that did not align well with published reference sequences, preventing the application of a 95% query coverage threshold. Instead, a minimum coverage of 60% or higher was used to achieve the best possible results.
• Model selection: The model used for phylogenetics (TIM2+F+I+G4) is specified, but the alignment software used to generate sequence alignments is not mentioned.	A sentence for more explanation was added:<br>To assess relationships between the samples and reference data, phylogenetic trees were constructed. Sequences were aligned in MEGA 11 and saved as FASTA files.
• Outgroups: No mention of which outgroups were used (if any) in tree construction — important for rooting and interpretation.	Adding explanation:<br>For COI analysis in sharks, sequences of *Rhynchobatus australiae* and *Glaucostegus granulatus* served as outgroups, while that of *R. australiae* was used for ND2. In batoids, sequences of *Chiloscyllium punctatum* and *C.*

Reviewers' comments	Response
	griseum were used as outgroups for COI and ND2, respectively.
• Voucher information: Though storage locations are mentioned, include how voucher IDs were assigned and whether they are linked to GenBank entries.	Voucher IDs were assigned by the respective collectors, as the samples are kept in multiple institutions. During NCBI sequence submission, contributors are required to provide as much sample information as possible. All authors agreed to use the voucher IDs designated by their institutions, which will appear in the database once the sequences are published and released.
The study relies heavily on publicly available databases (BOLD, NCBI), many of which contain mislabeled or outdated entries. This issue is discussed, but the authors might consider emphasizing the importance of curating verified local reference libraries.	We provided more discussion in the last paragraph of the Discussion section.<br>However, since taxonomic revisions are ongoing and updates may not always keep pace, maintaining well-curated voucher libraries and local reference collections is essential for accurate species verification.
**The Results and Discussion**<br>• Consider separating detailed species-level data into Supplementary Materials. Use summary tables/figures for clarity (e.g., show cases of taxonomic conflict or cryptic species with quick-reference tables).	We would like to keep Table 2 in the Result section because it illustrates and summarized our results as we planned.<br>The supplementary materials (appendix B) showed detailed information for each sample used in this study.

Reviewer #3

Reviewers' comments	Response
The work presents some spelling errors that the authors need to correct to allow for better understanding. For example, many sections on page 17 present a lot of redundancy and this is repeated in some other places. The lack of line numbering in the manuscript makes it difficult to provide better guidance, in this case I suggest that authors have access to the PDF to see the other comments.	Please see below. We try to solve your comments/ questions point-by-point.
Include a brief discussion regarding the high percentage of divergence within the species Himantura leoparda and Narcine spp.	References that explained cryptic diversity of *Himantura leoparda* (*Himantura uarnak* species complex) [Arlyza et al. 2013], *Narcine* spp. (taxonomic uncertainty in the order Torpediniformes, and *Maculabatis gerrardi* complex [Muhala et al. 2024] were added.
Marks on pdf<br>Title → The DNA barcoding is already stablished and well know, why do you want to observe the limitation and advantages?	We would like to observe the advantages and limitations of DNA barcoding because we have a lot of problems assessing biodiversity of cartilaginous fishes in our studied locations. Several taxonomists do not believe our identification because we are not experts. When we try to use genetics to help with our identification, they do not believe in using molecular markers for identification.<br>A lot of classical taxonomists use "morphological species concept" to identify species not molecular characteristics.<br>We think that they are true at a certain level, but morphologically distinct species should also be genetically distinct. The question is by how much.<br>Because of this question, we think using genetic data may have some limitations. So, we explore our resources trying to list some limitations of DNA barcoding.
**Abstract**<br>In this portion doesent sound well if we aare in a methodology section. May be you should have wrote the number of specimes evaluated and them in the results section metion the number of species for each group of species.	Abstract editing:<br>In this study, DNA products from 153 elasmobranch samples were amplifiable and revealed a total of 28 shark species and 32 batoid species.<br><br>The number of specimens with successful amplification was mentioned in the Result section.

Reviewers' comments	Response
**Introduction**<br>In think that before putting the sampling technics, the author could bring the sampling aerea ou study area location and then bring the remain information.	We added some words into this sentence: "Nonetheless, as Thailand has an urgent need for improved the country's biodiversity assessment, the adoption of DNA barcoding is a worthwhile endeavor."<br>Studied locations are along the Gulf of Thailand and the Andaman Sea. Samples came from several sources as stated in the Method section.
secondly??	To make it clear, we can take the word "primarily" out.<br>This research's objectives were to evaluate the extent of ………….
In the **methodologies** chapter, there is a portion that must be reconstructed, it is results information.	Please see below
**Methods**<br>Bold the sub-section	Done.
Redundance<br>The identification process relied on the guidance provided in Krajangdara et al. [5], Last et al. [34], Krajangdara [35], and Ebert et al. [36].	We shorten this sentence by combining with the previous sentence as following:<br><br>These specimens were photographed and underwent subsequent species identification by T. Krajangdara following the guidance provided in Krajangdara et al. [5], Last et al. [34], Krajangdara [35], and Ebert et al. [36].
"cf."<br>Any information for this designation??? or its just a simbol withouth meaning.	"cf." stands for the Latin word "confer" meaning compare. It is used when a specimen looks similar but not identical to a known species. We use it when we were not sure what that specimen was.
volume → total volume	Done.
BOLD → BOLD system	Done.
the Himantura genera is now recoganized as Maculabatis	The name "Himantura" is split into several genera, but still used in some species e.g. *H. leoparda*.<br>In the sentence "The differences in the generic names of species in the genera previously named as *Himantura* and *Dasyatis*, were not treated as different from the newly assigned names", we try to say that the name (for example) *Himantura uarnacoides* is the same as *Pateobatis uarnacoides*. Both names came up in the GenBank database.

Reviewers' comments	Response
This portion seems redundance, the author can join this information regarding the utilization of these two tools.<br>This methodology utilized the BLAST algorithm within NCBI, as well as the Identification Engine embedded in BOLDSYSTEMS.	We shorten the sentence as following:<br><br>This methodology utilized the BLAST algorithm within (NCBI) and BOLD Identification Engine.
This Portion seems part of the results.<br>Nevertheless, COI fragments from our samples did not overlap with most *Glaucostegus* COI sequences in the public database, preventing the application of a 95% query coverage threshold. Instead, a minimum coverage of 60% or higher was used.	We have thought about this issue, too, and decided to talk about it in the Method section because we described the way we worked here.
redundance<br>the best substitution model	We removed "best substitution" out as following:<br><br>The model for the sequence alignment was selected using…….
edited also<br>generated and visualized	We removed 'and visualized' out as following:<br><br>The consensus tree was generated by Figtree…..
**Results**<br>Table 2 → improve<br>Table 2 needs to be improved to allow clarity, although we are faced with a comparison between the two markers, I suggest that they be in different columns and that they include the voucher or codes from GENBANK or Bold System if they were submitted. Alias needs to make it clear that this data was submitted to these platforms.	We have tried to make Table 2 several versions. The version presented in this draft is better than others.<br>We try to show the identification results from different techniques. Then, we make a consensus species and identify problems.<br>The full results of each specimen were provided in the supplementary section.
Why the authors join both markers here??	We join both markers because several samples could NOT be amplified from both markers. ND2 was more difficult to amplify than COI.<br>As we want to present species identification using DNA barcoding regardless of markers, we join the results from both marker into 1 column to compare with morphological and phylogenetic examinations.
The Himantura genera should be revised considering the new placement. (Maculabatis).	We are not sure what you mean.<br>The genus Himantura has been split into several genera including Brevitrygon, Fluvitrygon, Fontitrygon, Maculabatis, Pateobatis, Styracura, and Urogymnus (Last et al. 2016)<br><br>Last PR, Naylor GJP, Manjaji-Matsumoto BM. A revised classification of the family Dasyatidae

Reviewers' comments	Response
	(Chondrichthyes: Myliobatiformes) based on new morphological and molecular insights. Zootaxa. 2016a;4139(3):345–368
phrase construction error. Please rewrite the sentence.<br>Genetic data for some species are newly described such as *Rhinobatos ranongensis* were not available for public access.	We re-write it as following:<br><br>Genetic data for some species such as *Rhinobatos ranongensis* were not available for public access.
I suggest to uniformize the writing of the species names.<br>*Chiloscyllium hasseltii*, which closely resembles *C. griseum*; *Carcharhinus falciformis*, misidentified as *C. brevipinna; C. sorrah*, misidentified as *C. seali*; and *Maculabatis gerrardi*, occasionally misidentified as *M. macrura*. These	 It is very confusing to use abbreviation for the generic names of elasmobranchs as many species begin with the same alphabet. In general, elasmobranch taxonomists are familiar with specific epithets. They tend to say the epithets and simultaneously know the generic names.<br>We are not sure if we uniform the writing of the species name in this draft will confuse the taxonomists.<br>So, we would like to keep it this way. However, we can spell out the full generic names in this sentence.
Not only this carachteristics.... See Muhala et al., 2024.<br>Similarly, *M. gerrardi* and *M. macrura* both exhibit white spots on their bodies but differ in the distribution of these markings.	 There are many morphological characteristics describing species in Maculabatis. In this case, we use a dichotomous key that differentiate Maculabatis found in Thai waters.<br>These 2 species are the most similar-looking pair and roughly differentiated by the distribution of white spots. Other characters are difficult to see. We also measured their morphometric characteristics, and so added these characteristics to the description as following:<br>Similarly, *M. gerrardi* and *M. macrura* both exhibit white spots on their bodies but differ in the distribution of these markings and the morphometric ratios (head length to disc width and disc length to disc width).
Narcine spp.	Re-write:<br>*Narcine* species
Past tense<br>did not aligned	Done
how did you do in this scenario whre you could not definitely stablish or identify the specie??<br>not be definitively established	 For this species, we revised the consensus to *B.* cf. *lutarius* because the other reviewer

Reviewers' comments	Response
	disagreed that *B. lutarius* could be found in the Thai Andaman Sea.
what did you consider in this cases?? and did you use any specific analyses to validade??<br><br>The COI sequence of this sample did not match any references in both NCBI and BOLD while its ND2 sequence matched an *E. splendidus* sample from Taiwan.	In this *Etmopterus* case, we used geographic distribution of species in this genus and morphological characteristics.<br>Our specimen did not look like *E. splendidus* but highly similar to *E. fusus*. Considering geographic distribution of these species, this specimen was likely *E. fusus*.<br>The genetic data were not much useful as taxonomy of this genus is still confusing.
is this bases on the lengh of the sequence>??<br>based on 659 bp.	Yes.
better to use both less intraspecific variation species.<br>In sharks, the highest COI intraspecific variation was observed between our *Etmopterus fusus* sample and the NCBI reference, while the greatest ND2 variation was observed in our *Echinorhinus brucus* sample and the reference.	Re-write:<br>In sharks, the highest COI variation was between our *Etmopterus fusus* sample and references. A similar ND2 pattern was seen in *Echinorhinus brucus*.
do you disscuss this??<br>Additionally, *Himantura leoparda* and *Narcine* spp. showed COI intraspecific<br>variation exceeding 4%.	We added more discussion on this.
Do you mean distance???<br>Table 3<br>Variations among…	Yes
Is tis a point???<br>*Neotrygon kuhlii* and other *Neotrygon* spp. and	Re-write<br>*Neotrygon kuhlii* and other *Neotrygon* species
Phylogenetic trees need to be reconstructed or better edited, they are not clear and the species evidenced in the study, especially the groups, need to have some signaling.	We edited the tree.
**Discussion**<br>Number of references	There are some mis-uses of punctuation in numbering references. We have corrected and re-numbered these references.
This problem is also observed in Maculabatis gerrard complex.. There are always problems in identifying the species.<br>For example, DNA barcodes of samples identified as *Centrophorus uyato* have been matched with different *Centrophorus* species due to recent revisions that revealed *C. granulosus* as a species complex.	Add a sentence and ref.<br><br>……… due to recent revisions that revealed *C. granulosus* as a species complex. This problem is also observed in Maculabatis gerrardi complex [57].
in general or for specific species or genera??<br>On the contrary, information on ND2 sequences is more recent and up-to-date serving as a better	in general or for specific species or genera?? → in general<br>re-write

Reviewers’ comments	Response
reference than COI.	On the contrary, information on ND2 sequences is generally more recent and up-to-date serving as a better reference than COI.
Are these species or genus?<br>exemplified by *Brevitrygon*<br>[38, 54], *Neotrygon* [11], and *Telatrygon* [55]	They are generic names in the family Dasyatidae.
you should write,. The species belonging to this genus exhibit .... this is more clear when mention a taxonomic classification<br>These species exhibit….	Re-write<br><br>These species belonging to these genera exhibit distinct morphological……….
Agree. Again see https://doi.org/10.1017/S0025315424000973 for Maculabatis gerrard complex that have also their geographic range extended to Mzambique channel<br>Some species are thought to be endemic to certain geographic locations, but their distribution is still unclear and requires further examination, which may result in further taxonomic revision	Yes
Reconstruct the sentence<br>The clear genetic differentiation between<br>*M. stevensi* and *M. lenticulatus* [10], which allowed a correct consensus identification.	Re-write<br><br>The clear genetic differentiation between *M. stevensi* and *M. lenticulatus* [10] enabled accurate consensus identification.
https://doi.org/10.1017/S0025315424000973<br>*montalbani*, *Maculabatis gerrardi*,<br>and *Narcine timlei*, display high genetic variation within species.	Add a reference<br><br>This may arise from either cryptic diversity or misidentification of reference samples as seen in the species complex of *Himantura uarnak* [71], *Maculabatis gerrardi* [57], and *Narcine* species. [72].
Reference<br>….for understanding these processes.	 ….for understanding these processes [80].
