# Supplementary material for: DNA barcoding for elasmobranch diversity assessment in Thailand: Its advantages and limitations
Source: PLoS One. 2025 Oct 31;20(10):e0334640. doi: 10.1371/journal.pone.0334640 (PMC12578239; doi:10.1371/journal.pone.0334640)
Supplement: Attachment — Submitted filename: Response to reviewers.docx [file pone.0334640.s006.docx]

Letter of response to reviewer’s comments

- Adding the section “Financial disclosure”
- Move the ethic statements (previously in the Acknowledgements) to method section
- Separate supporting information into 2 files: S1—primers and protocol used, S2—sample identification including morphological and genetic examination of each sample.

Reviewer #1

| Reviewers’ comments | Response |
| --- | --- |
| Nevertheless, if the morphology was indeed examined, it needs to be explained how it was examined and the morphological data should be presented in the manuscript. | Morphological identification of ALL samples is in the supplementary section II. The identification was on site. Some uncertain individuals were brought to the lab for thorough examination. If identification was uncertain, they were marked as unsure. |
| Page 2, sentence “Available genetic information, though scant, poses many questions regarding taxonomic diversity in which the high levels of genetic differentiation that could not be ignored [11]”: This message was not published in the cited paper but in the reply to that paper. Therefore, the reference needs to be changed to the following one:  Weigmann 2017: Reply to Borsa (2017): Comment on ‘Annotated checklist of the living sharks, batoids and chimaeras (Chondrichthyes) of the world, with a focus on biogeographical diversity by Weigmann (2016)’. Journal of Fish Biology, 90(4), 1176–1181. | We felt that Weigmann 2017 Reply to Borsa 2017 appeared to place priority to morphology rather than genetics, and take genetics as “not important” or something that can be ignored.  However, if the reviewer feels that we should cite Weigmann 2017, we would do so. |
| Table 2: The correct name of the order is “CARCHARHINIFORMES” | Done. |
| Page 7, Iago mangalorensis: The specimens should also be compared with the very recently described Iago gopalakrishnani. | We used the name *Iago mangalorensis* before the knowledge of this publication by the time of manuscript submission.  We changed the name and provided the citation as suggested.  The edited version moves *Iago mangalorensis* to #2 Taxonomic revision/ redescription/ new species assignments. In table 2, the consensus species of this sample is changed to *I. gopalakrishnani*, but the phylogenetic tree remains unchanged as the genetic sequences of this new species are not available for public use. Only samples labelled as Iago sp. are. |
| Page 9, *2: Reference 42 is the paper currently listed as reference 43 in the reference section. | There are additional references being added. This reference is now #42 |
| Page 9, *4: Reference 43 is the paper currently listed as reference 42 in the reference section. | There are additional references being added. This reference is now #44 |
| Page 9, *5: Wrong paper cited, a taxonomic and biogeographic revision of Brevitrygon was carried out in the following paper:  Last, P. R., Weigmann, S., & Naylor, G. J. P. (2023). The Indo-Pacific Stingray Genus Brevitrygon (Myliobatiformes: Dasyatidae): Clarification of Historical Names and Description of a New Species, B. manjajiae sp. nov., from the Western Indian Ocean. Diversity, 15(12), 1213. <https://doi.org/10.3390/d15121213> | We would like to give credit to the original paper that described the genus *Brevitrygon*. We know that Last et al. (2023) provided a revision of species in this genus, but some information in GenBank referred to this genus as Himantura.  We put 2 references in Table 2. |
| Page 9, *6: A taxonomic revision of Neotrygon kuhlii was not carried out in the cited references but in the following reference: Last, P.R. & White, W.T. & Séret, B. (2016) Taxonomic status of maskrays of the Neotrygon kuhlii species complex (Myliobatoidei: Dasyatidae) with the description of three new species from the Indo-West Pacific. Zootaxa, 4083(4), 533–561 | Reference added. |
| Page 9, *7-9: Write “species resurrection” instead of “new species erection”. | Done |
| Page 10, reference 43: see my comment 6 (regarding Glaucostegus younholeei); same applies to mentioning of this reference on page 13. | Done |
| Page 13, first sentence of Discussion: This sentence gives the impression that barcoding can be used without making any morphological examinations. This is a very dangerous way as many examples from the literature have shown that solely relying on genetics can lead to clearly incorrect conclusions and even incorrect new species descriptions. One example for such wrong conclusions the following paper (see their Etmopterus lucifer in the order Carcharhiniformes):  Vélez-Zuazo X, Agnarsson I. Shark tales: a molecular species-level phylogeny of sharks (Selachimorpha, Chondrichthyes). Mol Phylogenet Evol. 2011 Feb;58(2):207-17. doi: 10.1016/j.ympev.2010.11.018.  The importance of integrating morphological and molecular taxonomies has been summarized, e.g., in the previously mentioned paper by Weigmann (2017) (see my comment 2 for the full citation of this paper). | Our intention does NOT mean that barcoding can be used without making any morphological examinations. We relied on morphological examination before the use of genetics. We use genetics to HELP with the identification when we are uncertain.  It is difficult to work in taxonomic studies that require long-term training and experiences. We know that there is a conflict between taxonomists and phylogeneticist.  The 2 references cited in this sentence (Cerutii-Pereyra et al. 2012 and Gostel and Kress 2022) just pointed out the usefulness of genetics to help with species identification.  We agree with the reviewer that using molecular data alone to identify species can lead to wrong conclusion. To clarify this sentence, we revised the sentence as following:  The implementation of DNA barcoding significantly enhances the taxonomic clarification of cartilaginous fishes, offering an alternative to the intricate and time-consuming process of species identification for non-taxonomists [15, 51]. However, morphological examination still remains the key method. |
| isPage 14: Reference 59 must be reference 52. | The reference number moved to #55. |
| Page 14: Reference 42 must be reference 43 and reference 52 must be reference 53. | Done. |
| Page 14: Reference 53 must be reference 54. | The reference number moved to #57 |
| Page 14: Reference 54 must be reference 55 (revision of Brevitrygon). | The reference number moved to #45 |
| Page 14: Reference 11 is incorrect for revision of Neotrygon, it must be the following paper:  Last, P.R. & White, W.T. & Séret, B. (2016) Taxonomic status of maskrays of the Neotrygon kuhlii species complex (Myliobatoidei: Dasyatidae) with the description of three new species from the Indo-West Pacific. Zootaxa, 4083(4), 533–561 | The reference number of this articles is now #47. |
| Page 14: Reference 55 must be reference 56. | The reference number moved to #58 |
| Page 14: Reference 56-58 must be references 57-59. | Done. |
| Pages 14-15: You state that B. hispidus, B. stewarti, B. naylori, B. dawsoni, and B. canescens are found in the same region (Thai Andaman Sea). However, only B. hispidus has been found in the Andaman Sea and the paper cited for B. hispidus (reference 60) is about specimens collected from off Southwest India, not the Andaman Sea. Specimens of B. hispidus from the Andaman Sea were described in the following references:  Alcock, A. (1891) Class Pisces. In: II.–Natural history notes from H.M. Indian marine survey steamer ‘Investigator’, Commander R.F. Hoskyn, R.N. commanding.–Series II., No. 1. On the results of deep-sea dredging during the season 1890–91. The Annals and Magazine of Natural History, 8 (6), 16–34.  Springer, S. & D’Aubrey, J.D. (1972) Two New Scyliorhinid Sharks from the East Coast of Africa with Notes on Related Species. Oceanographic Research Institute, Investigational Report, 29, 1–19.  Springer, S. (1979) A revision of the catsharks, family Scyliorhinidae. NOAA Technical Report NMFS Circular, 422, 1–152.  Séret, B. (1987) Halaelurus clevai, sp. n., a new species of catshark (Scyliorhinidae) from off Madagascar, with remarks on the taxonomic status of the genera Halaelurus Gill & Galeus Rafinesque. J.L.B. Smith Institute of Ichthyology Special Publication, 44, 1–27.  Kaschner, C.J., Weigmann, S. & Thiel, R. (2015) Bythaelurus tenuicephalus n. sp., a new deep-water catshark (Carcharhiniformes, Scyliorhinidae) from the western Indian Ocean. Zootaxa, 4013 (1), 120–138. <http://dx.doi.org/10.11646/zootaxa.4013.1.9>  Weigmann S, Ebert DA, Clerkin PJ, Stehmann MF, Naylor GJ. Bythaelurus bachi n. sp., a new deep-water catshark (Carcharhiniformes, Scyliorhinidae) from the southwestern Indian Ocean, with a review of Bythaelurus species and a key to their identification. Zootaxa. 2016; 4208(5): 401–432. [https://doi.org/10.11646/zootaxa.4208.5.1](https://doi.org/10.11646/zootaxa.4208.5.1)  Weigmann S, Kaschner CJ. Bythaelurus vivaldii, a new deep-water catshark (Carcharhiniformes, Scyliorhinidae) from the northwestern Indian Ocean off Somalia. Zootaxa. 2017; 4263(1): 97–119. [https://doi.org/10.11646/zootaxa.4263.1.4](https://doi.org/10.11646/zootaxa.4263.1.4)  Weigmann S, Kaschner CJ, Thiel R. A new microendemic species of the deep-water catshark genus Bythaelurus (Carcharhiniformes, Pentanchidae) from the northwestern Indian Ocean, with investigations of its feeding ecology, generic review and identification key. PLoS One. 2018;13(12):e0207887. | “You state that B. hispidus, B. stewarti, B. naylori, B. dawsoni, and B. canescens are found in the same region (Thai Andaman Sea).” 🡪 This is **NOT** what we stated.  We tried to say that we compared morphological characteristics of our samples with known Bythaelurus spp. The only known Bythaelurus is our study locations is B. hispidus. The word “region” in this case is very broad including all the Indian Ocean and Indo-Pacific region since this genus is not widely distributed. If we only review a few species, we would have been criticized for missing others.  To clarify this misunderstanding, we revise a sentence as following:  Identification based on morphological characteristics revealed that all examined samples collected from the Thai Andaman Sea were of B. lutarius [5]. This species, first described as Halaelurus lutarius [62], is clearly distinguishable from similar looking species in the same genus [63-67].  We did not include Alcock 1891 and Séret 1987 because we did not use them. |
| The next sentence is not correct, either, as ND2 sequences for B. hispidus were published in the following paper:  Weigmann S, Ebert DA, Clerkin PJ, Stehmann MF, Naylor GJ. Bythaelurus bachi n. sp., a new deep-water catshark (Carcharhiniformes, Scyliorhinidae) from the southwestern Indian Ocean, with a review of Bythaelurus species and a key to their identification. Zootaxa. 2016; 4208(5): 401–432. <https://doi.org/10.11646/zootaxa.4208.5.1> | ND2 of B. hispidus is NOT available for public assessment. Therefore, it cannot be used for reference.  This is a problem found in many species as **some authors did not share** information to public.  We revised the sentence as following:  The lack of COI sequences of B. lutarius from the type locality and the unavailability of the ND2 sequence of B. hispidus, despite being studied [65], poses a challenge to the use of DNA barcoding for species confirmation. |
| Tentative identification should be indicated by writing B. cf. lutarius (with cf. not written in italics) and likewise also for other spp. with tentative identification. | Done. |

Reviewer #2

| Reviewers’ comments | Response |
| --- | --- |
| **Abstract** "mean for species identification" should be "means of species identification." | Edited as suggested |
| "exercise cautions" should be "exercise caution." | Edited as suggested |
| **Methodological Clarifications** • Mention the primers used in this study | Lists of primers used in this study in addition to the universal primers (Ward et al. 2005) are provided in the Supplementary section. |
| • DNA quantification method: Mention how DNA concentration was measured (e.g., Nanodrop, Qubit?). | More explanation as following:  The quality of the extracted DNA samples was assessed by electrophoresis on a 1% agarose gel. Their concentrations were measured using a Nanodrop (NanoVue^TM^). |
| • Threshold justification: Explain why 98% identity and 95% coverage were chosen as cutoffs (e.g., precedent in literature). | This justification is explained as following:  The 95% query coverage threshold was selected for its robustness and greater likelihood of yielding accurate matches. A similarity level of 98% or higher was chosen because intraspecific genetic variation in most fish species studied to date typically falls within this range [13, 15, 20–22]. |
| • *Glaucostegus* exception: The lowering of query coverage to 60% should be explained more clearly is this due to database fragment length? | The DNA quality of the *Glaucostegus* samples was very poor, requiring the design of new primers for targeted amplification. However, the resulting sequences did not align well with published reference sequences. The best alignment achieved was approximately 60% coverage with the available data.  Revision:  Nevertheless, COI fragments from our samples did not overlap with most *Glaucostegus* COI sequences in the public database due to very poor DNA quality resulting in sequences that did not align well with published reference sequences, preventing the application of a 95% query coverage threshold. Instead, a minimum coverage of 60% or higher was used to achieve the best possible results. |
| • Model selection: The model used for phylogenetics (TIM2+F+I+G4) is specified, but the alignment software used to generate sequence alignments is not mentioned. | A sentence for more explanation was added:  To assess relationships between the samples and reference data, phylogenetic trees were constructed. Sequences were aligned in MEGA 11 and saved as FASTA files. |
| • Outgroups: No mention of which outgroups were used (if any) in tree construction — important for rooting and interpretation. | Adding explanation:  For COI analysis in sharks, sequences of *Rhynchobatus australiae* and *Glaucostegus granulatus* served as outgroups, while that of *R. australiae* was used for ND2. In batoids, sequences of *Chiloscyllium punctatum* and *C. griseum* were used as outgroups for COI and ND2, respectively. |
| • Voucher information: Though storage locations are mentioned, include how voucher IDs were assigned and whether they are linked to GenBank entries. | Voucher IDs were assigned by the respective collectors, as the samples are kept in multiple institutions. During NCBI sequence submission, contributors are required to provide as much sample information as possible. All authors agreed to use the voucher IDs designated by their institutions, which will appear in the database once the sequences are published and released. |
| The study relies heavily on publicly available databases (BOLD, NCBI), many of which contain mislabeled or outdated entries. This issue is discussed, but the authors might consider emphasizing the importance of curating verified local reference libraries. | We provided more discussion in the last paragraph of the Discussion section.  However, since taxonomic revisions are ongoing and updates may not always keep pace, maintaining well-curated voucher libraries and local reference collections is essential for accurate species verification. |
| **The Results and Discussion**   - Consider separating detailed species-level data into Supplementary Materials. Use summary tables/figures for clarity (e.g., show cases of taxonomic conflict or cryptic species with quick-reference tables). | We would like to keep Table 2 in the Result section because it illustrates and summarized our results as we planned.  The supplementary materials (appendix B) showed detailed information for each sample used in this study. |

Reviewer #3

| Reviewers’ comments | Response |
| --- | --- |
| The work presents some spelling errors that the authors need to correct to allow for better understanding. For example, many sections on page 17 present a lot of redundancy and this is repeated in some other places. The lack of line numbering in the manuscript makes it difficult to provide better guidance, in this case I suggest that authors have access to the PDF to see the other comments. | Please see below. We try to solve your comments/ questions point-by-point. |
| Include a brief discussion regarding the high percentage of divergence within the species Himantura leoparda and Narcine spp. | References that explained cryptic diversity of *Himantura leoparda* (*Himantura uarnak* species complex) [Arlyza et al. 2013], *Narcine* spp. (taxonomic uncertainty in the order Torpediniformes, and *Maculabatis gerrardi* complex [Muhala et al. 2024] were added. |
| Marks on pdf  Title 🡪 The DNA barcoding is already stablished and well know, why do you want to observe the limitation and advantages? | We would like to observe the advantages and limitations of DNA barcoding because we have a lot of problems assessing biodiversity of cartilaginous fishes in our studied locations.  Several taxonomists do not believe our identification because we are not experts. When we try to use genetics to help with our identification, they do not believe in using molecular markers for identification.  A lot of classical taxonomists use “morphological species concept” to identify species not molecular characteristics.  We think that they are true at a certain level, but morphologically distinct species should also be genetically distinct. The question is by how much.  Because of this question, we think using genetic data may have some limitations. So, we explore our resources trying to list some limitations of DNA barcoding. |
| **Abstract**  In this portion doesent sound well if we aare in a methodology section. May be you should have wrote the number of specimes evaluated and them in the results section metion the number of species for each group of species. | Abstract editing:  In this study, DNA products from 153 elasmobranch samples were amplifiable and revealed a total of 28 shark species and 32 batoid species.  The number of specimens with successful amplification was mentioned in the Result section. |
| **Introduction**  In think that before putting the sampling technics, the author could bring the sampling aerea ou study area location and then bring the remain information. | We added some words into this sentence:  “Nonetheless, as Thailand has an urgent need for improved the country’s biodiversity assessment, the adoption of DNA barcoding is a worthwhile endeavor.”  Studied locations are along the Gulf of Thailand and the Andaman Sea. Samples came from several sources as stated in the Method section. |
| secondly?? | To make it clear, we can take the word “primarily” out.  This research's objectives were to evaluate the extent of ………….. |
| In the **methodologies** chapter, there is a portion that must be reconstructed, it is results information. | Please see below |
| **Methods**  Bold the sub-section | Done. |
| Redundance  The identification process relied on the guidance  provided in Krajangdara et al. [5], Last et al. [34], Krajangdara [35], and Ebert et al. [36]. | We shorten this sentence by combining with the previous sentence as following:  These specimens were photographed and underwent subsequent species identification by T. Krajangdara following the guidance provided in Krajangdara et al. [5], Last et al. [34], Krajangdara [35], and Ebert et al. [36]. |
| “cf.”  Any information for this designation??? or its just a simbol withouth meaning. | “cf.” stands for the Latin word “confer” meaning compare. It is used when a specimen looks similar but not identical to a known species. We use it when we were not sure what that specimen was. |
| volume 🡪 total volume | Done. |
| BOLD 🡪 BOLD system | Done. |
| the Himantura genera is now recoganized as Maculabatis | The name “Himantura” is split into several genera, but still used in some species e.g. *H. leoparda*.  In the sentence “The differences in the generic names of species in the genera previously named as *Himantura* and *Dasyatis*, were not treated as different from the newly assigned names”, we try to say that the name (for example) *Himantura uarnacoides* is the same as *Pateobatis uarnacoides*. Both names came up in the GenBank database. |
| This portion seems redundance, the author can join this information regarding the utilization of these two tools.  This methodology utilized the BLAST algorithm within NCBI, as well as the Identification Engine embedded in BOLDSYSTEMS. | We shorten the sentence as following:  This methodology utilized the BLAST algorithm within (NCBI) and BOLD Identification Engine. |
| This Portion seems part of the results.  Nevertheless, COI fragments from our  samples did not overlap with most *Glaucostegus* COI sequences in the public database,  preventing the application of a 95% query coverage threshold. Instead, a minimum coverage of 60% or higher was used. | We have thought about this issue, too, and decided to talk about it in the Method section because we described the way we worked here. |
| redundance  the best substitution model | We removed “best substitution” out as following:  The model for the sequence alignment was selected using……. |
| edited also  generated and visualized | We removed ‘and visualized’ out as following:  The consensus tree was generated by Figtree….. |
| **Results**  Table 2 🡪 improve  Table 2 needs to be improved to allow clarity, although we are faced with a comparison between the two markers, I suggest that they be in different columns and that they include the voucher or codes from GENBANK or Bold System if they were submitted. Alias needs to make it clear that this data was submitted to these platforms. | We have tried to make Table 2 several versions. The version presented in this draft is better than others.  We try to show the identification results from different techniques. Then, we make a consensus species and identify problems.  The full results of each specimen were provided in the supplementary section. |
| Why the authors join both markers here?? | We join both markers because several samples could NOT be amplified from both markers. ND2 was more difficult to amplify than COI.  As we want to present species identification using DNA barcoding regardless of markers, we join the results from both marker into 1 column to compare with morphological and phylogenetic examinations. |
| The Himantura genera should be revised considering the new placement. ( Maculabatis). | We are not sure what you mean.  The genus Himantura has been split into several genera including Brevitrygon, Fluvitrygon, Fontitrygon, Maculabatis, Pateobatis, Styracura, and Urogymnus (Last et al. 2016)  Last PR, Naylor GJP, Manjaji-Matsumoto BM. A revised classification of the family Dasyatidae (Chondrichthyes: Myliobatiformes) based on new morphological and molecular insights. Zootaxa. 2016a;4139(3):345–368 |
| phrase construction error. Please rewrite the sentence.  Genetic data for some species are newly described such as *Rhinobatos ranongensis* were not available for public access. | We re-write it as following:  Genetic data for some species such as *Rhinobatos ranongensis* were not available for public access. |
| I suggest to uniformize the writing of the species names.  *Chiloscyllium hasseltii*, which closely resembles *C.*  *griseum*; *Carcharhinus falciformis*, misidentified as *C. brevipinna; C. sorrah*, misidentified as *C.*  *seali*; and *Maculabatis gerrardi*, occasionally misidentified as *M. macrura*. These | It is very confusing to use abbreviation for the generic names of elasmobranchs as many species begin with the same alphabet. In general, elasmobranch taxonomists are familiar with specific epithets. They tend to say the epithets and simultaneously know the generic names.  We are not sure if we uniform the writing of the species name in this draft will confuse the taxonomists.  So, we would like to keep it this way. However, we can spell out the full generic names in this sentence. |
| Not only this carachteristics.... See Muhala et al., 2024.  Similarly, *M. gerrardi* and *M. macrura* both exhibit white spots on their bodies but differ in the distribution of these markings. | There are many morphological characteristics describing species in Maculabatis. In this case, we use a dichotomous key that differentiate Maculabatis found in Thai waters.  These 2 species are the most similar-looking pair and roughly differentiated by the distribution of white spots. Other characters are difficult to see. We also measured their morphometric characteristics, and so added these characteristics to the description as following:  Similarly, *M. gerrardi* and *M. macrura* both exhibit white spots on their bodies but differ in the distribution of these markings and the morphometric ratios (head length to disc width and disc length to disc width). |
| *Narcine* spp. | Re-write:  *Narcine* species |
| Past tense  did not aligned | Done |
| how did you do in this scenario whre you could not definitely stablish or identify the specie??  not be definitively established | For this species, we revised the consensus to *B.* cf. *lutarius* because the other reviewer disagreed that *B. lutarius* could be found in the Thai Andaman Sea. |
| what did you consider in this cases?? and did you use any specific analyses to validade??  The COI sequence of this sample did not match any references in both NCBI and BOLD while its ND2 sequence matched an *E. splendidus* sample from Taiwan. | In this *Etmopterus* case, we used geographic distribution of species in this genus and morphological characteristics.  Our specimen did not look like *E. splendidus* but highly similar to *E. fusus*. Considering geographic distribution of these species, this specimen was likely *E. fusus*.  The genetic data were not much useful as taxonomy of this genus is still confusing. |
| is this bases on the lengh of the sequence>??  based on 659 bp. | Yes. |
| better to use both less intraspecific variation species.  In sharks, the highest COI intraspecific variation was observed between our *Etmopterus fusus* sample and the NCBI reference, while the greatest ND2 variation was observed in our *Echinorhinus brucus* sample and the reference. | Re-write:  In sharks, the highest COI variation was between our *Etmopterus fusus* sample and references. A similar ND2 pattern was seen in *Echinorhinus brucus*. |
| do you disscuss this??  Additionally, *Himantura leoparda* and *Narcine* spp. showed COI intraspecific  variation exceeding 4%. | We added more discussion on this. |
| Do you mean distance???  Table 3  Variations among… | Yes |
| Is tis a point???  *Neotrygon kuhlii* and other *Neotrygon* spp. and | Re-write  *Neotrygon kuhlii* and other *Neotrygon* species |
| Phylogenetic trees need to be reconstructed or better edited, they are not clear and the species evidenced in the study, especially the groups, need to have some signaling. | We edited the tree. |
| **Discussion**  Number of references | There are some mis-uses of punctuation in numbering references. We have corrected and re-numbered these references. |
| This problem is also observed in Maculabatis gerrard complex.. There are always problems in identifying the species.  For example, DNA barcodes of samples  identified as *Centrophorus uyato* have been matched with different *Centrophorus* species due to recent revisions that revealed *C. granulosus* as a species complex. | Add a sentence and ref.  ……… due to recent revisions that revealed *C. granulosus* as a species complex. This problem is also observed in Maculabatis gerrardi complex [57]. |
| in general or for specific species or genera??  On the contrary, information on ND2 sequences is more recent and up-to-date serving as a better  reference than COI. | in general or for specific species or genera?? 🡪 in general  re-write  On the contrary, information on ND2 sequences is generally more recent and up-to-date serving as a better reference than COI. |
| Are these species or genus?  exemplified by *Brevitrygon*  [38, 54], *Neotrygon* [11], and *Telatrygon* [55] | They are generic names in the family Dasyatidae. |
| you should write,. The species belonging to this genus exhibit ......... this is more clear when mention a taxonomic classification  These species exhibit…. | Re-write  These species belonging to these genera exhibit distinct morphological……….. |
| Agree. Again see https://doi.org/10.1017/S0025315424000973 for Maculabatis gerrard complex that have also their geographic range extended to Mzambique channel  Some species are thought to be endemic to certain geographic locations, but their distribution is still unclear and requires further examination, which may result in further taxonomic revision | Yes |
| Reconstruct the sentence  The clear genetic differentiation between  *M. stevensi* and *M. lenticulatus* [10], which allowed a correct consensus identification. | Re-write  The clear genetic differentiation between *M. stevensi* and *M. lenticulatus* [10] enabled accurate consensus identification. |
| <https://doi.org/10.1017/S0025315424000973>  *montalbani*, *Maculabatis gerrardi*,  and *Narcine timlei*, display high genetic variation within species. | Add a reference  This may arise from either cryptic diversity or misidentification of reference samples as seen in the species complex of *Himantura uarnak* [71], *Maculabatis gerrardi* [57], and *Narcine* species. [72]. |
| Reference  ….for understanding these processes. | ….for understanding these processes [80]. |
