# Supplementary material for: DNA barcoding for elasmobranch diversity assessment in Thailand: Its advantages and limitations
Source: PLoS One. 2025 Oct 31;20(10):e0334640. doi: 10.1371/journal.pone.0334640 (PMC12578239; doi:10.1371/journal.pone.0334640)
Supplement: Attachment — Submitted filename: Response to reviewers round 3.docx [file pone.0334640.s009.docx]

Response to reviewers

**Title:** DNA barcoding for elasmobranch diversity assessment in Thailand: its advantages and limitations

September 2025

Reviewer #2: The manuscript PONE-D-25-18018R1, entitled “DNA barcoding for elasmobranch diversity assessment in Thailand: its advantages and limitations,” is interesting; however, there are instances of redundancy, and some sentences could be rephrased for improved clarity and conciseness. Additionally, the absence of line numbers makes precise referencing challenging. Including line numbers would facilitate easier review and future revisions. Please ensure consistent formatting of species names (e.g., full genus names vs. abbreviations), particularly in the Results and Discussion sections.

**Reply:** We make a revision throughout to ensure clarity and consistency.

Abstract

"mean for species identification" should be "means of species identification."
"exercise cautions" should be "exercise caution."

**Reply:** Corrected.

Introduction

"Available genetic information, though scant, poses many questions regarding taxonomic diversity in which the high levels of genetic differentiation that could not be ignored [11]": The phrasing "in which the high levels of genetic differentiation that could not be ignored" is a bit clunky. Consider rephrasing for smoother reading.

**Reply:** Rephrased (Available genetic information, though scant, poses many questions regarding taxonomic diversity, particularly the high levels of genetic differentiation that cannot be overlooked).

Methods

• Did the authors perform sequence analysis? Which software did the authors use? Mention it in the ms!

**Reply:** included detailed analysis in the MM section (p. 3-4)

Results
• The authors' decision to keep Table 2 in the Results section as it illustrates and summarizes their findings is valid. The detailed information being in the supplementary section is also appropriate.
• The authors' preference for spelling out full generic names in the text to avoid confusion, especially given the similar initial letters of many elasmobranch genera, is a valid point. However, if the authors choose to abbreviate, ensure consistency (e.g., first use full name, then abbreviate).

Reply: Checked the consistency.

• addition of morphometric ratios to differentiate *M. gerrardi* and *M. macrura* provides more robust identification criteria.

**Reply:** *M. gerrardi* and *M. macrura* both exhibit white spots on their bodies but differ in the distribution of these markings and the morphometric ratios (head length to disc width and disc length to disc width) (p. 12)

• "did not aligned": This should be "did not align."

**Reply:** Corrected.

Reviewer #3: Congratulations on following the corrections left. The work is better. One thing could be added, but it doesn't take away any merit from this version. Congratulations.

**Reply:** Corrected both points (specified the range, p. 4, L. 155-156; and moved to the Results section p. 11, L. 261-265)

**Reply:**

Added the suggested reference in the discussion session (p. 15, L. 415).

We used existing literature to identify the species.

We are uncertain about the corrections of Table 2. We did some minor edits, like capitalization of the headings.
